# Supplementary material for: Strontium isotopes reveal diverse life history variations, migration patterns, and habitat use for Broad Whitefish (Coregonus nasus) in Arctic, Alaska
Source: PLoS One. 2022 May 2;17(5):e0259921. doi: 10.1371/journal.pone.0259921 (PMC9060380; doi:10.1371/journal.pone.0259921)
Supplement: S1 Table — Table displaying population sex, age, and size structure, tissues sampled, and sample size for Broad Whitefish (Coregonus nasus) caught at three locations within the Colville River watershed, Alaska, USA. (DOCX) [file pone.0259921.s001.docx]

**S1 Table**. **Summary of Broad Whitefish sampled.** Table displaying population structure, tissues sampled, and sample size for Broad Whitefish (*Coregonus nasus*) caught at three locations within the Colville River, AK, USA watershed.

Table legend. N= sample size, mm = millimeter, g = grams, yrs = years
