# Supplementary material for: Strontium isotopes reveal diverse life history variations, migration patterns, and habitat use for Broad Whitefish (Coregonus nasus) in Arctic, Alaska
Source: PLoS One. 2022 May 2;17(5):e0259921. doi: 10.1371/journal.pone.0259921 (PMC9060380; doi:10.1371/journal.pone.0259921)
Supplement: S2 Table — Table displaying the LA MC-ICP-MS instrument parameters used at the Alaska Stable Isotope Facility, University of Alaska Fairbanks, Fairbanks, AK, USA. (DOCX) [file pone.0259921.s002.docx]

**S2 Table.** Laser Ablation System (LA) multi collector inductively coupled plasma mass spectrometer (MC-ICP-MS) instrument parameters. Table displaying the LA MC-ICP-MS instrument parameters used at Alaska Stable Isotope Facility, University of Alaska Fairbanks, Fairbanks, AK, USA.
