# Supplementary material for: Strontium isotopes reveal diverse life history variations, migration patterns, and habitat use for Broad Whitefish (Coregonus nasus) in Arctic, Alaska
Source: PLoS One. 2022 May 2;17(5):e0259921. doi: 10.1371/journal.pone.0259921 (PMC9060380; doi:10.1371/journal.pone.0259921)
Supplement: S3 Table — Table displaying otoliths analyzed, Broad whitefish attributes, and muscle tissue stable isotope values (δ13C, δ15N, δ18O, and δD). (DOCX) [file pone.0259921.s003.docx]

**S3 Table.** Summary of Broad Whitefish (Coregonus nasus) sampled in the Colville River, AK, USA. Table displaying otoliths analyzed and attributes. All stable isotope values are expressed in er mil (‰) relative to international standards. Spawning condition was assessed based on gonadsomatic index (GSI) for females or the presence of nuptial tubercles for males.

**S3 Table (Continued)**

Legend: spawning condition; Prespawn = females with gonadosomatic index values ≥ 3 or males with nuptial tubercles present, mature = females with gonadosomatic index value < 3, NA = male individuals without nuptial tubercles present. Life history type; type 1 = early marine anadromous, type 2 = late freshwater anadromous, type 3 = late transitional anadromous, type 4 = semi-anadromous, type 5 = freshwater estuarine nonanadromous, and type 6 = nonanadromous.
