# Supplementary material for: Strontium isotopes reveal diverse life history variations, migration patterns, and habitat use for Broad Whitefish (Coregonus nasus) in Arctic, Alaska
Source: PLoS One. 2022 May 2;17(5):e0259921. doi: 10.1371/journal.pone.0259921 (PMC9060380; doi:10.1371/journal.pone.0259921)
Supplement: S1 File — Plots showing 88Sr concentration and 87Sr/86Sr for all Broad Whitefish (Coregonus nasus) caught within the Colville River watershed, Alaska, USA. We cropped strontium data for otoliths analyzed (n = 61) at the otolith core and edge. (PDF) [file pone.0259921.s004.pdf]

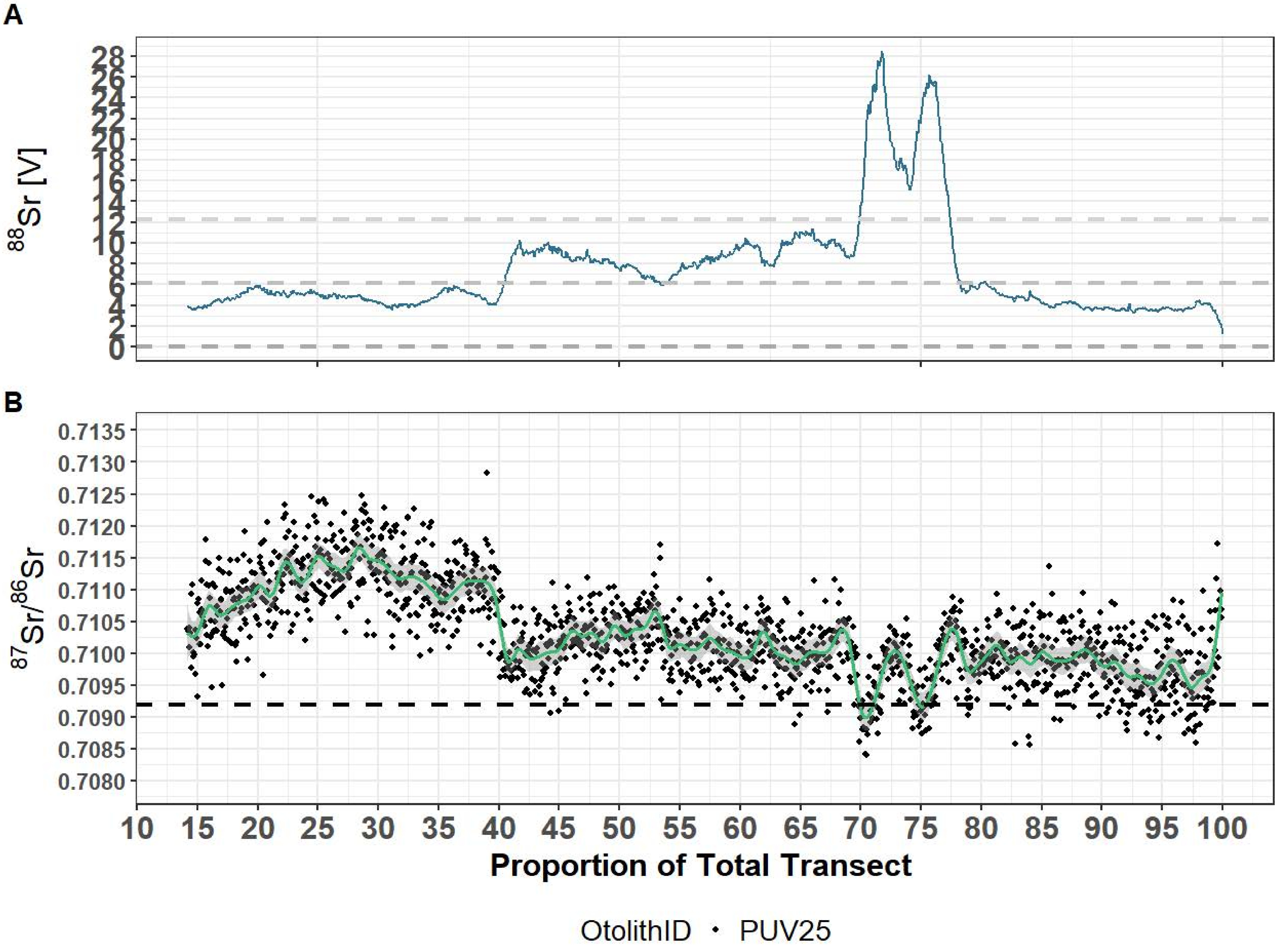

A
^{88}Sr [V]
0
2
4
6
8
10
12
14
16
18
20
22
24
26
28
B
$^{87}Sr/^{86}Sr$
0.7080
0.7085
0.7090
0.7095
0.7100
0.7105
0.7110
0.7115
0.7120
0.7125
0.7130
0.7135
10
15
20
25
30
35
40
45
50
55
60
65
70
75
80
85
90
95
100
Proportion of Total Transect
OtolithID • PUV25

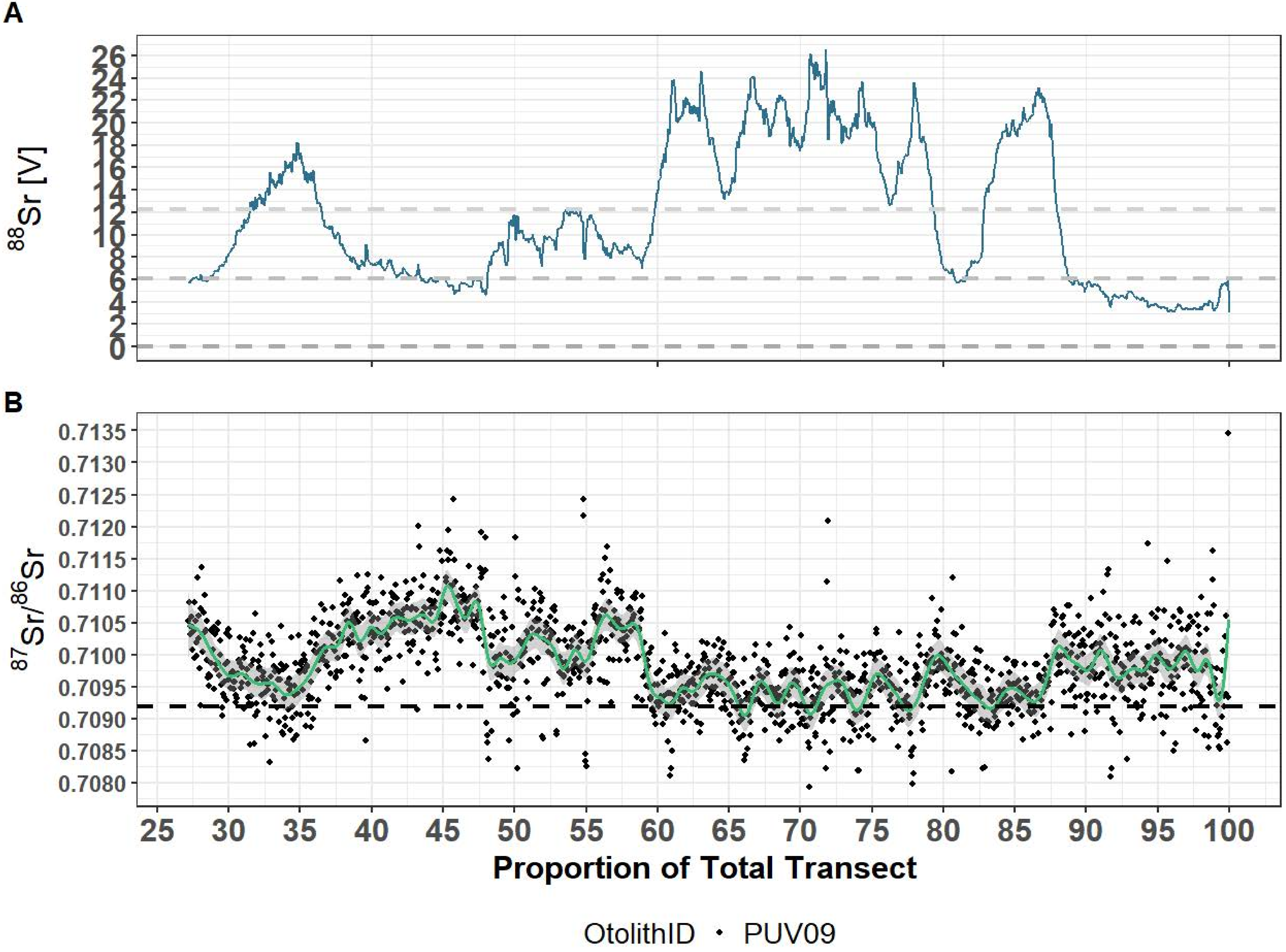

A
B
^{88}Sr [V]
^{87}Sr/^{86}Sr
Proportion of Total Transect
OtolithID • PUV09

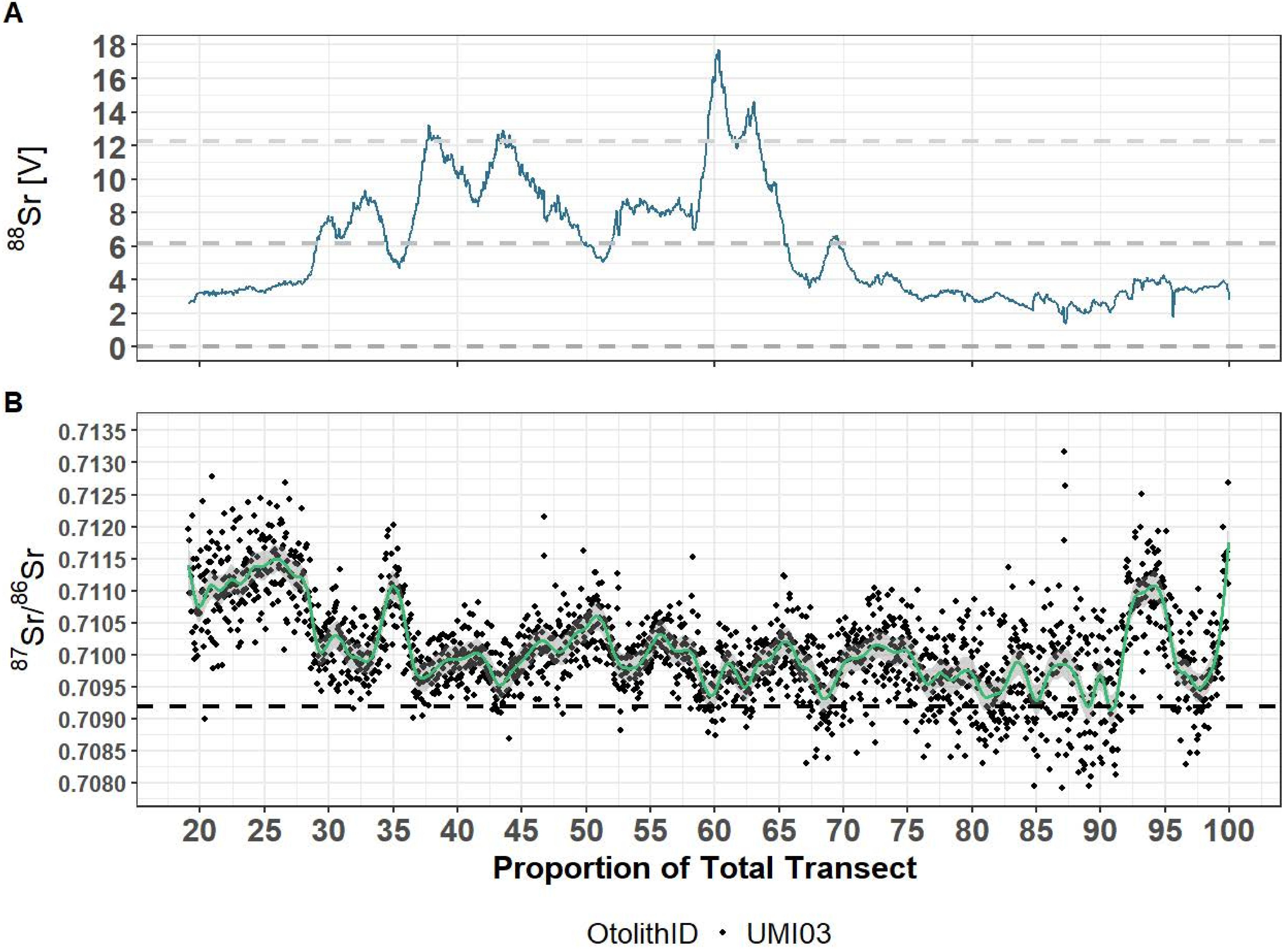

A
B
^{88}Sr [V]
^{87}Sr/^{86}Sr
Proportion of Total Transect
OtolithID
UMI03

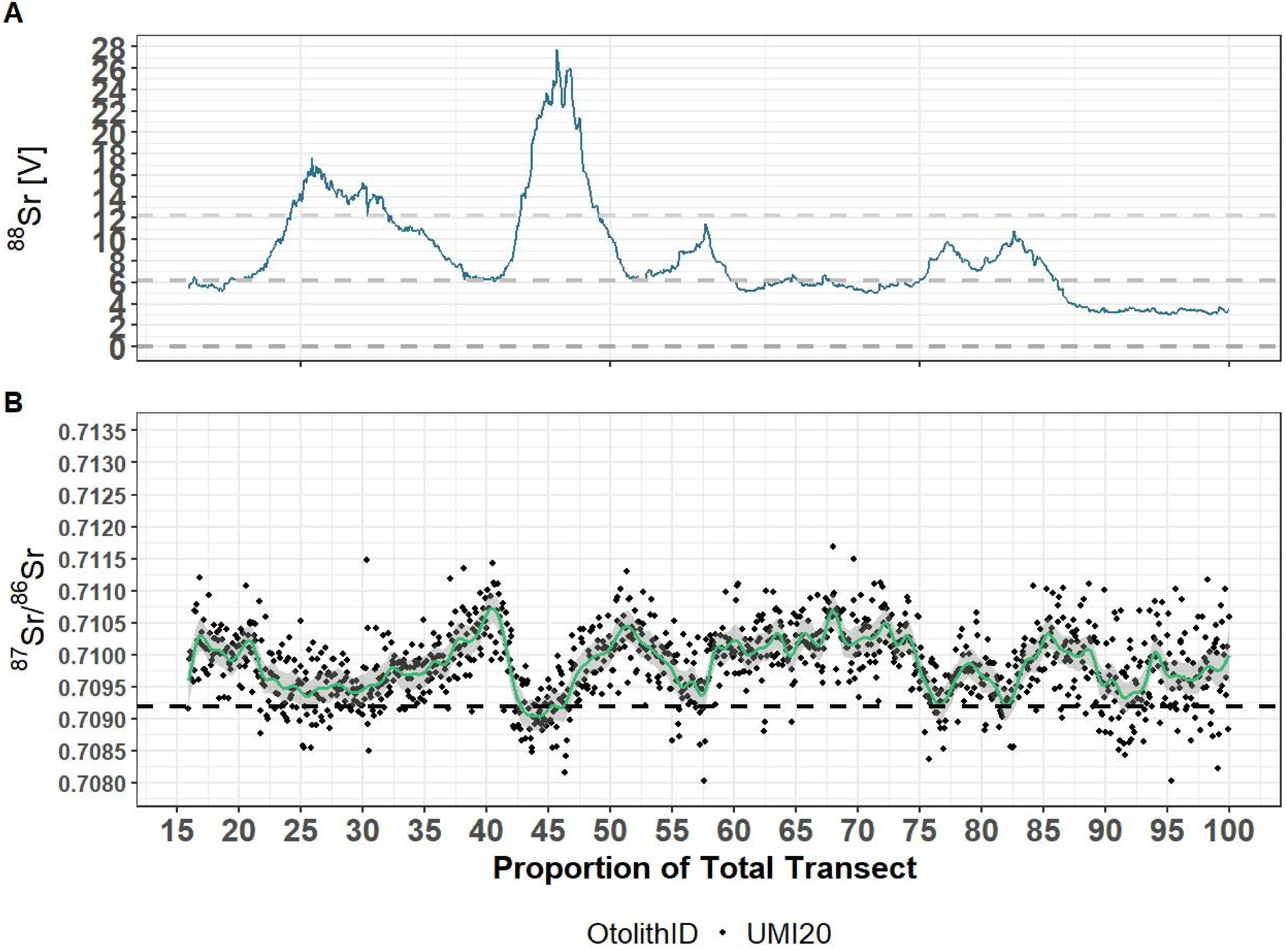
A
^{88}Sr [V]
0
2
4
6
8
10
12
14
16
18
20
22
24
26
28
B
$^{87}Sr/^{86}Sr$
0.7080
0.7085
0.7090
0.7095
0.7100
0.7105
0.7110
0.7115
0.7120
0.7125
0.7130
0.7135
15
20
25
30
35
40
45
50
55
60
65
70
75
80
85
90
95
100
Proportion of Total Transect
OtolithID
UMI20

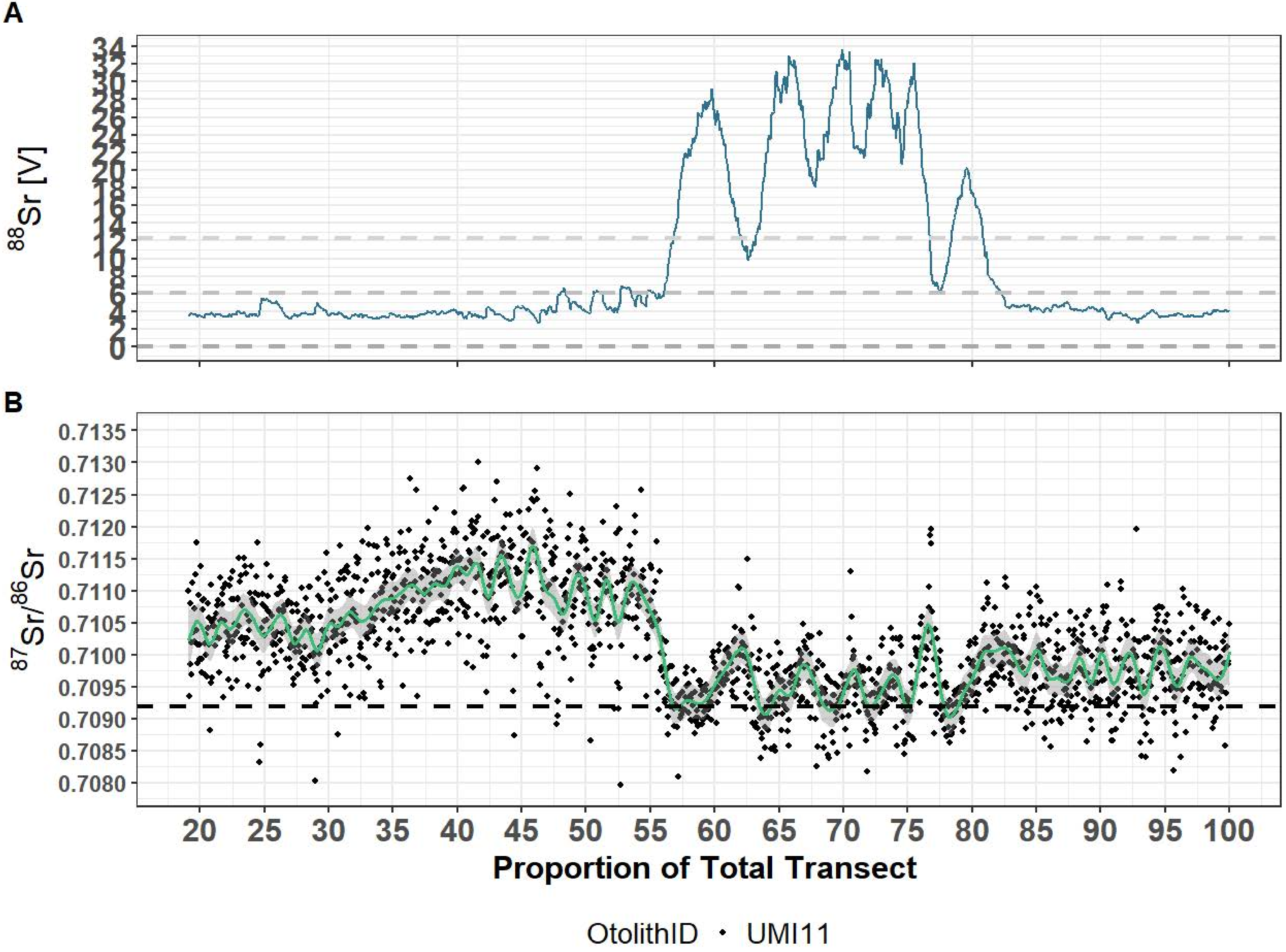
A
^{88}Sr [V]
B
$^{87}Sr/^{86}Sr$
0.7135
0.7130
0.7125
0.7120
0.7115
0.7110
0.7105
0.7100
0.7095
0.7090
0.7085
0.7080
20
25
30
35
40
45
50
55
60
65
70
75
80
85
90
95
100
Proportion of Total Transect
OtolithID
UMI11

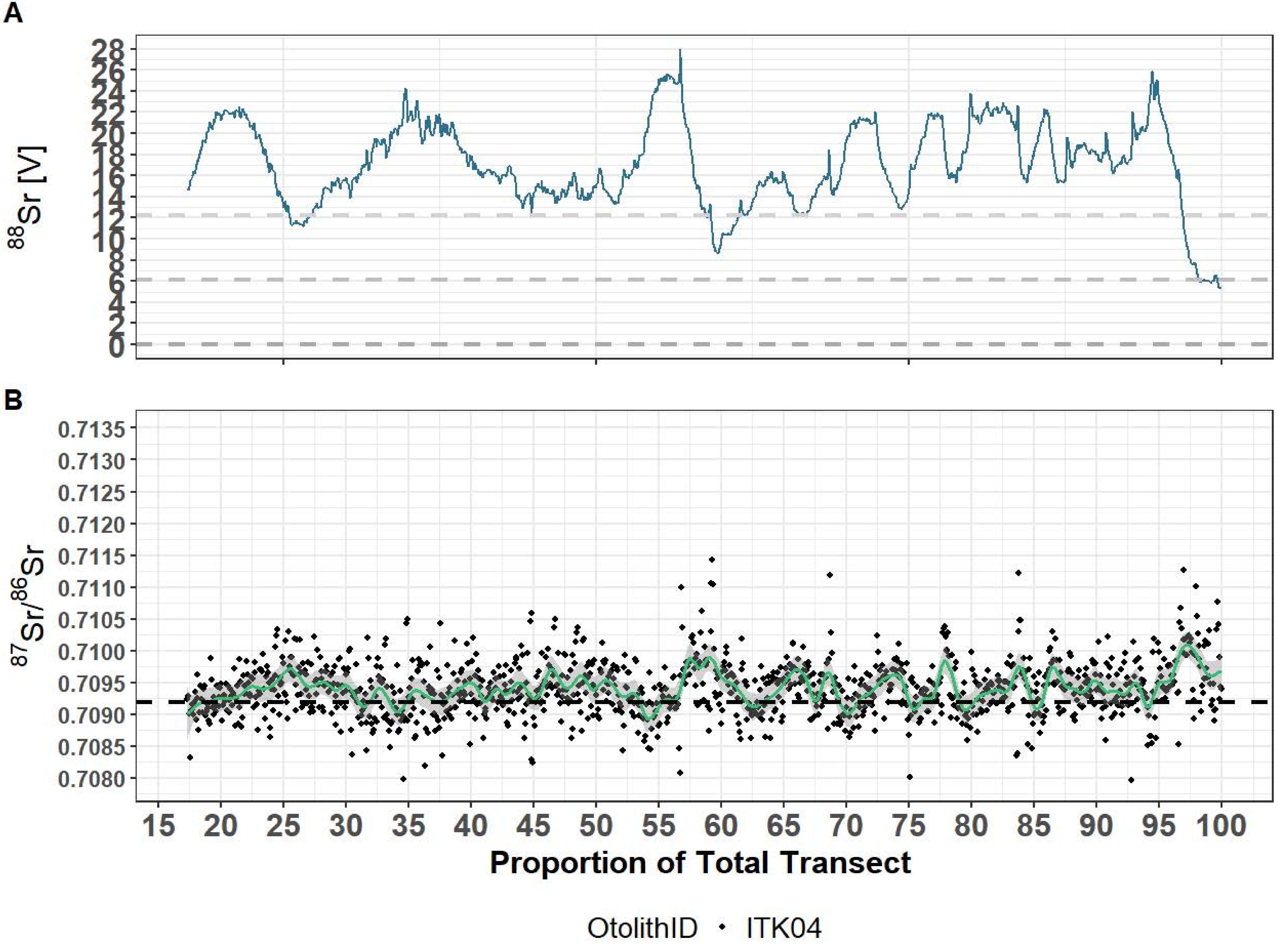

A
B
^{88}Sr [V]
0
2
4
6
8
10
12
14
16
18
20
22
24
26
28
^{87}Sr/^{86}Sr
0.7080
0.7085
0.7090
0.7095
0.7100
0.7105
0.7110
0.7115
0.7120
0.7125
0.7130
0.7135
15 20 25 30 35 40 45 50 55 60 65 70 75 80 85 90 95 100
Proportion of Total Transect
OtolithID • ITK04

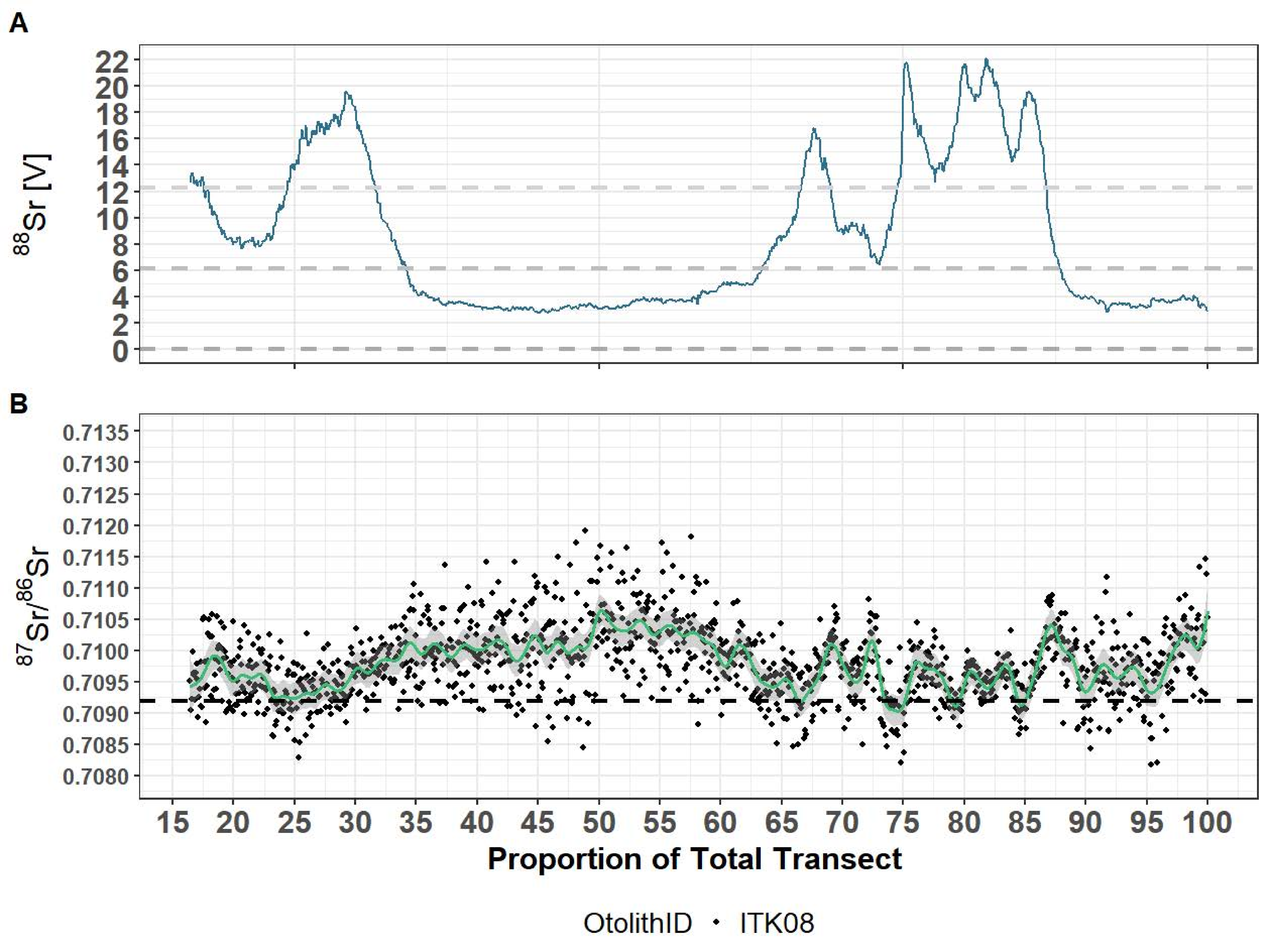

A
22
20
18
16
14
12
10
8
6
4
2
0
88Sr [V]
B
0.7135
0.7130
0.7125
0.7120
0.7115
0.7110
0.7105
0.7100
0.7095
0.7090
0.7085
0.7080
87Sr/86Sr
15 20 25 30 35 40 45 50 55 60 65 70 75 80 85 90 95 100
Proportion of Total Transect
OtolithID • ITK08

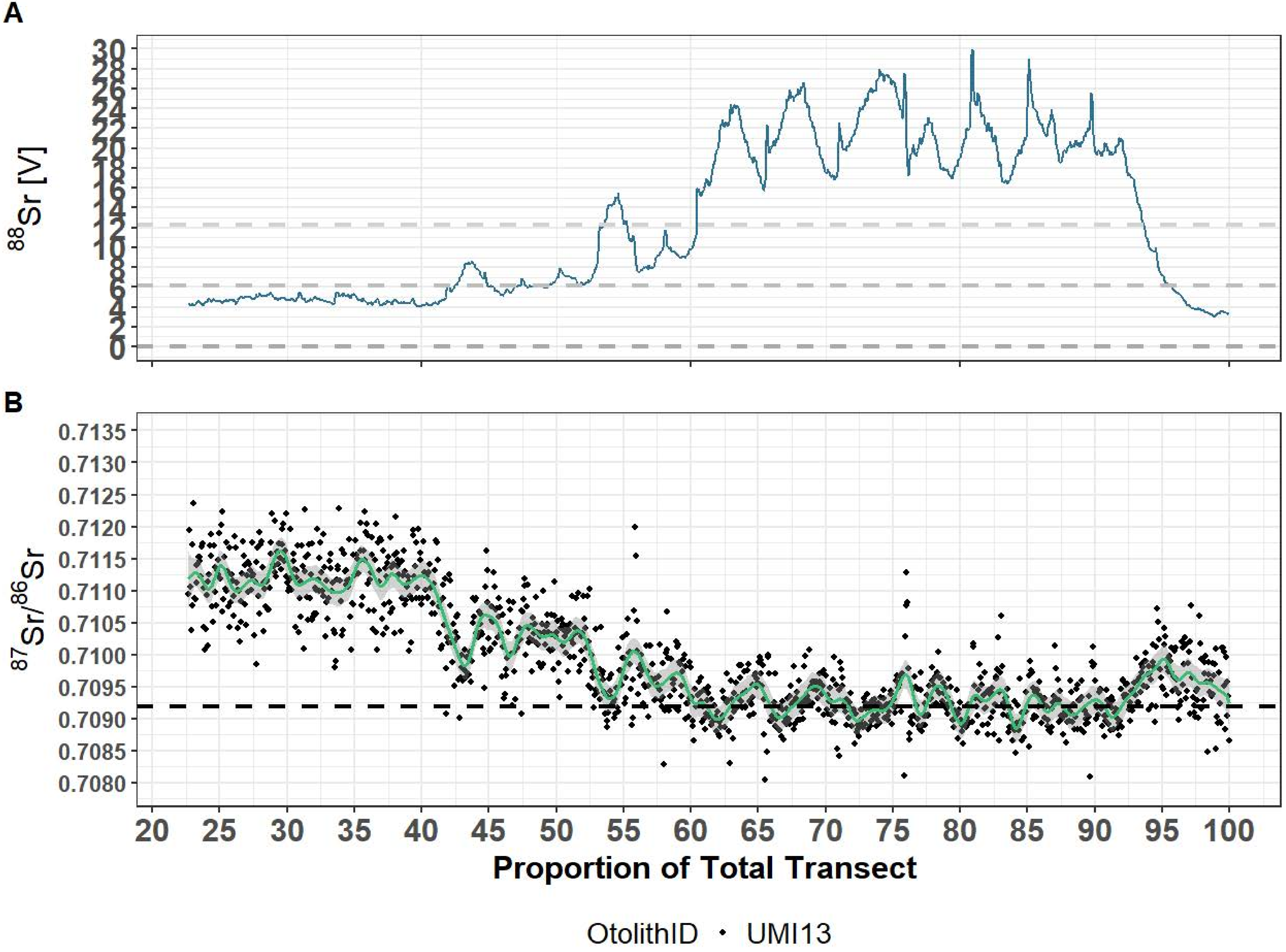

A
^{88}Sr [V]
30
28
26
24
22
20
18
16
14
12
10
8
6
4
2
0
B
^{87}Sr/^{86}Sr
0.7135
0.7130
0.7125
0.7120
0.7115
0.7110
0.7105
0.7100
0.7095
0.7090
0.7085
0.7080
20
25
30
35
40
45
50
55
60
65
70
75
80
85
90
95
100
Proportion of Total Transect
OtolithID
UMI13

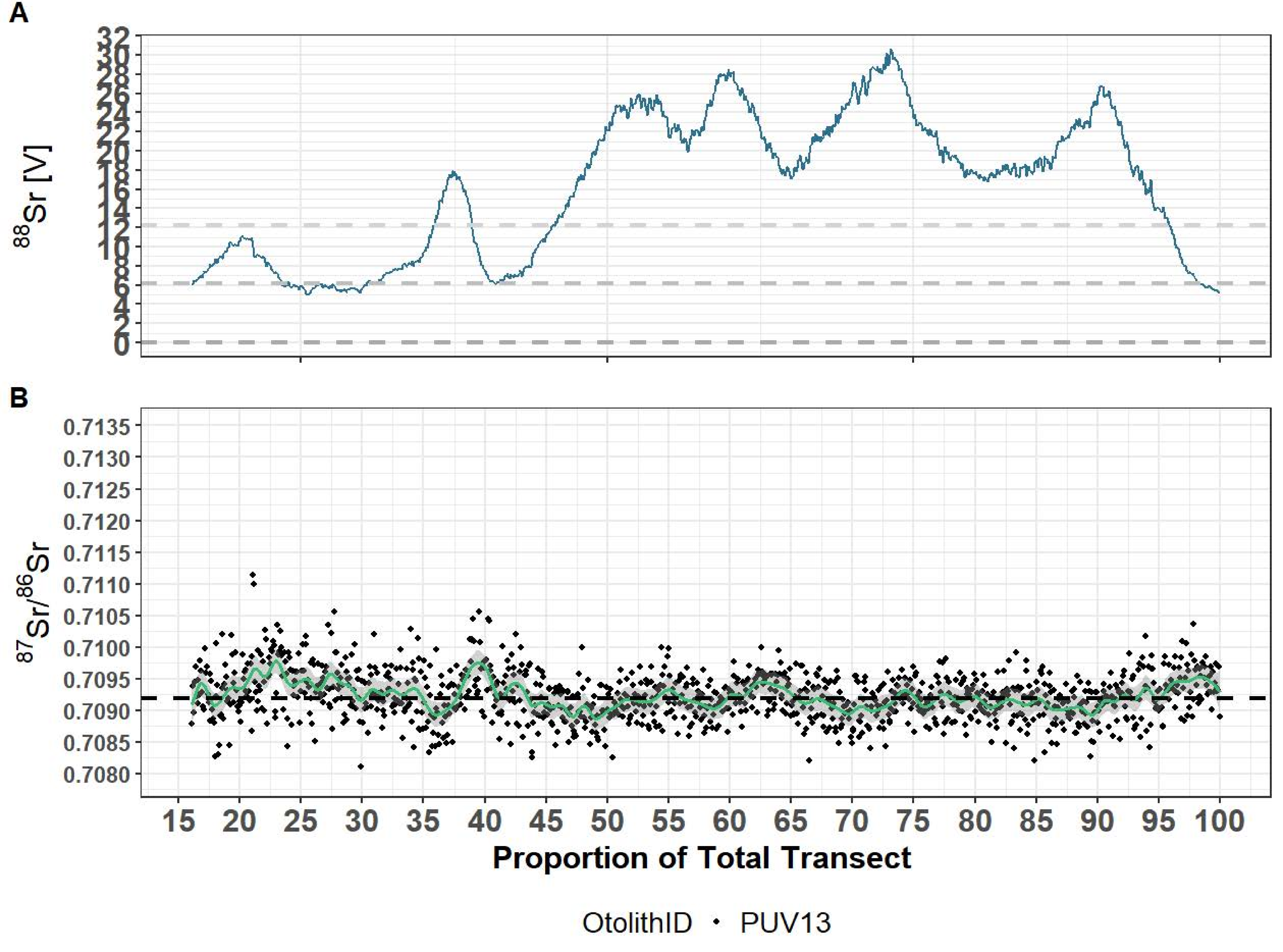

A
B
^{88}Sr [V]
0
2
4
6
8
10
12
14
16
18
20
22
24
26
28
30
32
^{87}Sr/^{86}Sr
0.7080
0.7085
0.7090
0.7095
0.7100
0.7105
0.7110
0.7115
0.7120
0.7125
0.7130
0.7135
15
20
25
30
35
40
45
50
55
60
65
70
75
80
85
90
95
100
Proportion of Total Transect
OtolithID • PUV13

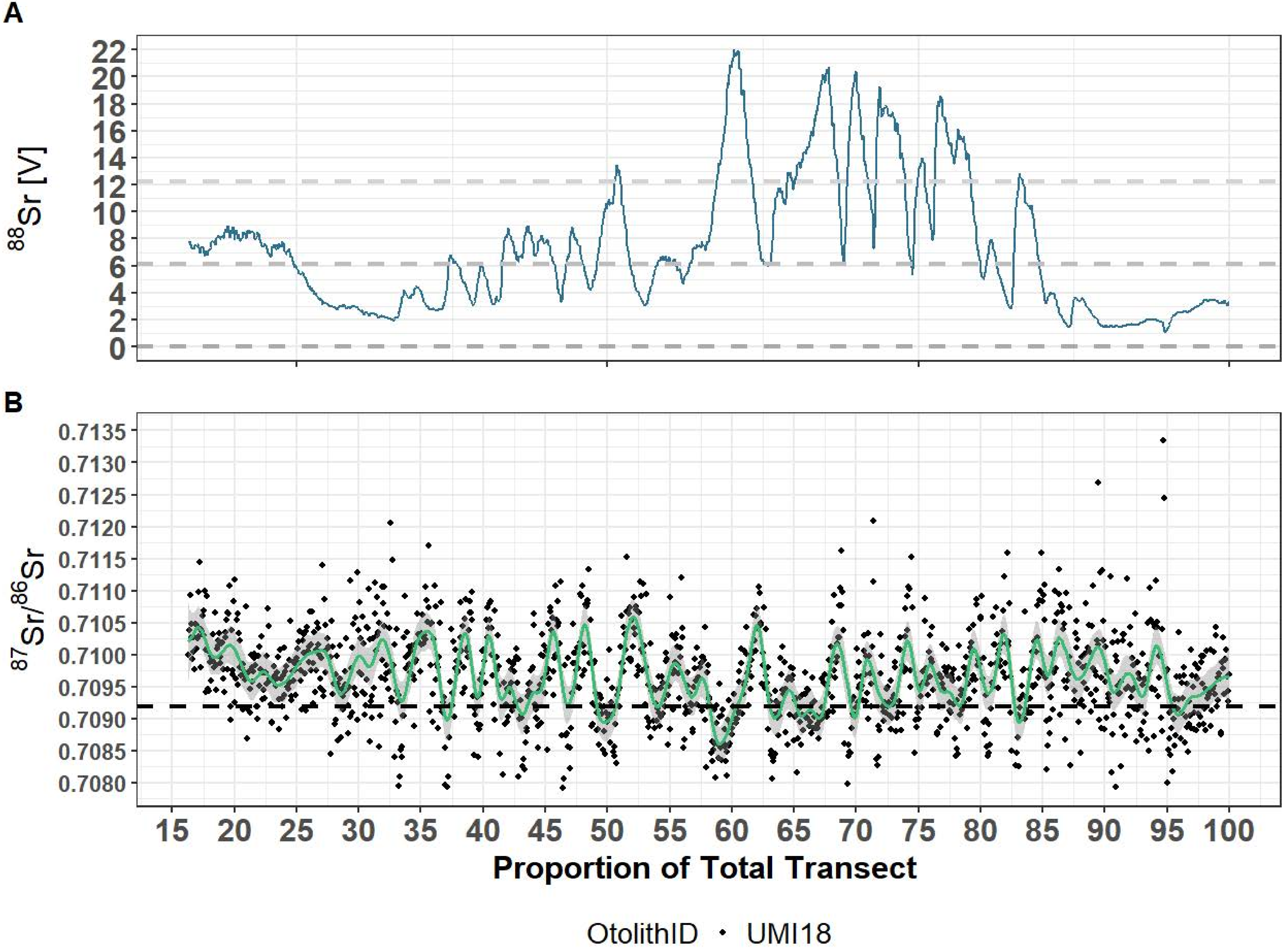
A
^{88}Sr [V]
0
2
4
6
8
10
12
14
16
18
20
22
B
^{87}Sr/^{86}Sr
0.7080
0.7085
0.7090
0.7095
0.7100
0.7105
0.7110
0.7115
0.7120
0.7125
0.7130
0.7135
15 20 25 30 35 40 45 50 55 60 65 70 75 80 85 90 95 100
Proportion of Total Transect
OtolithID
UMI18

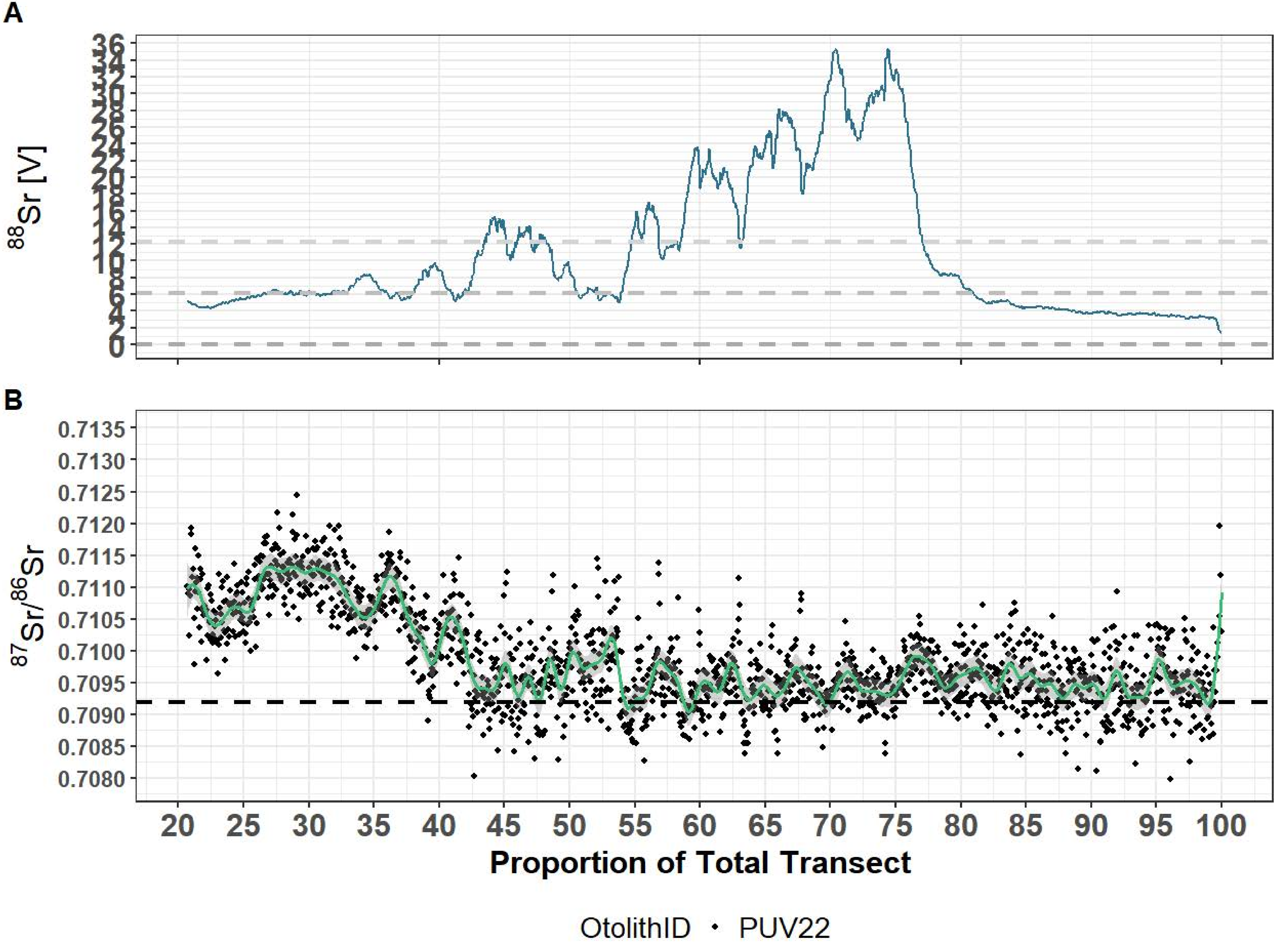

A
88Sr [V]
36
34
32
30
28
26
24
22
20
18
16
14
12
10
8
6
4
2
0
B
87Sr/86Sr
0.7135
0.7130
0.7125
0.7120
0.7115
0.7110
0.7105
0.7100
0.7095
0.7090
0.7085
0.7080
20 25 30 35 40 45 50 55 60 65 70 75 80 85 90 95 100
Proportion of Total Transect
OtolithID • PUV22

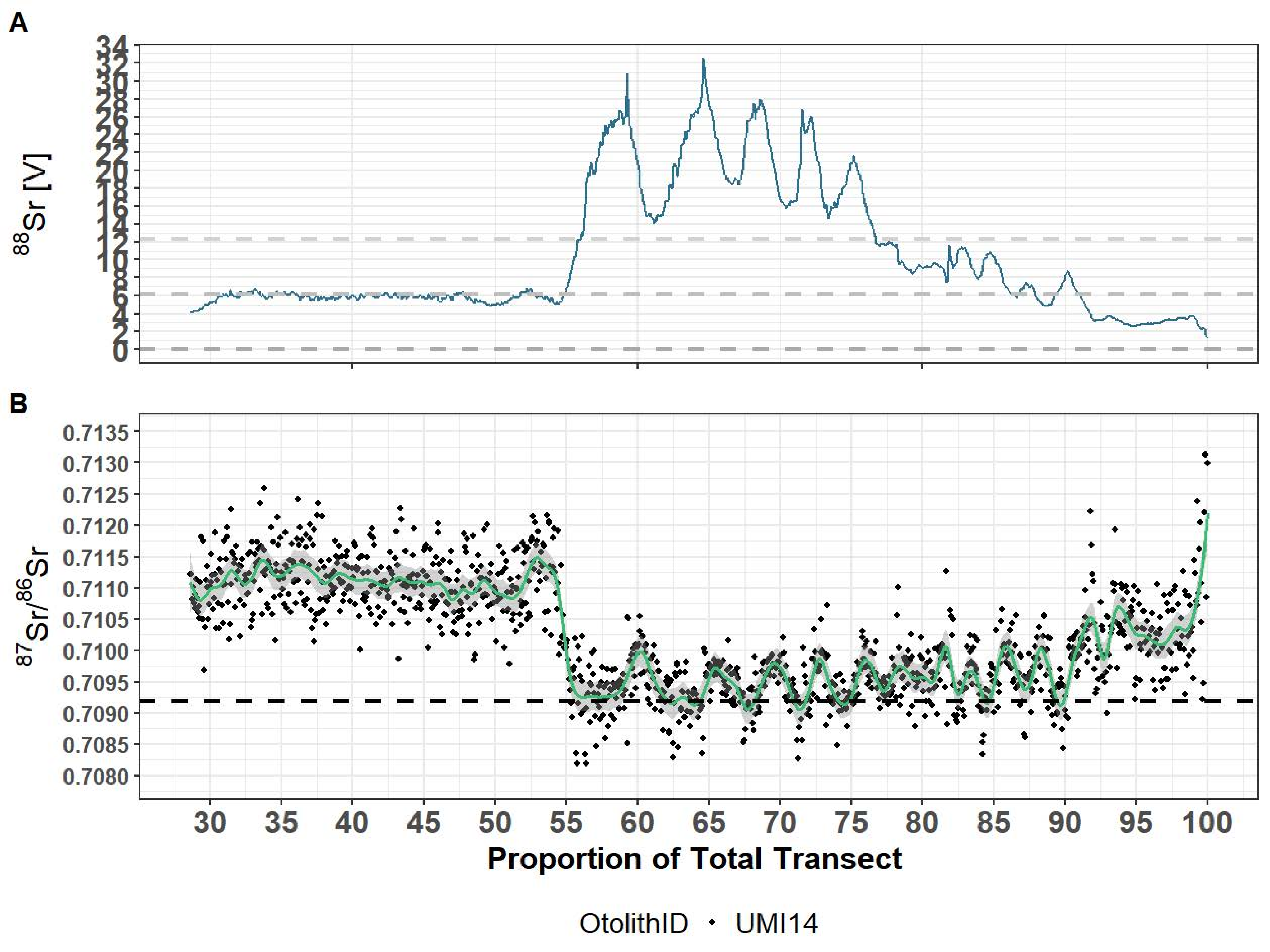

A
^{88}Sr [V]
34
32
30
28
26
24
22
20
18
16
14
12
10
8
6
4
2
0
B
^{87}Sr/^{86}Sr
0.7135
0.7130
0.7125
0.7120
0.7115
0.7110
0.7105
0.7100
0.7095
0.7090
0.7085
0.7080
30 35 40 45 50 55 60 65 70 75 80 85 90 95 100
Proportion of Total Transect
OtolithID • UMI14

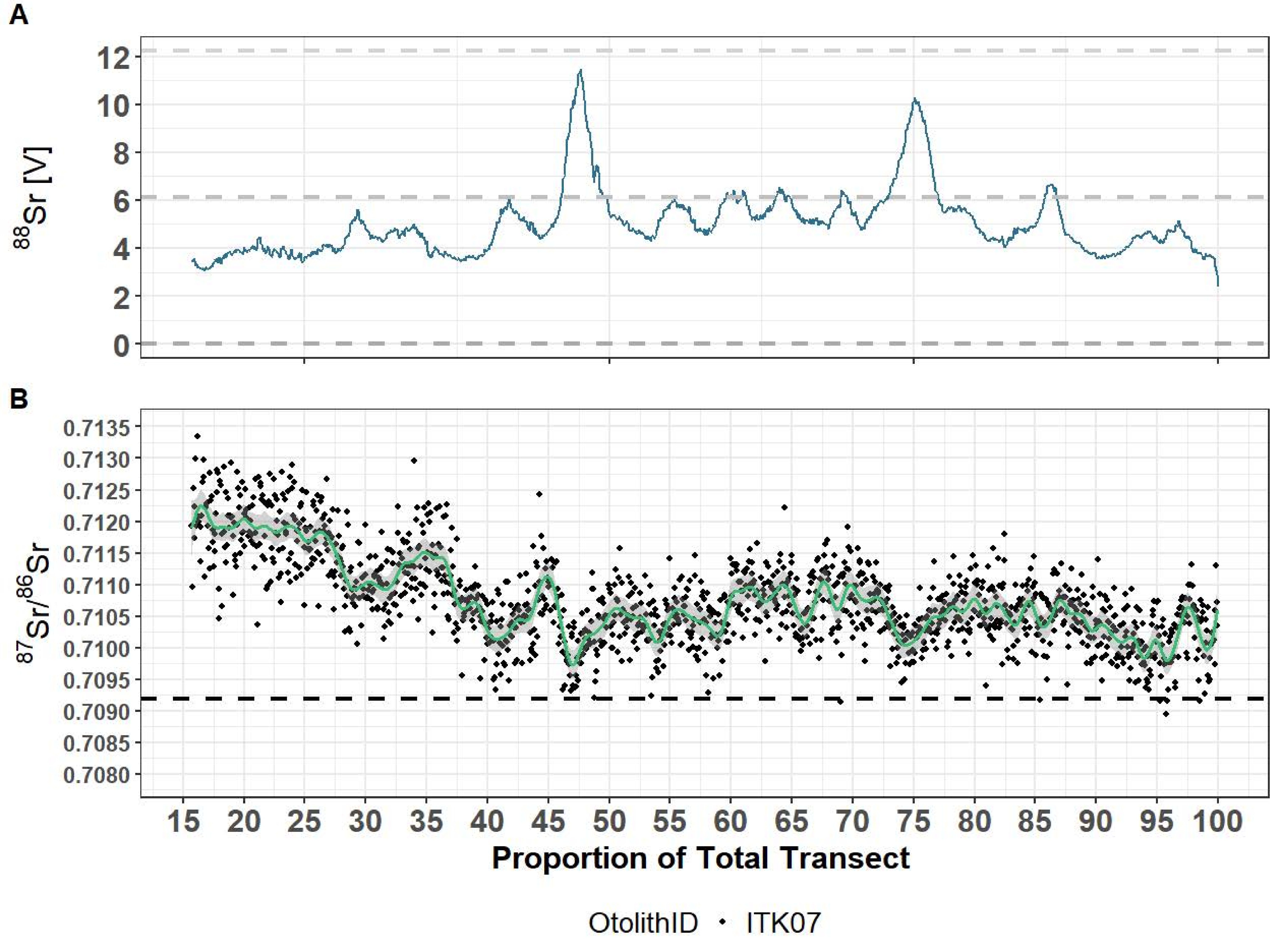
A
B
^{88}Sr [V]
0
2
4
6
8
10
12
^{87}Sr/^{86}Sr
0.7080
0.7085
0.7090
0.7095
0.7100
0.7105
0.7110
0.7115
0.7120
0.7125
0.7130
0.7135
15 20 25 30 35 40 45 50 55 60 65 70 75 80 85 90 95 100
Proportion of Total Transect
OtolithID • ITK07

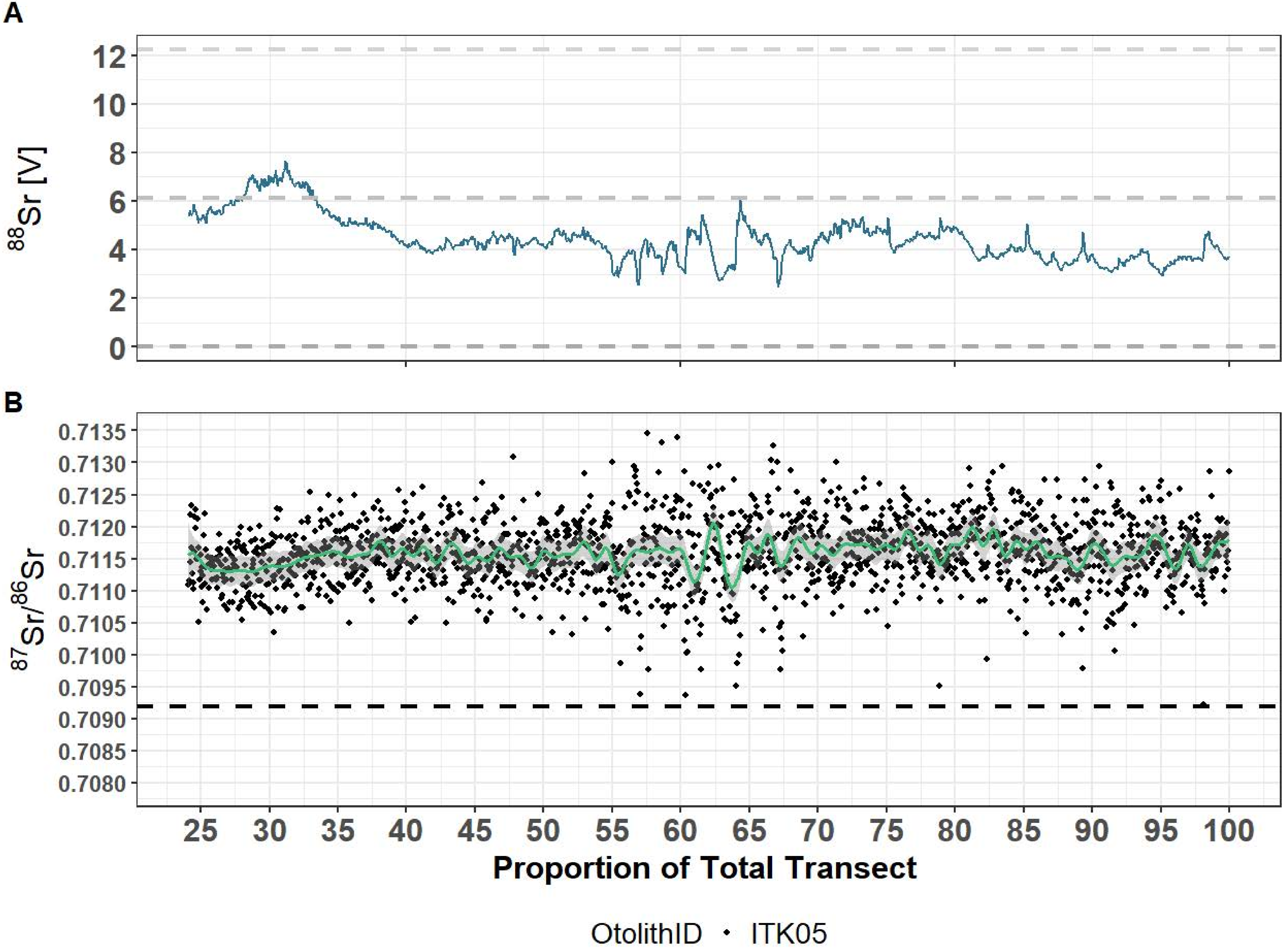

A
12
10
8
6
4
2
0
^{88}Sr [V]
B
0.7135
0.7130
0.7125
0.7120
0.7115
0.7110
0.7105
0.7100
0.7095
0.7090
0.7085
0.7080
^{87}Sr/^{86}Sr
25 30 35 40 45 50 55 60 65 70 75 80 85 90 95 100
Proportion of Total Transect
OtolithID • ITK05

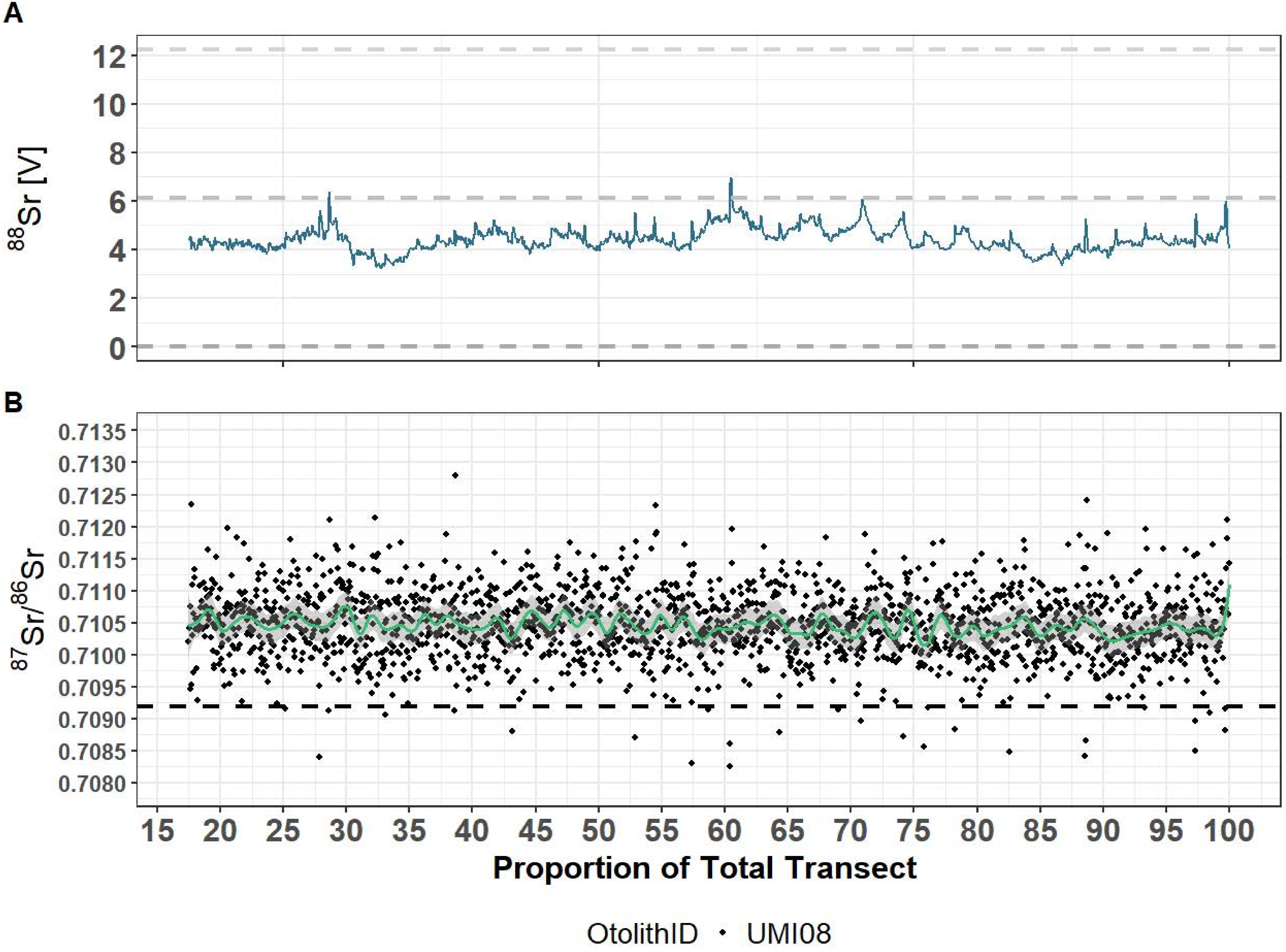
A
B
88Sr [V]
0
2
4
6
8
10
12
87Sr/86Sr
0.7080
0.7085
0.7090
0.7095
0.7100
0.7105
0.7110
0.7115
0.7120
0.7125
0.7130
0.7135
15
20
25
30
35
40
45
50
55
60
65
70
75
80
85
90
95
100
Proportion of Total Transect
OtolithID
UMI08

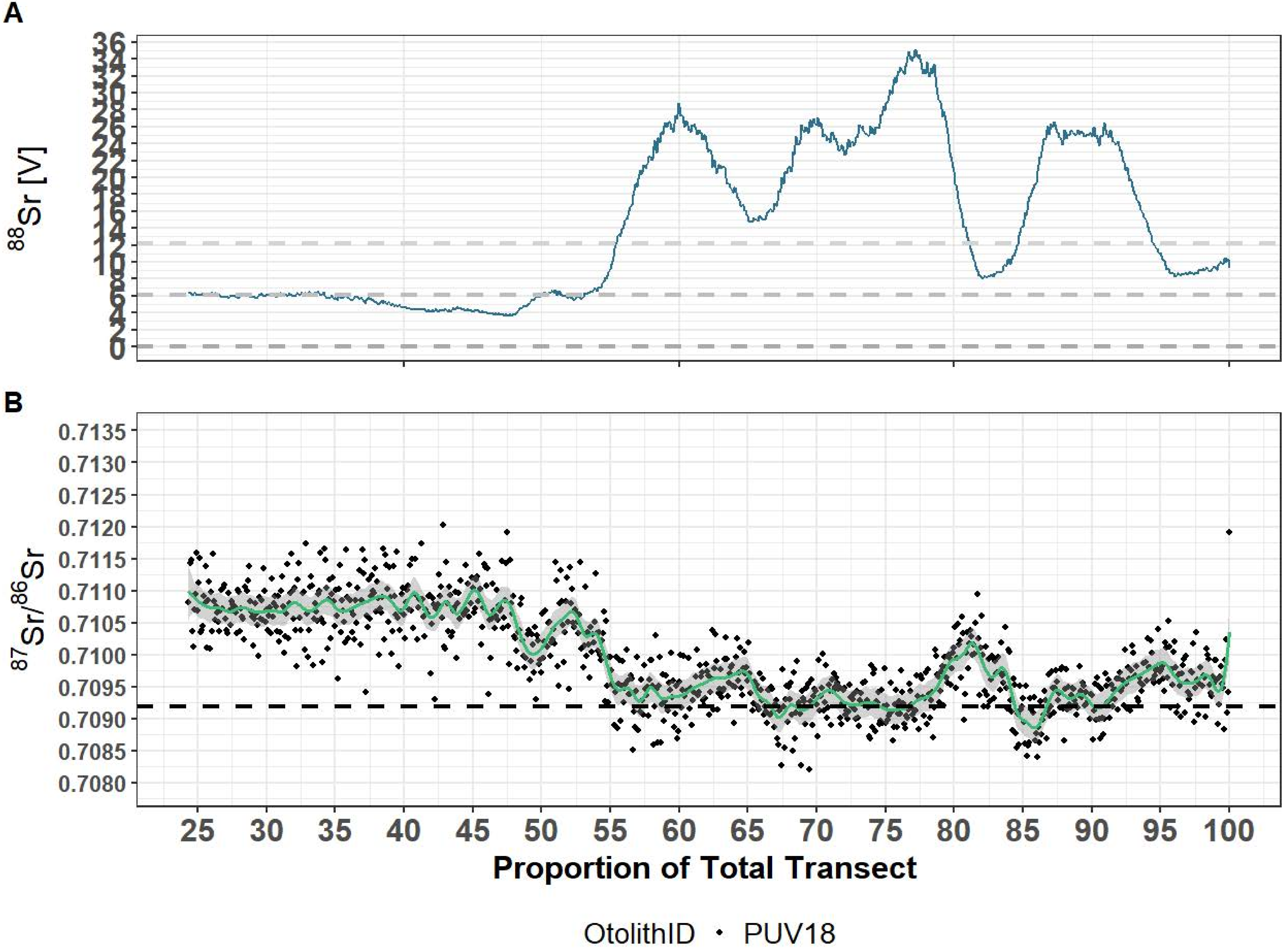
A
^{88}Sr [V]
0
2
4
6
8
10
12
14
16
18
20
22
24
26
28
30
32
34
36
B
$^{87}Sr/^{86}Sr$
0.7080
0.7085
0.7090
0.7095
0.7100
0.7105
0.7110
0.7115
0.7120
0.7125
0.7130
0.7135
25 30 35 40 45 50 55 60 65 70 75 80 85 90 95 100
Proportion of Total Transect
OtolithID • PUV18

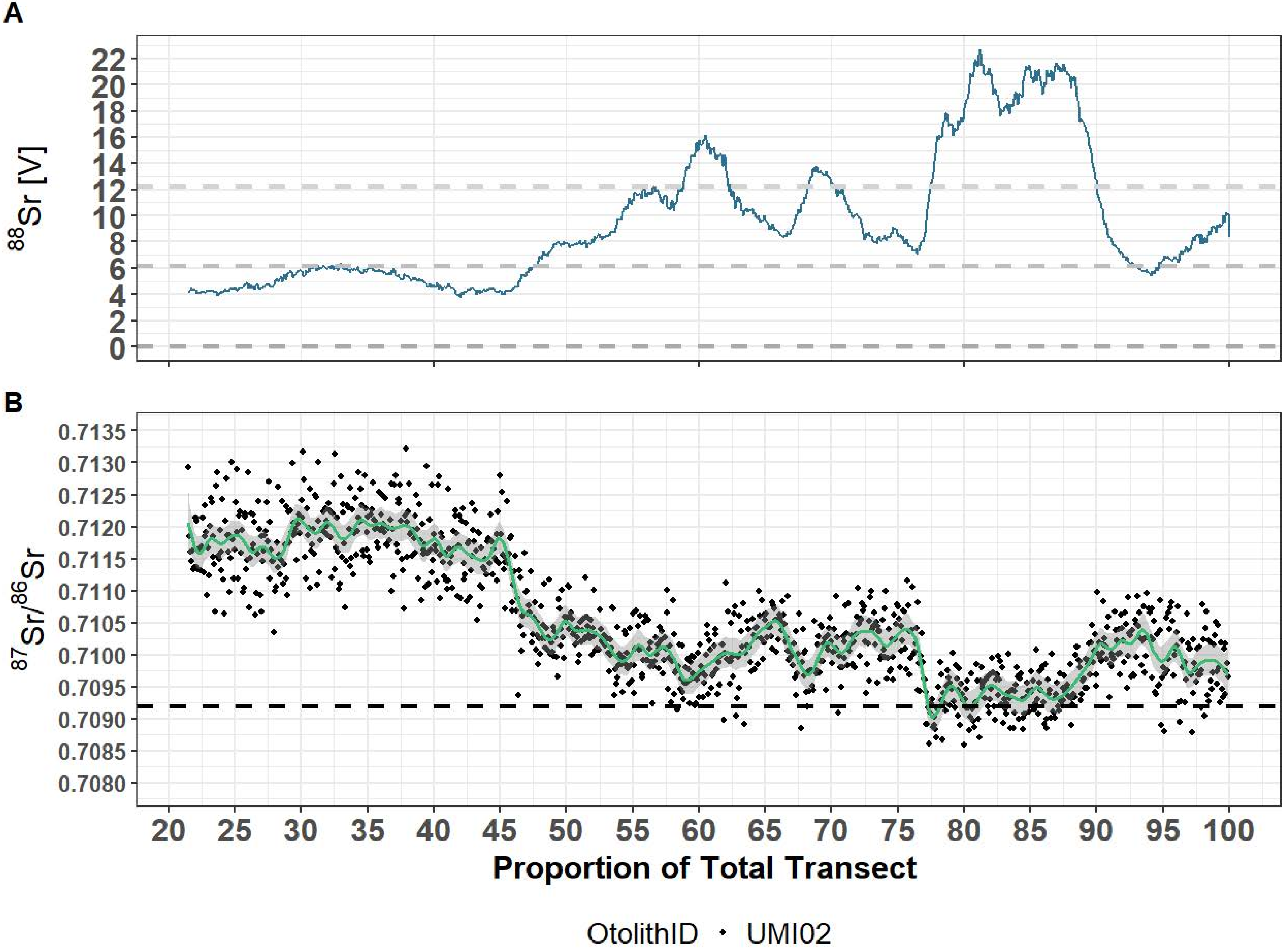
A
B
^{88}Sr [V]
0
2
4
6
8
10
12
14
16
18
20
22
$^{87}Sr/^{86}Sr$
0.7080
0.7085
0.7090
0.7095
0.7100
0.7105
0.7110
0.7115
0.7120
0.7125
0.7130
0.7135
20 25 30 35 40 45 50 55 60 65 70 75 80 85 90 95 100
Proportion of Total Transect
OtolithID • UMI02

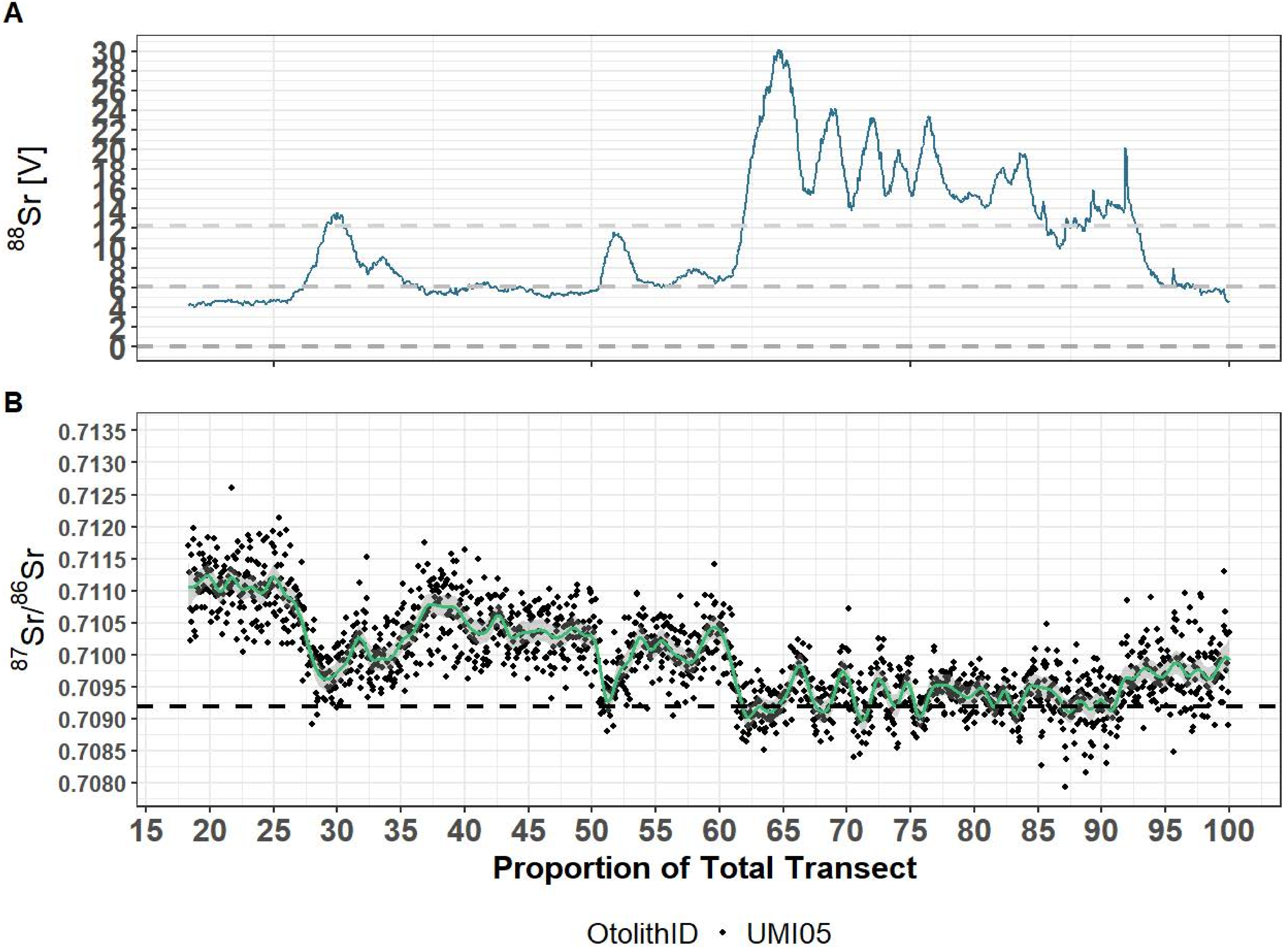
A
^{88}Sr [V]
0
2
4
6
8
10
12
14
16
18
20
22
24
26
28
30
B
^{87}Sr/^{86}Sr
0.7080
0.7085
0.7090
0.7095
0.7100
0.7105
0.7110
0.7115
0.7120
0.7125
0.7130
0.7135
15
20
25
30
35
40
45
50
55
60
65
70
75
80
85
90
95
100
Proportion of Total Transect
OtolithID • UMI05

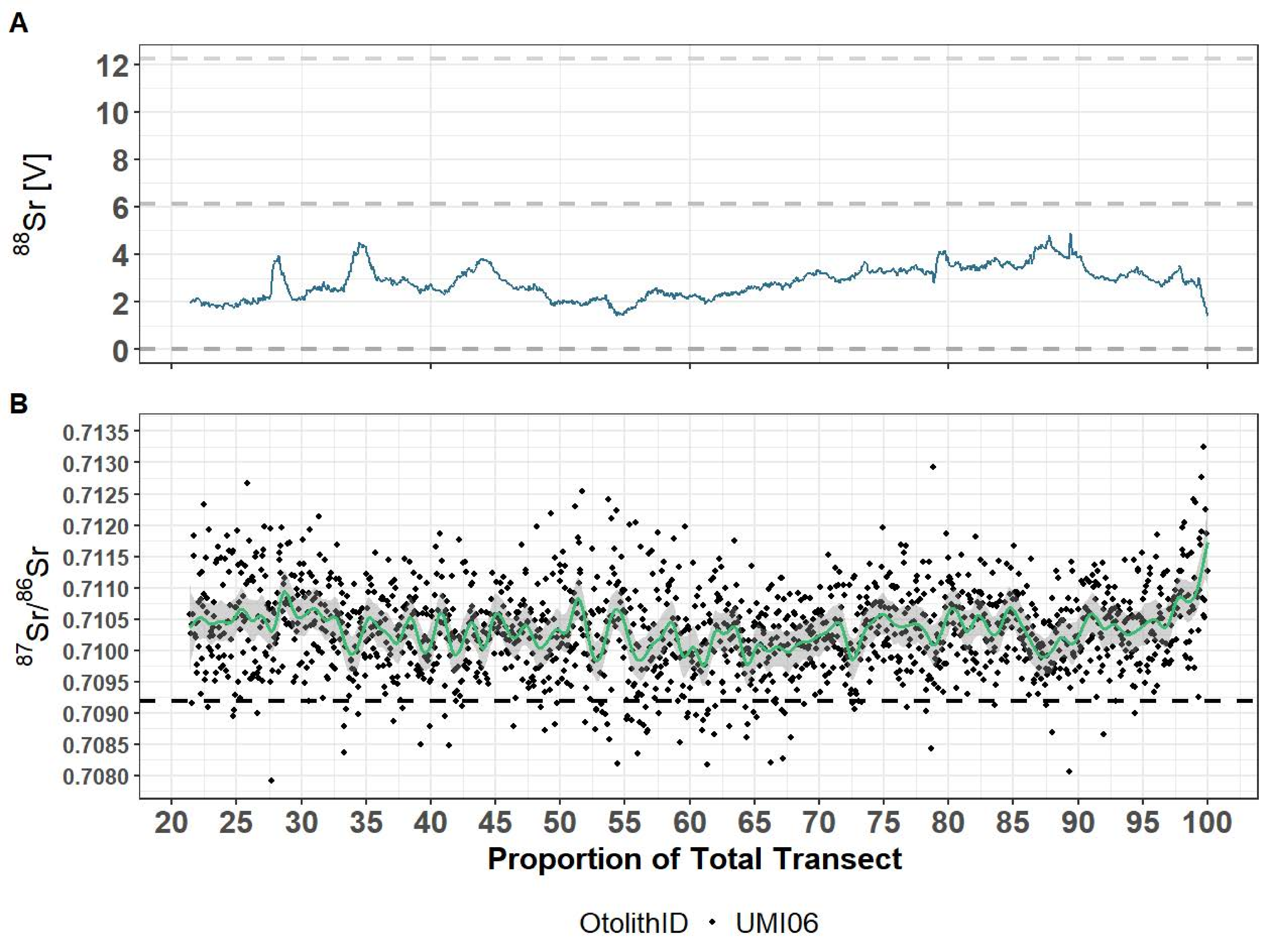

A
B
^{88}Sr [V]
^{87}Sr/^{86}Sr
Proportion of Total Transect
OtolithID • UMI06

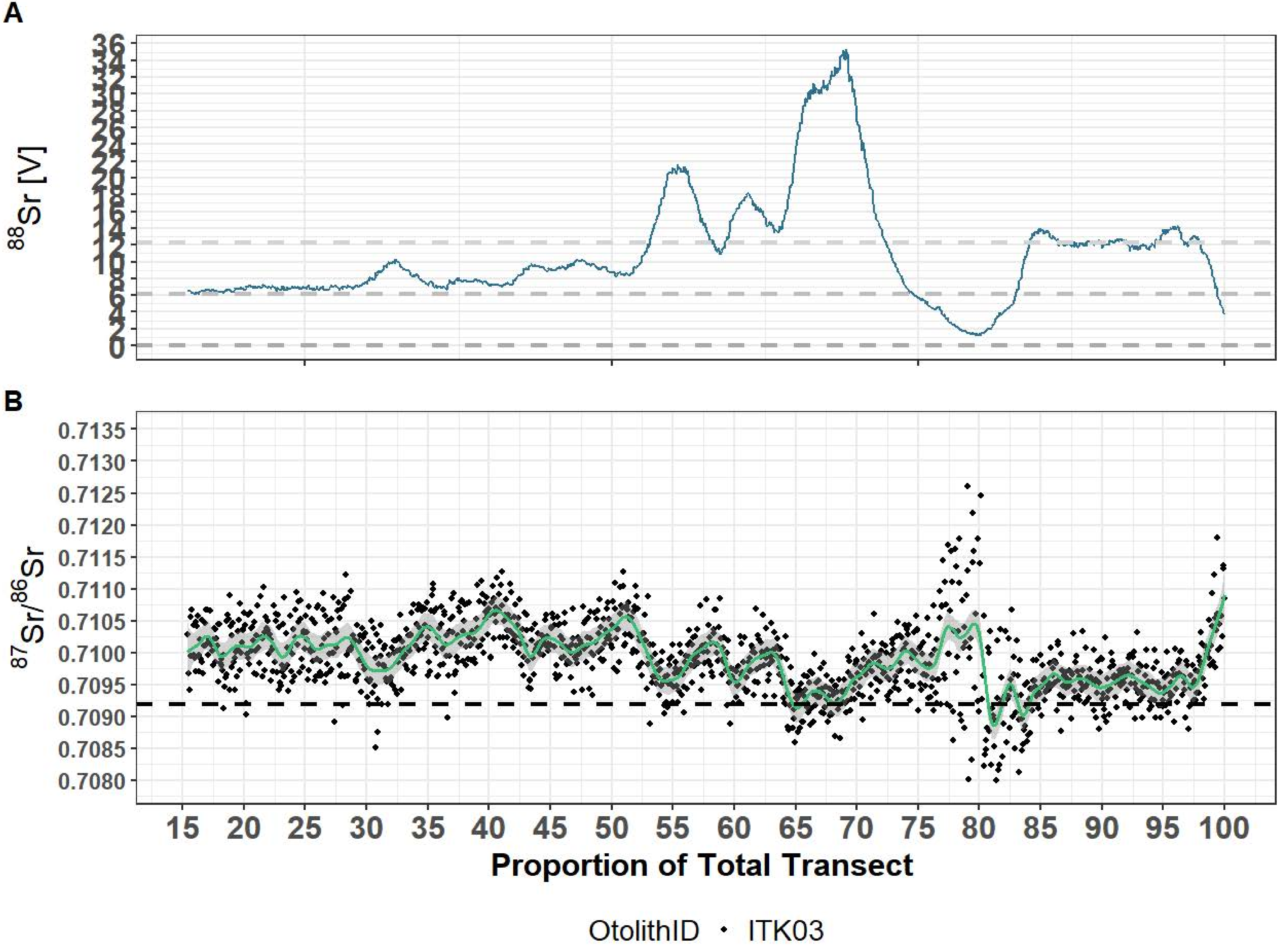

A
B
^{88}Sr [V]
0 2 4 6 8 10 12 14 16 18 20 22 24 26 28 30 32 34 36
$^{87}Sr/^{86}Sr$
0.7080 0.7085 0.7090 0.7095 0.7100 0.7105 0.7110 0.7115 0.7120 0.7125 0.7130 0.7135
15 20 25 30 35 40 45 50 55 60 65 70 75 80 85 90 95 100
Proportion of Total Transect
OtolithID ITK03

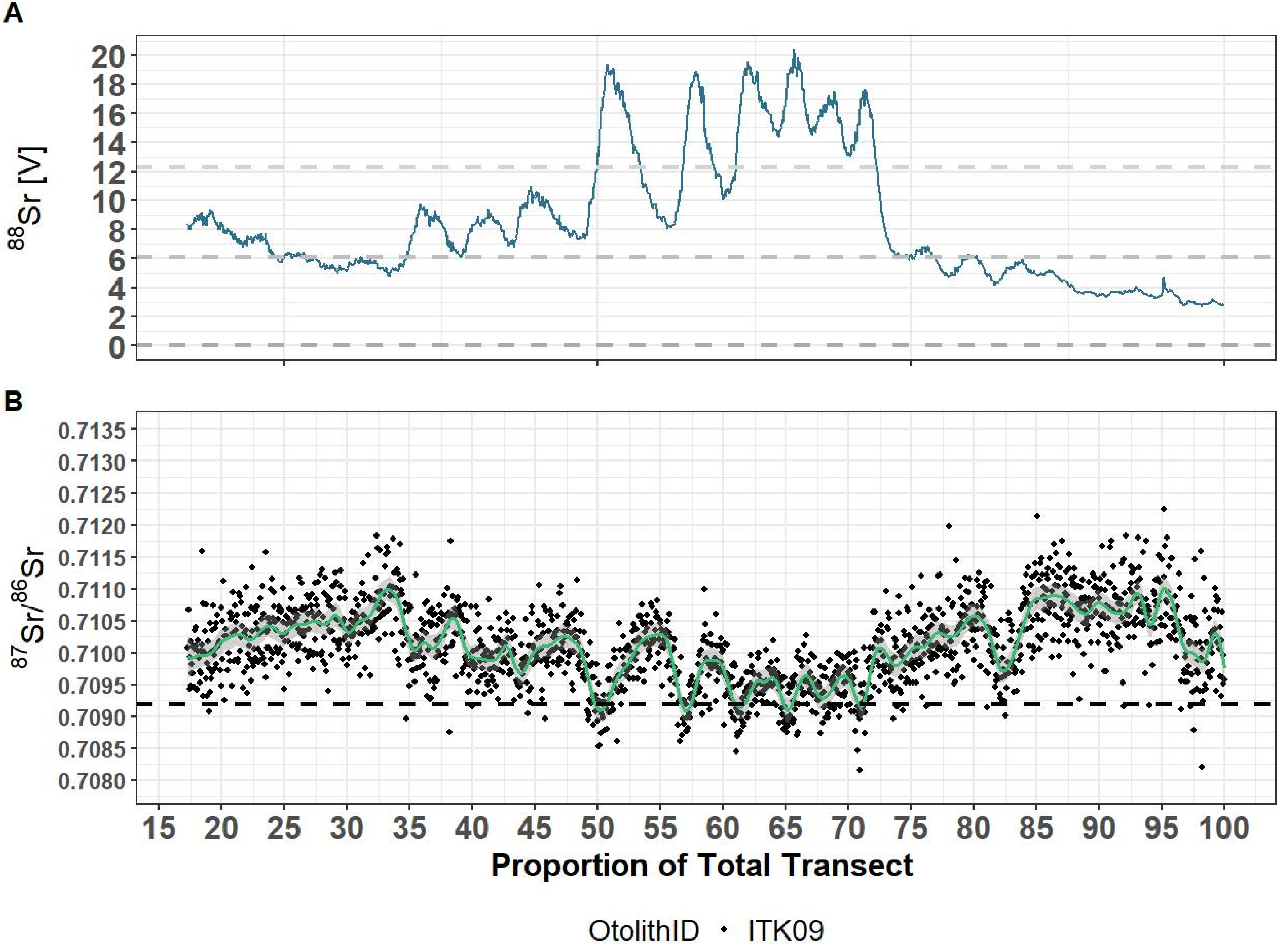

A
B
20
18
16
14
12
10
8
6
4
2
0
^{88}Sr [V]
0.7135
0.7130
0.7125
0.7120
0.7115
0.7110
0.7105
0.7100
0.7095
0.7090
0.7085
0.7080
^{87}Sr/^{86}Sr
15 20 25 30 35 40 45 50 55 60 65 70 75 80 85 90 95 100
Proportion of Total Transect
OtolithID
ITK09

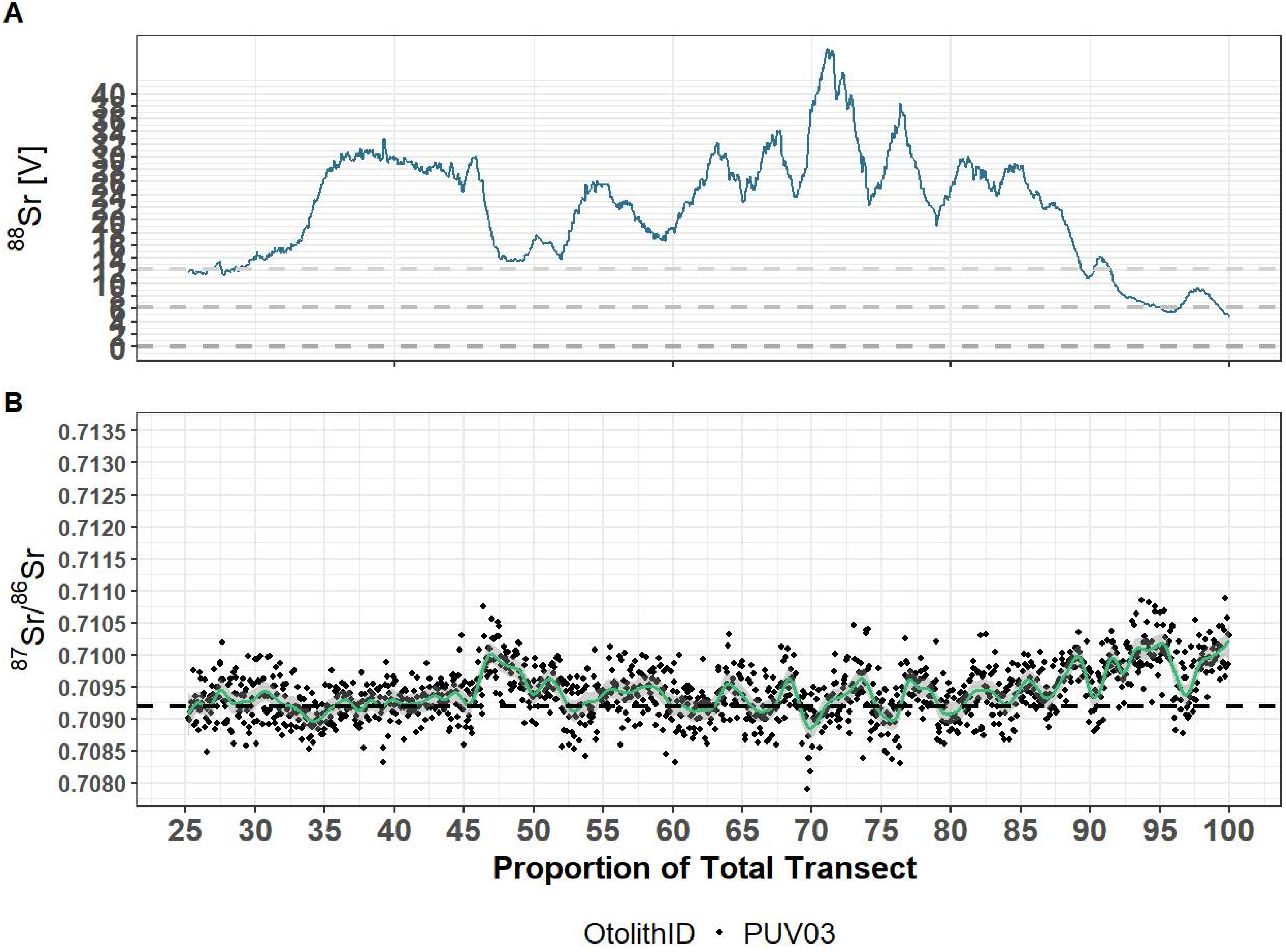

A
^{88}Sr [V]
40
0
B
^{87}Sr/^{86}Sr
0.7135
0.7130
0.7125
0.7120
0.7115
0.7110
0.7105
0.7100
0.7095
0.7090
0.7085
0.7080
25 30 35 40 45 50 55 60 65 70 75 80 85 90 95 100
Proportion of Total Transect
OtolithID • PUV03

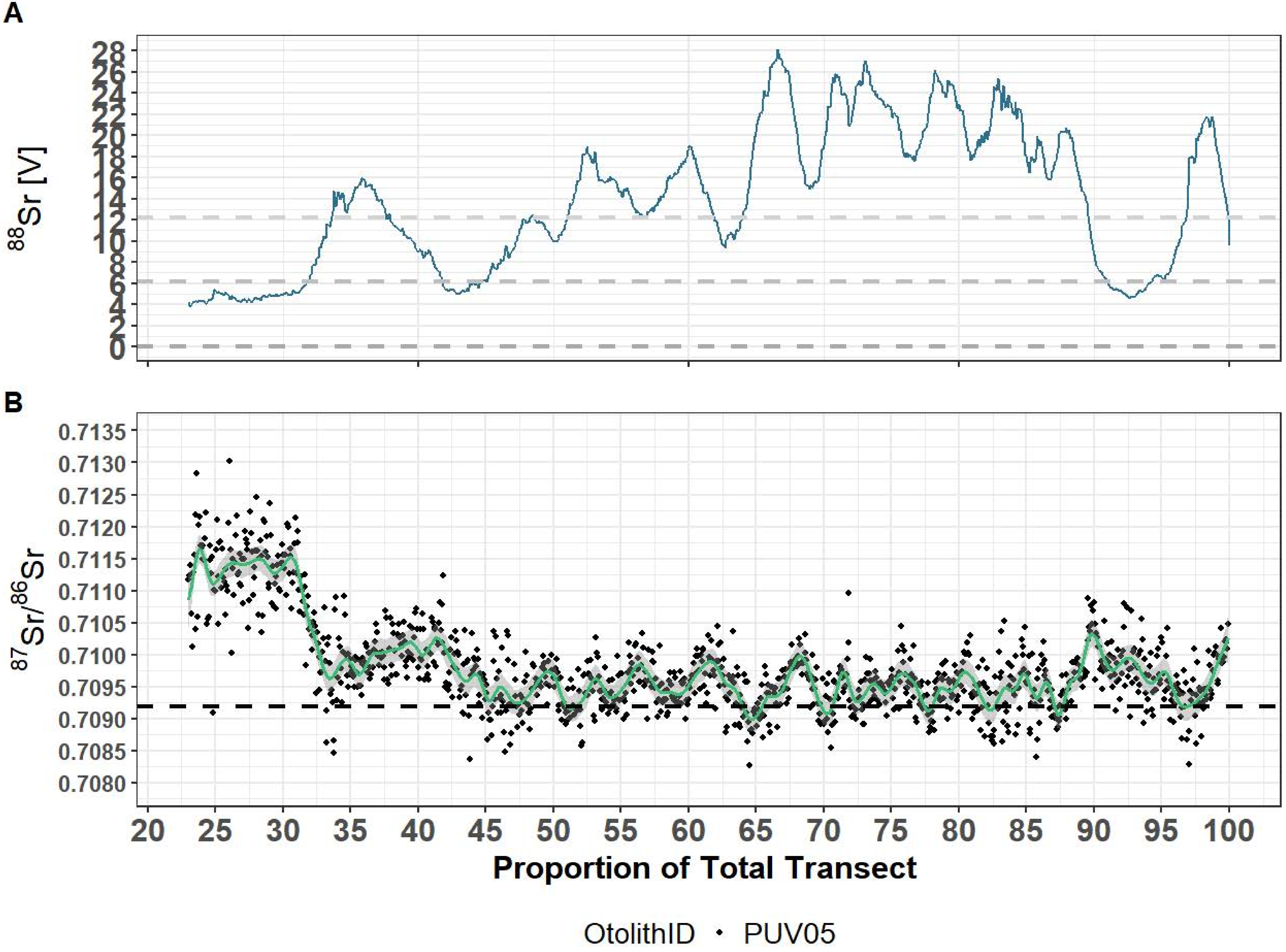

A
^{88}Sr [V]
0
2
4
6
8
10
12
14
16
18
20
22
24
26
28
B
^{87}Sr/^{86}Sr
0.7080
0.7085
0.7090
0.7095
0.7100
0.7105
0.7110
0.7115
0.7120
0.7125
0.7130
0.7135
20
25
30
35
40
45
50
55
60
65
70
75
80
85
90
95
100
Proportion of Total Transect
OtolithID • PUV05

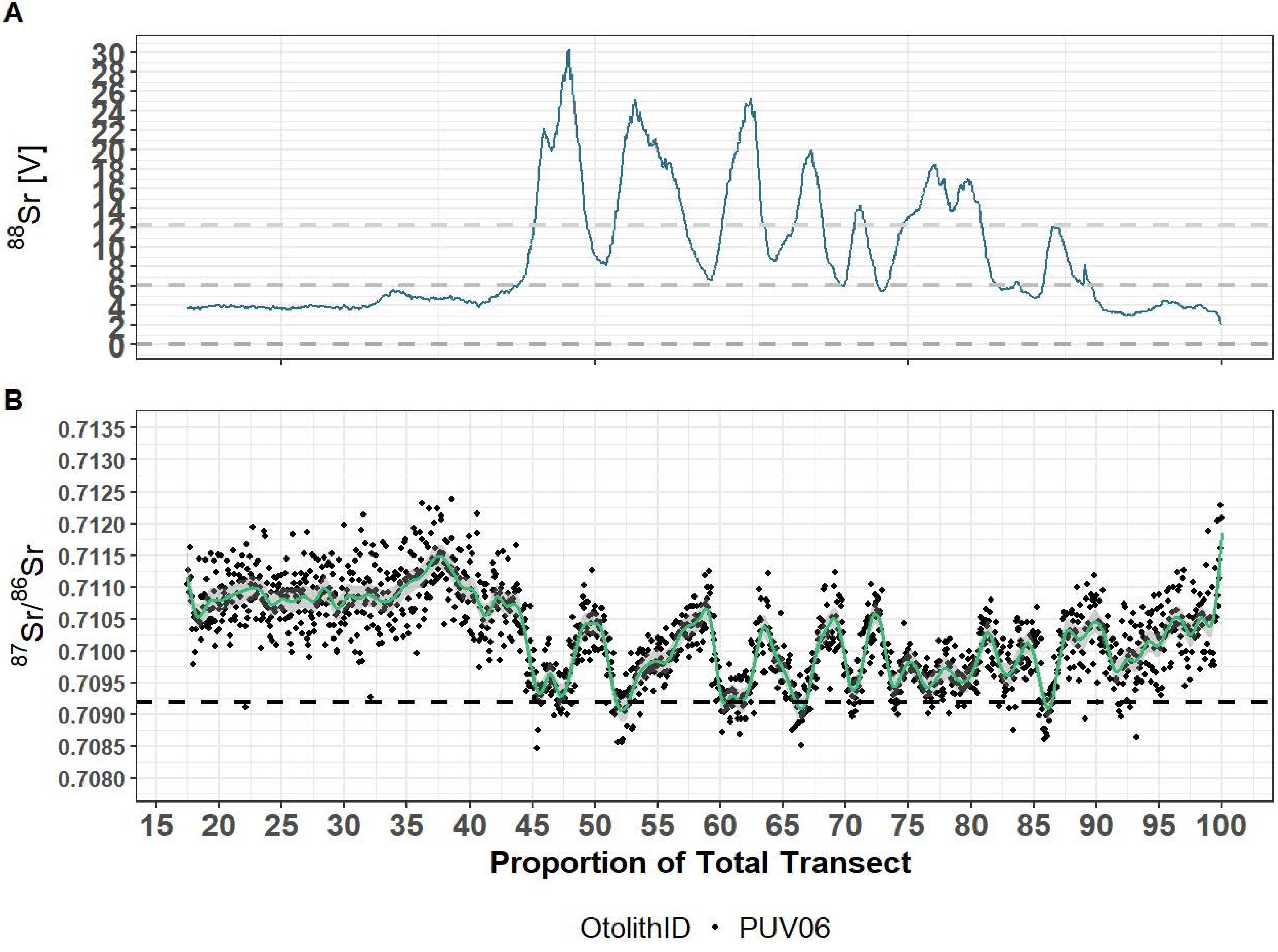

A
^{88}Sr [V]
0
2
4
6
8
10
12
14
16
18
20
22
24
26
28
30
B
$^{87}Sr/^{86}Sr$
0.7080
0.7085
0.7090
0.7095
0.7100
0.7105
0.7110
0.7115
0.7120
0.7125
0.7130
0.7135
15
20
25
30
35
40
45
50
55
60
65
70
75
80
85
90
95
100
Proportion of Total Transect
OtolithID • PUV06

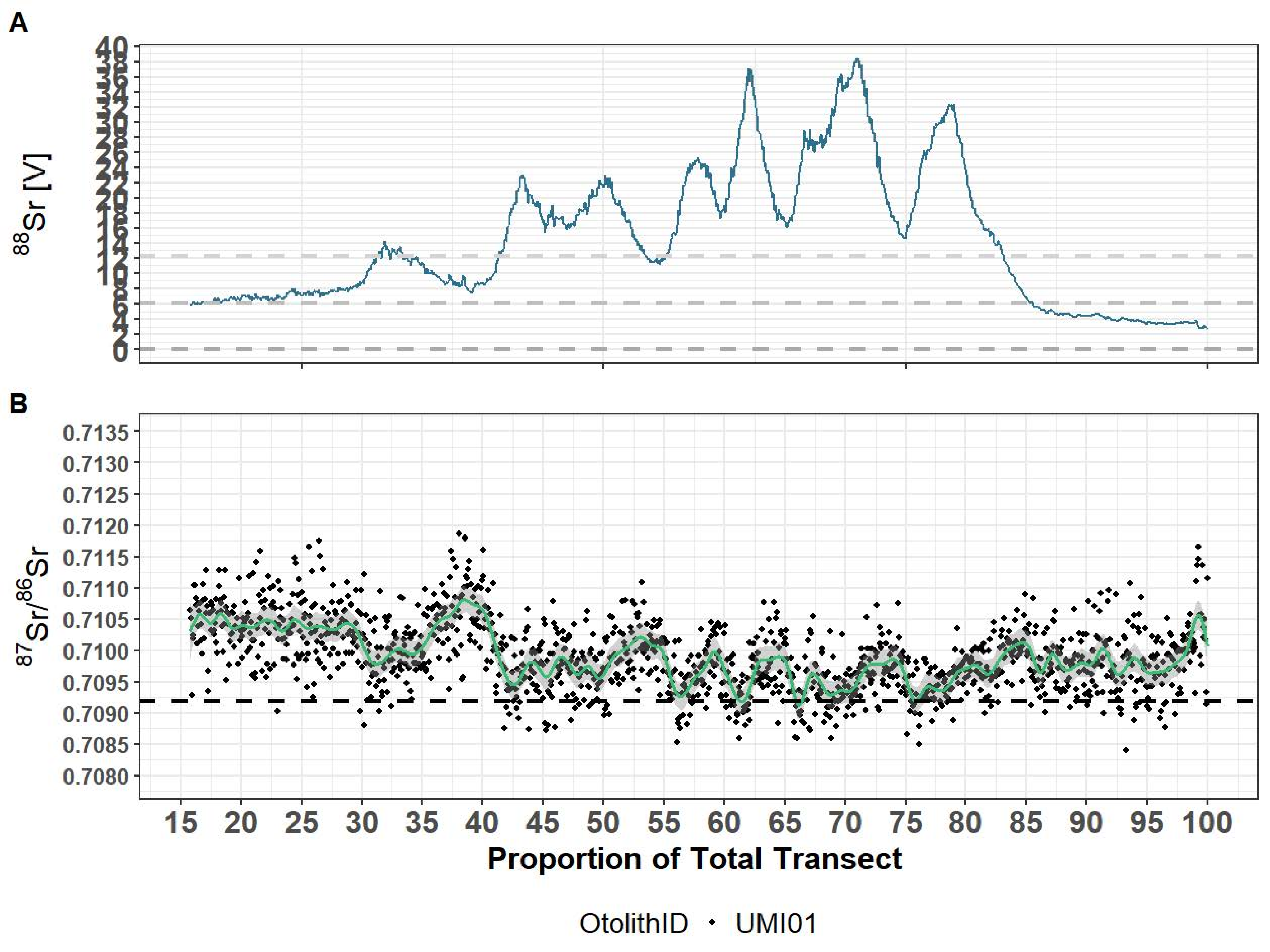
A
^{88}Sr [V]
B
$^{87}Sr/^{86}Sr$
0.7135
0.7130
0.7125
0.7120
0.7115
0.7110
0.7105
0.7100
0.7095
0.7090
0.7085
0.7080
15
20
25
30
35
40
45
50
55
60
65
70
75
80
85
90
95
100
Proportion of Total Transect
OtolithID
UMI01

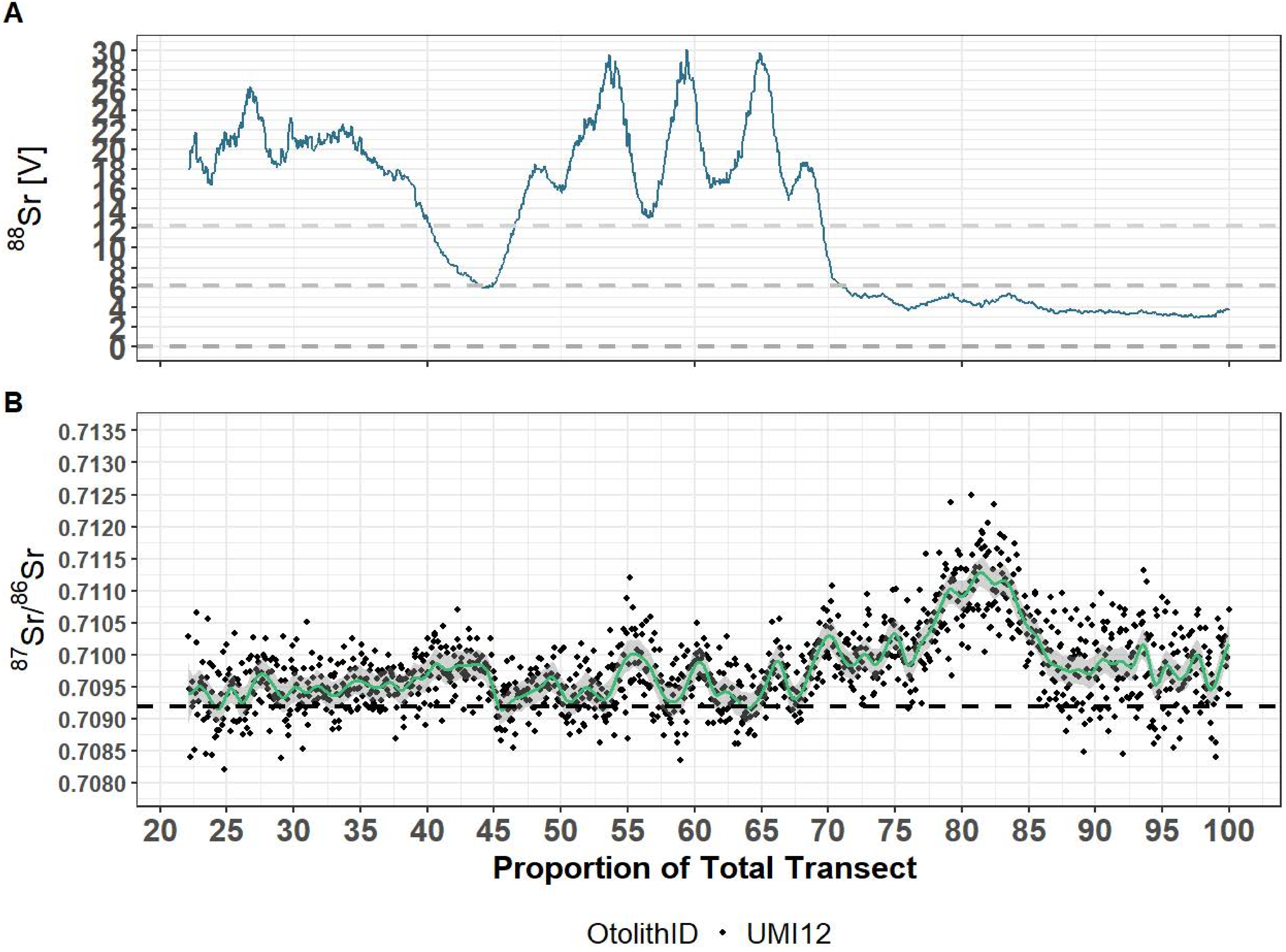
A
^{88}Sr [V]
0
2
4
6
8
10
12
14
16
18
20
22
24
26
28
30
B
$^{87}Sr/^{86}Sr$
0.7080
0.7085
0.7090
0.7095
0.7100
0.7105
0.7110
0.7115
0.7120
0.7125
0.7130
0.7135
20 25 30 35 40 45 50 55 60 65 70 75 80 85 90 95 100
Proportion of Total Transect
OtolithID • UMI12

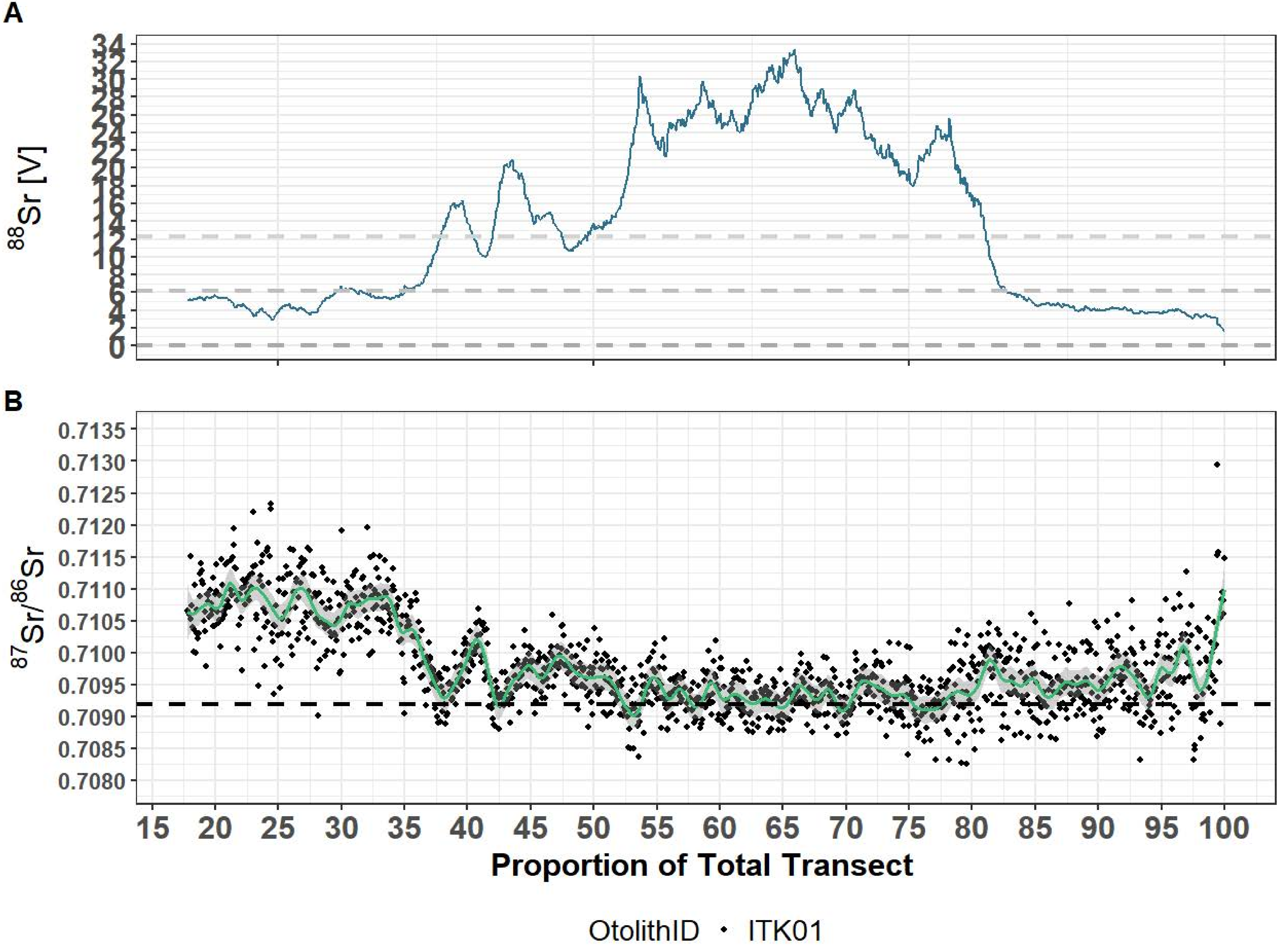

A
^{88}Sr [V]
0
2
4
6
8
10
12
14
16
18
20
22
24
26
28
30
32
34
B
^{87}Sr/^{86}Sr
0.7080
0.7085
0.7090
0.7095
0.7100
0.7105
0.7110
0.7115
0.7120
0.7125
0.7130
0.7135
15 20 25 30 35 40 45 50 55 60 65 70 75 80 85 90 95 100
Proportion of Total Transect
OtolithID • ITK01

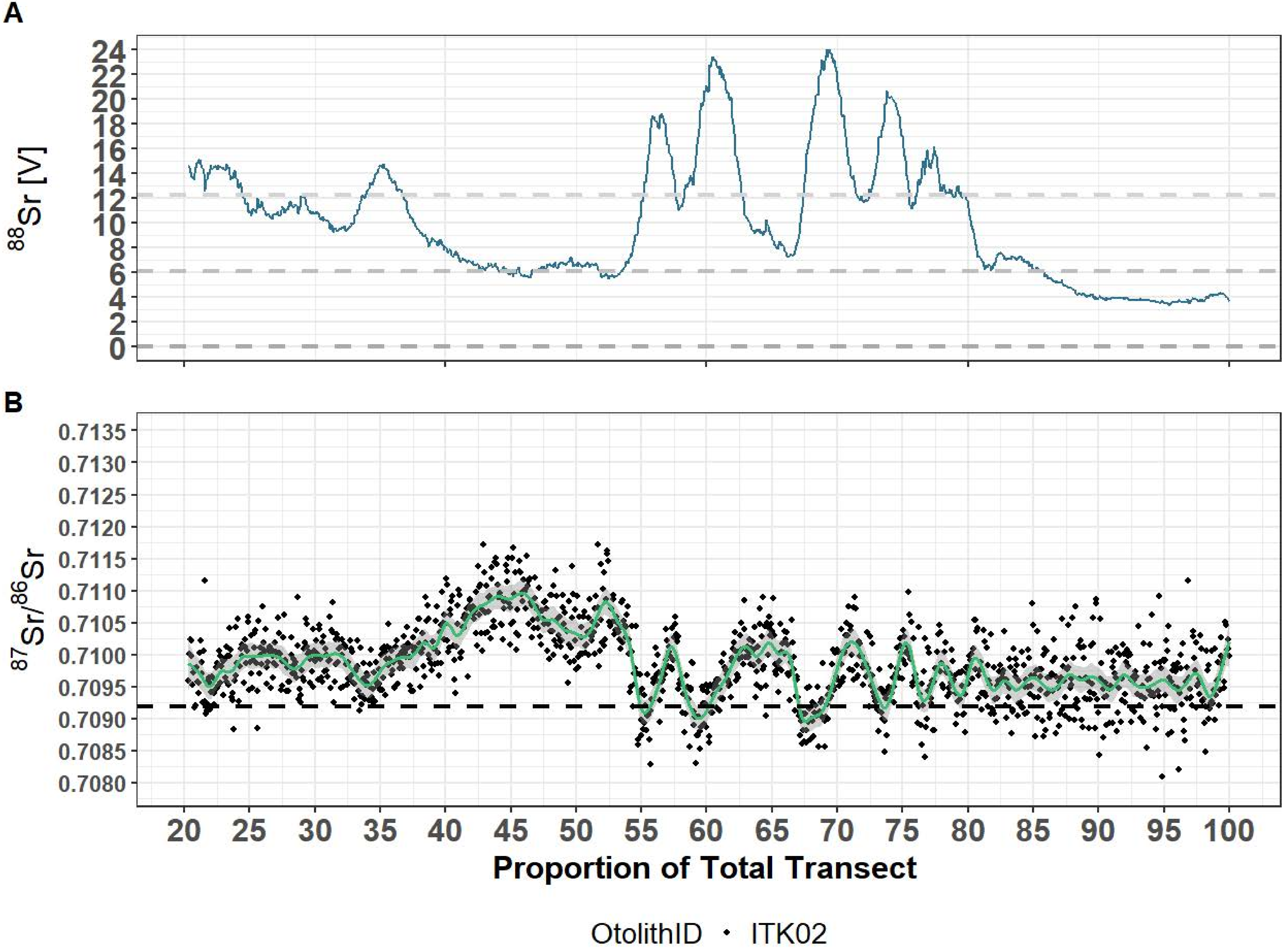

A
^{88}Sr [V]
0
2
4
6
8
10
12
14
16
18
20
22
24
B
^{87}Sr/^{86}Sr
0.7080
0.7085
0.7090
0.7095
0.7100
0.7105
0.7110
0.7115
0.7120
0.7125
0.7130
0.7135
20
25
30
35
40
45
50
55
60
65
70
75
80
85
90
95
100
Proportion of Total Transect
OtolithID
ITK02

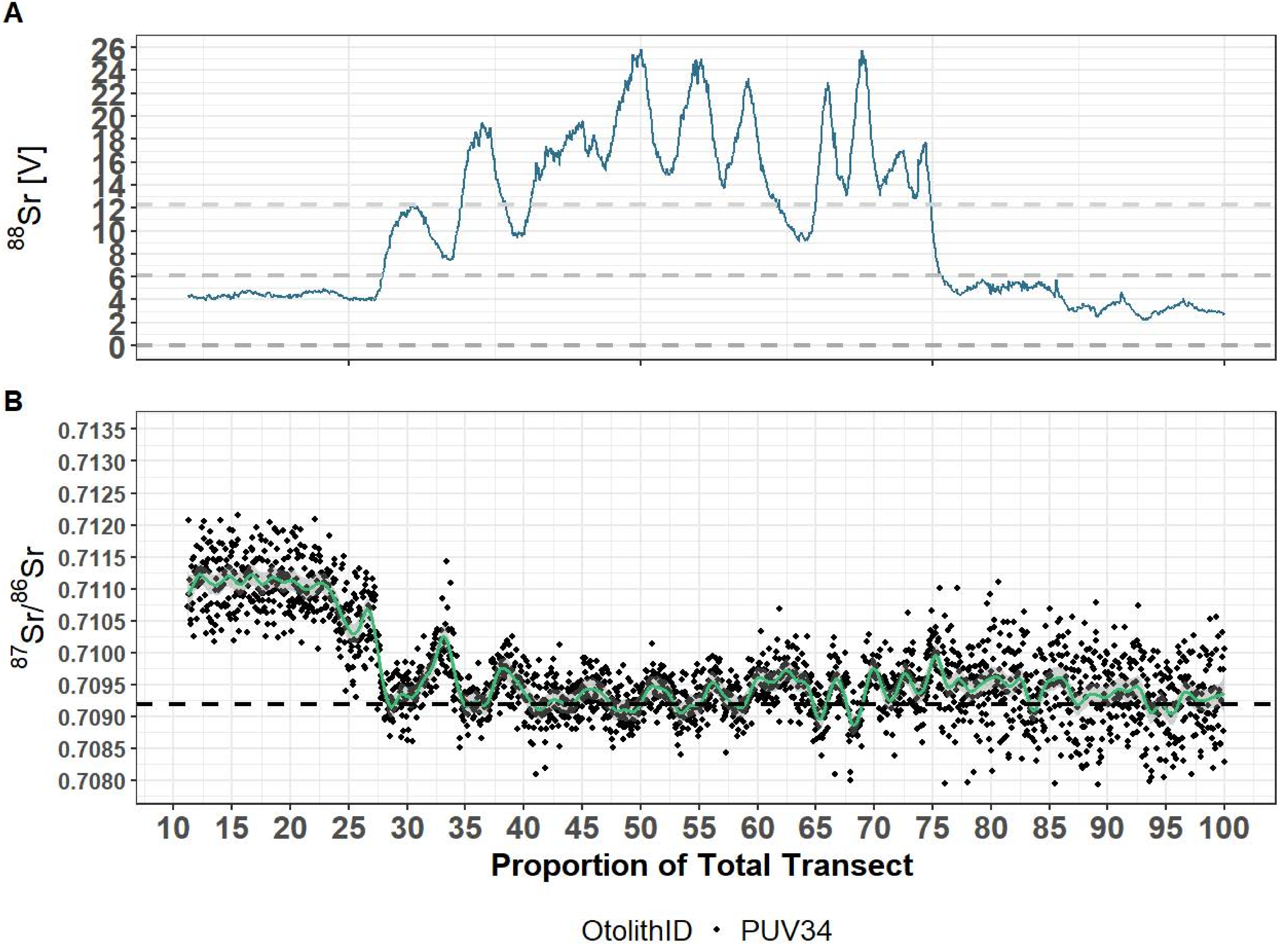
A
^{88}Sr [V]
0
2
4
6
8
10
12
14
16
18
20
22
24
26
B
^{87}Sr/^{86}Sr
0.7080
0.7085
0.7090
0.7095
0.7100
0.7105
0.7110
0.7115
0.7120
0.7125
0.7130
0.7135
10 15 20 25 30 35 40 45 50 55 60 65 70 75 80 85 90 95 100
Proportion of Total Transect
OtolithID • PUV34

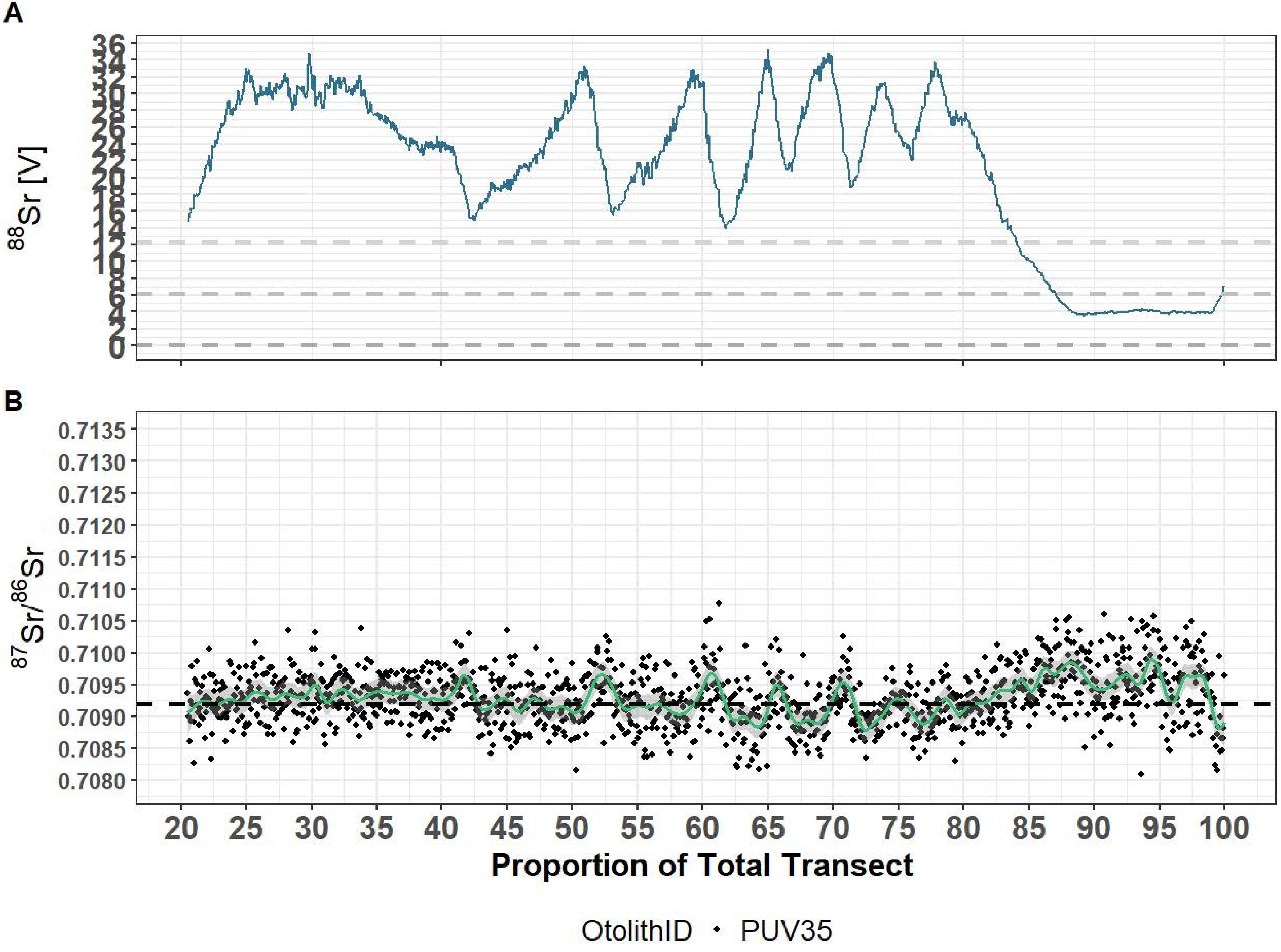

A
^{88}Sr [V]
36
34
32
30
28
26
24
22
20
18
16
14
12
10
8
6
4
2
0
B
^{87}Sr/^{86}Sr
0.7135
0.7130
0.7125
0.7120
0.7115
0.7110
0.7105
0.7100
0.7095
0.7090
0.7085
0.7080
20
25
30
35
40
45
50
55
60
65
70
75
80
85
90
95
100
Proportion of Total Transect
OtolithID • PUV35

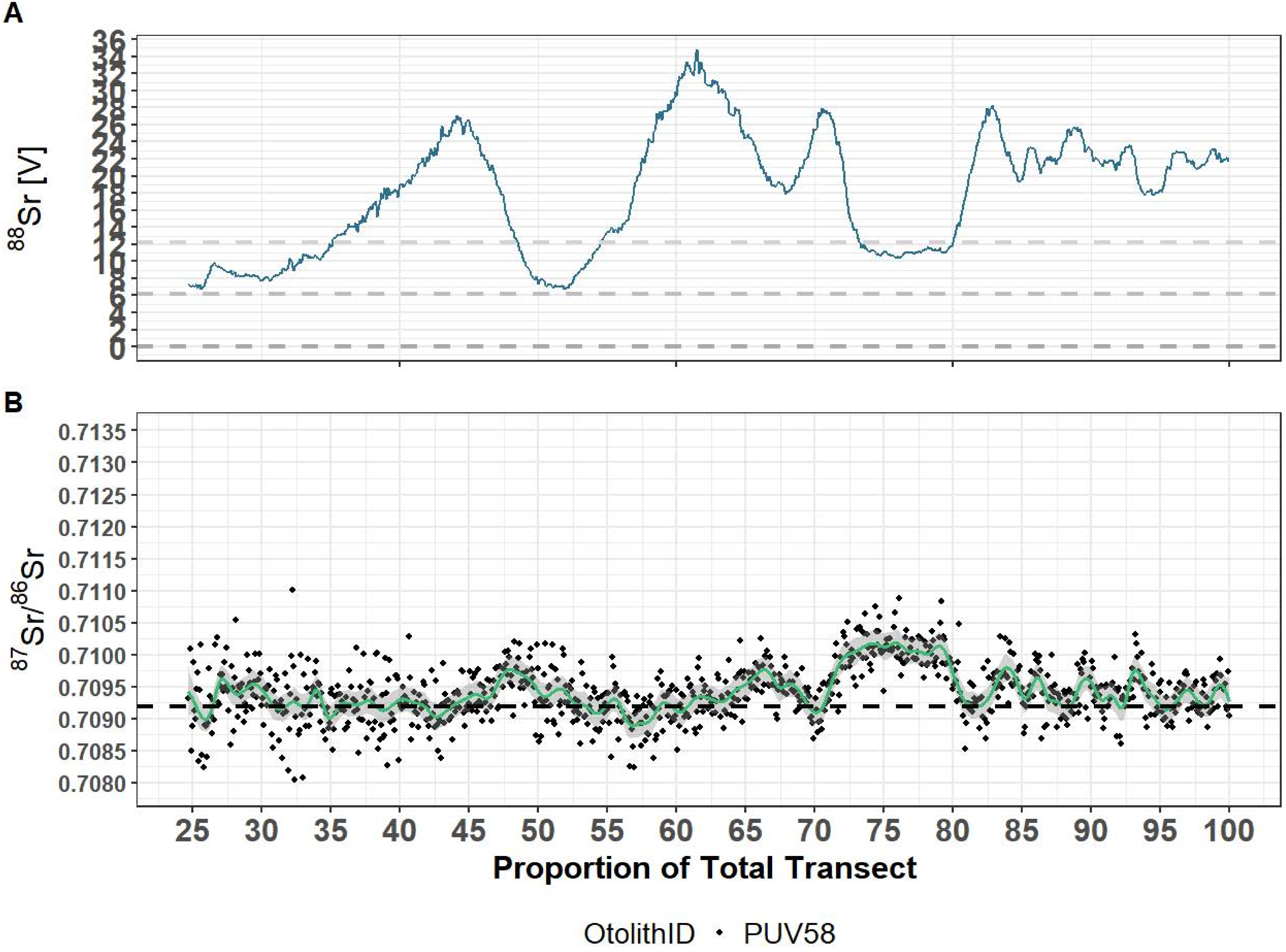

A
^{88}Sr [V]
0
2
4
6
8
10
12
14
16
18
20
22
24
26
28
30
32
34
36
B
^{87}Sr/^{86}Sr
0.7080
0.7085
0.7090
0.7095
0.7100
0.7105
0.7110
0.7115
0.7120
0.7125
0.7130
0.7135
25
30
35
40
45
50
55
60
65
70
75
80
85
90
95
100
Proportion of Total Transect
OtolithID • PUV58

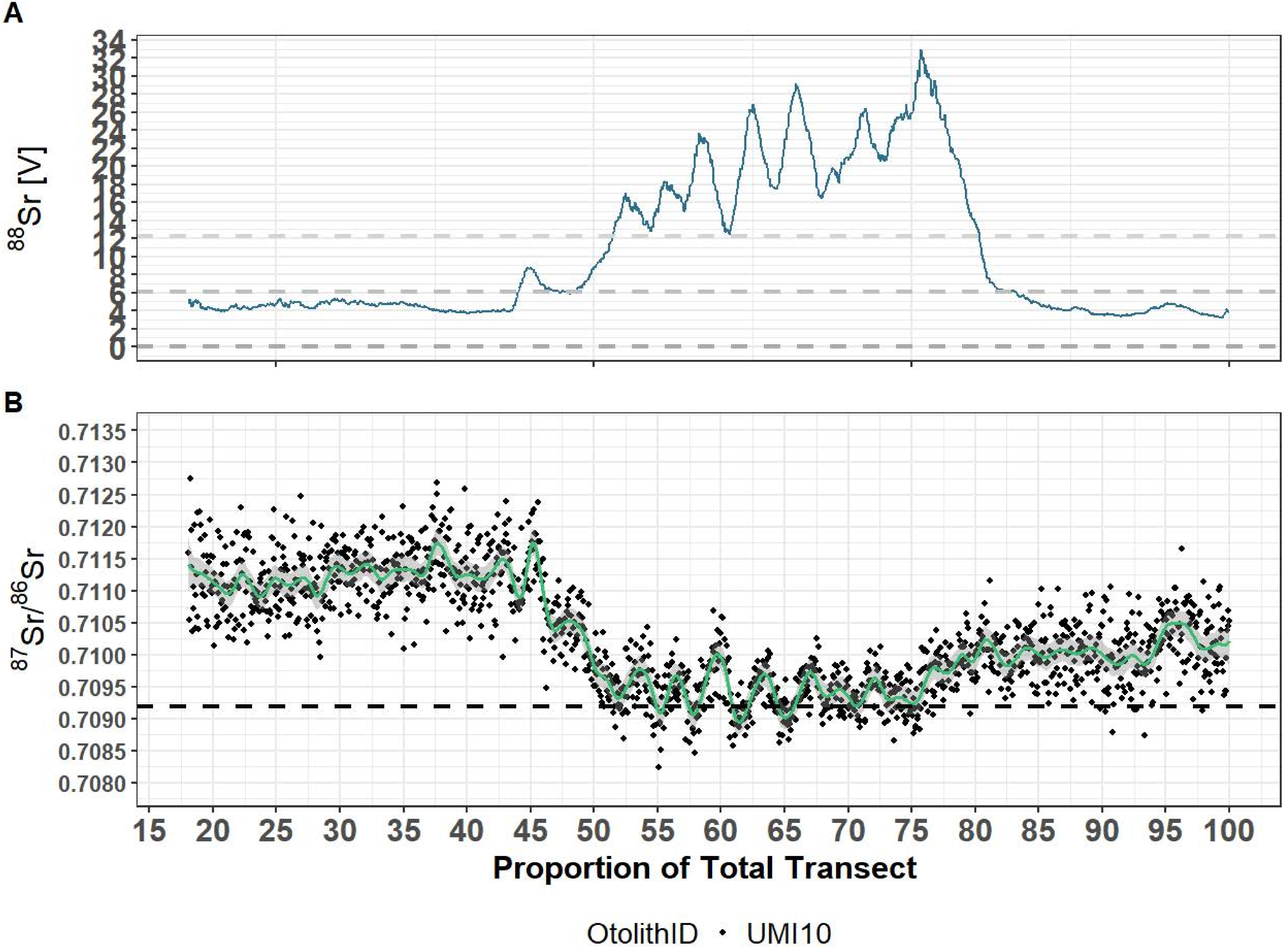

A
^{88}Sr [V]
0
2
4
6
8
10
12
14
16
18
20
22
24
26
28
30
32
34
B
^{87}Sr/^{86}Sr
0.7080
0.7085
0.7090
0.7095
0.7100
0.7105
0.7110
0.7115
0.7120
0.7125
0.7130
0.7135
15
20
25
30
35
40
45
50
55
60
65
70
75
80
85
90
95
100
Proportion of Total Transect
OtolithID • UMI10

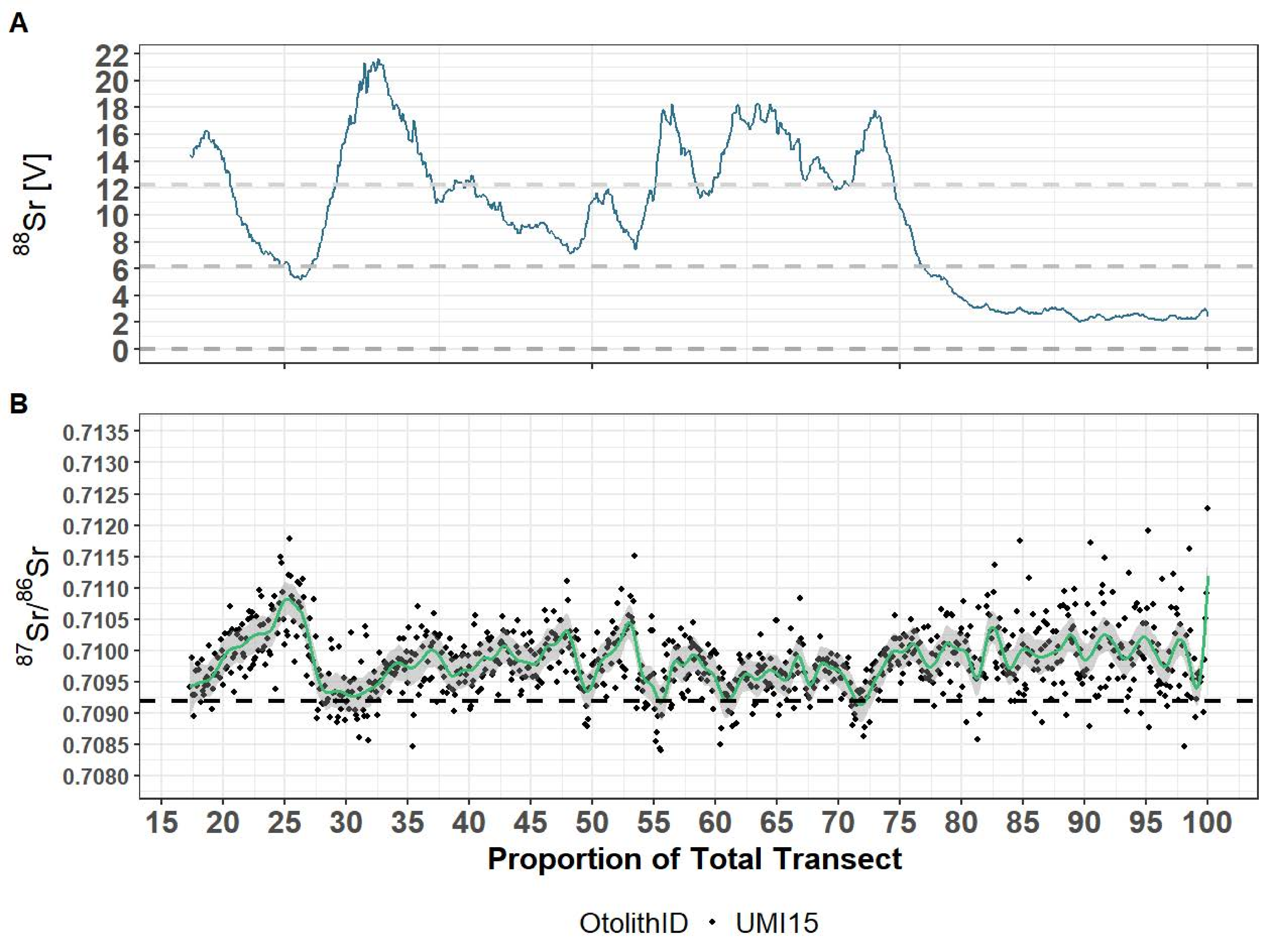
A
B
22
20
18
16
14
12
10
8
6
4
2
0
^{88}Sr [V]
0.7135
0.7130
0.7125
0.7120
0.7115
0.7110
0.7105
0.7100
0.7095
0.7090
0.7085
0.7080
^{87}Sr/^{86}Sr
15 20 25 30 35 40 45 50 55 60 65 70 75 80 85 90 95 100
Proportion of Total Transect
OtolithID • UMI15

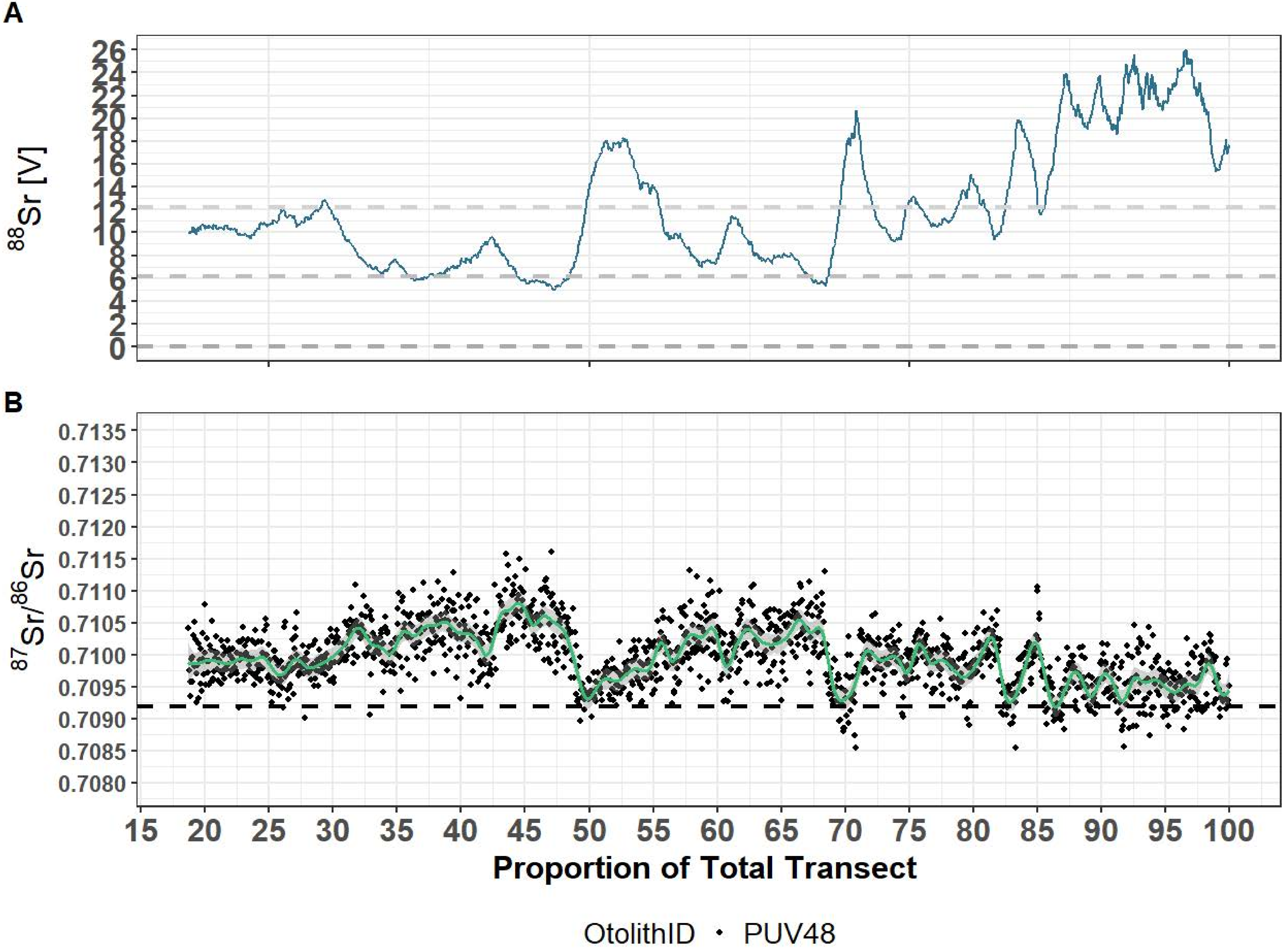

A
26
24
22
20
18
16
14
12
10
8
6
4
2
0
^{88}Sr [V]
B
0.7135
0.7130
0.7125
0.7120
0.7115
0.7110
0.7105
0.7100
0.7095
0.7090
0.7085
0.7080
^{87}Sr/^{86}Sr
15 20 25 30 35 40 45 50 55 60 65 70 75 80 85 90 95 100
Proportion of Total Transect
OtolithID • PUV48

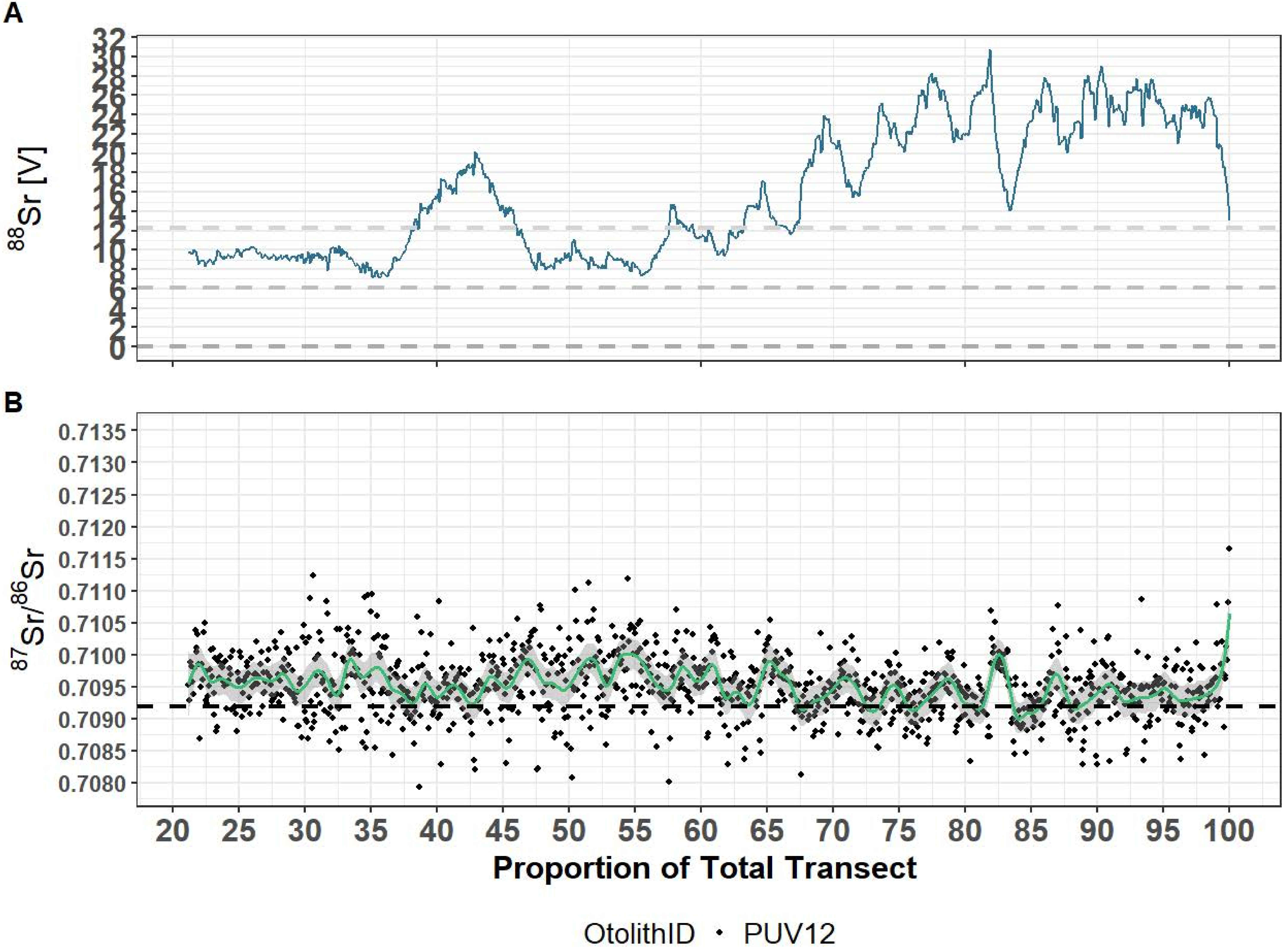

A
88Sr [V]
0
2
4
6
8
10
12
14
16
18
20
22
24
26
28
30
32
B
87Sr/86Sr
0.7080
0.7085
0.7090
0.7095
0.7100
0.7105
0.7110
0.7115
0.7120
0.7125
0.7130
0.7135
20
25
30
35
40
45
50
55
60
65
70
75
80
85
90
95
100
Proportion of Total Transect
OtolithID
PUV12

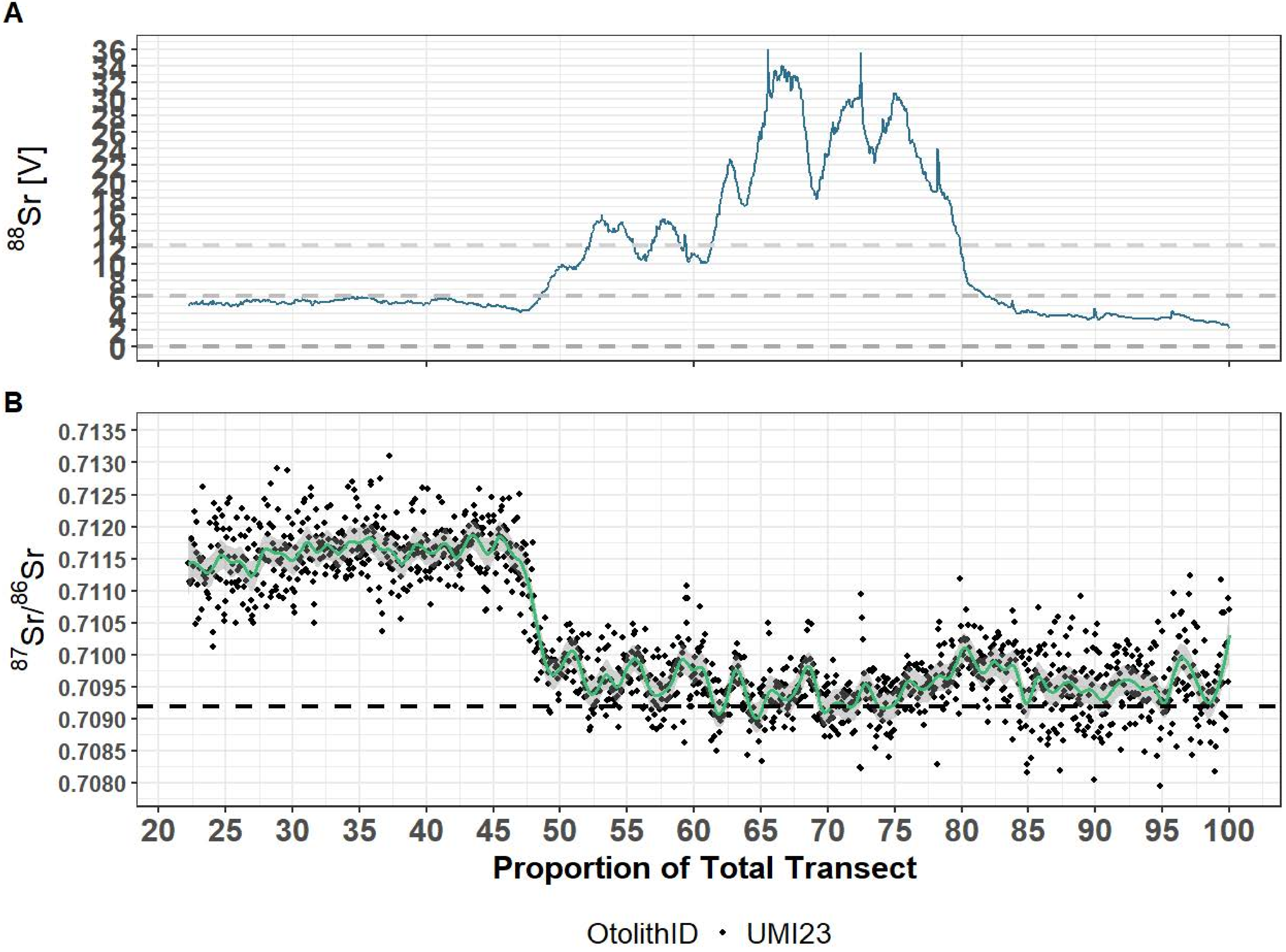

A
^{88}Sr [V]
B
$^{87}Sr/^{86}Sr$
0.7135
0.7130
0.7125
0.7120
0.7115
0.7110
0.7105
0.7100
0.7095
0.7090
0.7085
0.7080
20 25 30 35 40 45 50 55 60 65 70 75 80 85 90 95 100
Proportion of Total Transect
OtolithID • UMI23

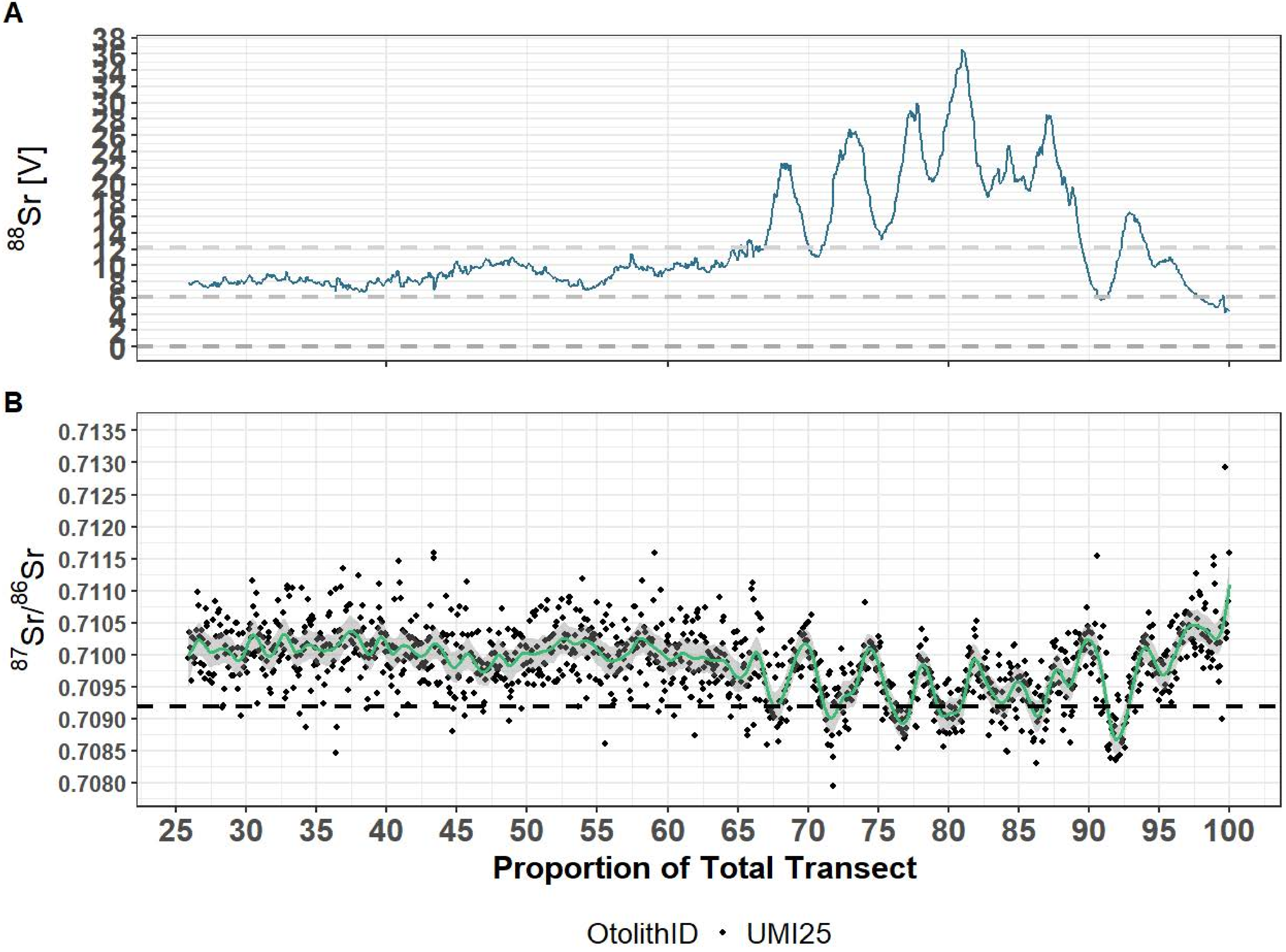
A
^{88}Sr [V]
0
2
4
6
8
10
12
14
16
18
20
22
24
26
28
30
32
34
36
38
B
^{87}Sr/^{86}Sr
0.7080
0.7085
0.7090
0.7095
0.7100
0.7105
0.7110
0.7115
0.7120
0.7125
0.7130
0.7135
25
30
35
40
45
50
55
60
65
70
75
80
85
90
95
100
Proportion of Total Transect
OtolithID • UMI25

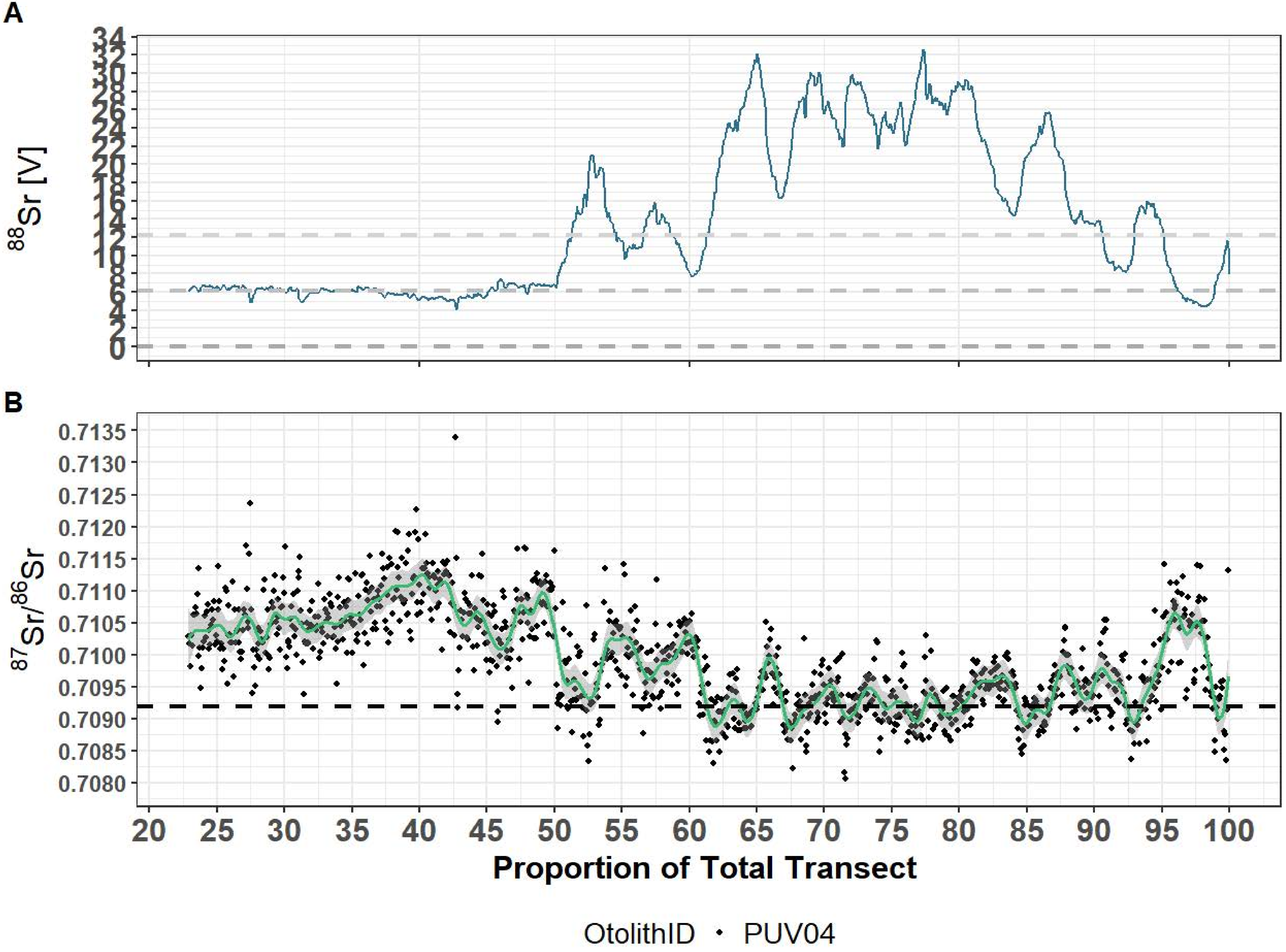
A
B
^{88}Sr [V]
34
32
30
28
26
24
22
20
18
16
14
12
10
8
6
4
2
0
^{87}Sr/^{86}Sr
0.7135
0.7130
0.7125
0.7120
0.7115
0.7110
0.7105
0.7100
0.7095
0.7090
0.7085
0.7080
20
25
30
35
40
45
50
55
60
65
70
75
80
85
90
95
100
Proportion of Total Transect
OtolithID • PUV04

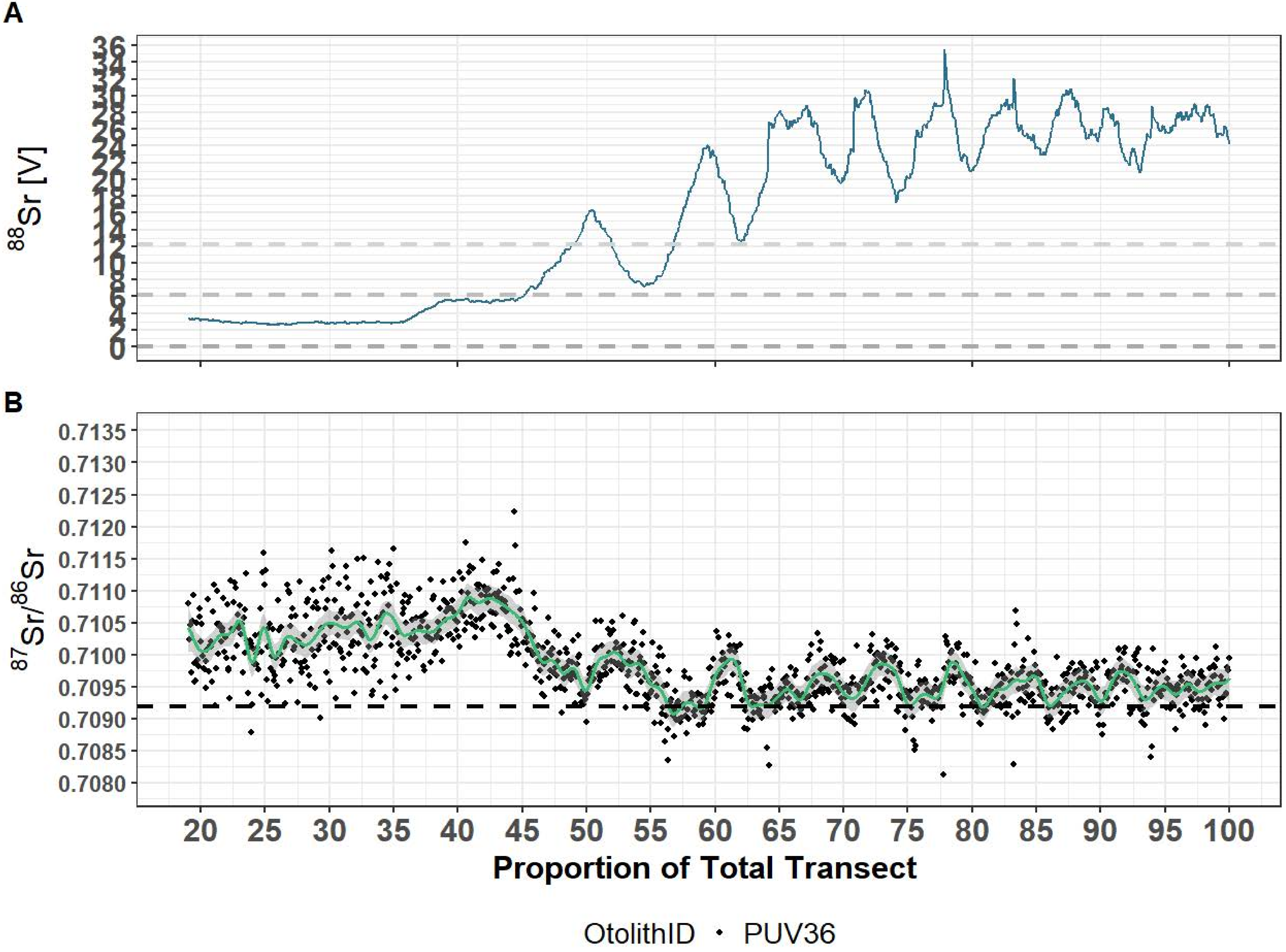

A
^{88}Sr [V]
0
2
4
6
8
10
12
14
16
18
20
22
24
26
28
30
32
34
36
B
^{87}Sr/^{86}Sr
0.7080
0.7085
0.7090
0.7095
0.7100
0.7105
0.7110
0.7115
0.7120
0.7125
0.7130
0.7135
20
25
30
35
40
45
50
55
60
65
70
75
80
85
90
95
100
Proportion of Total Transect
OtolithID • PUV36

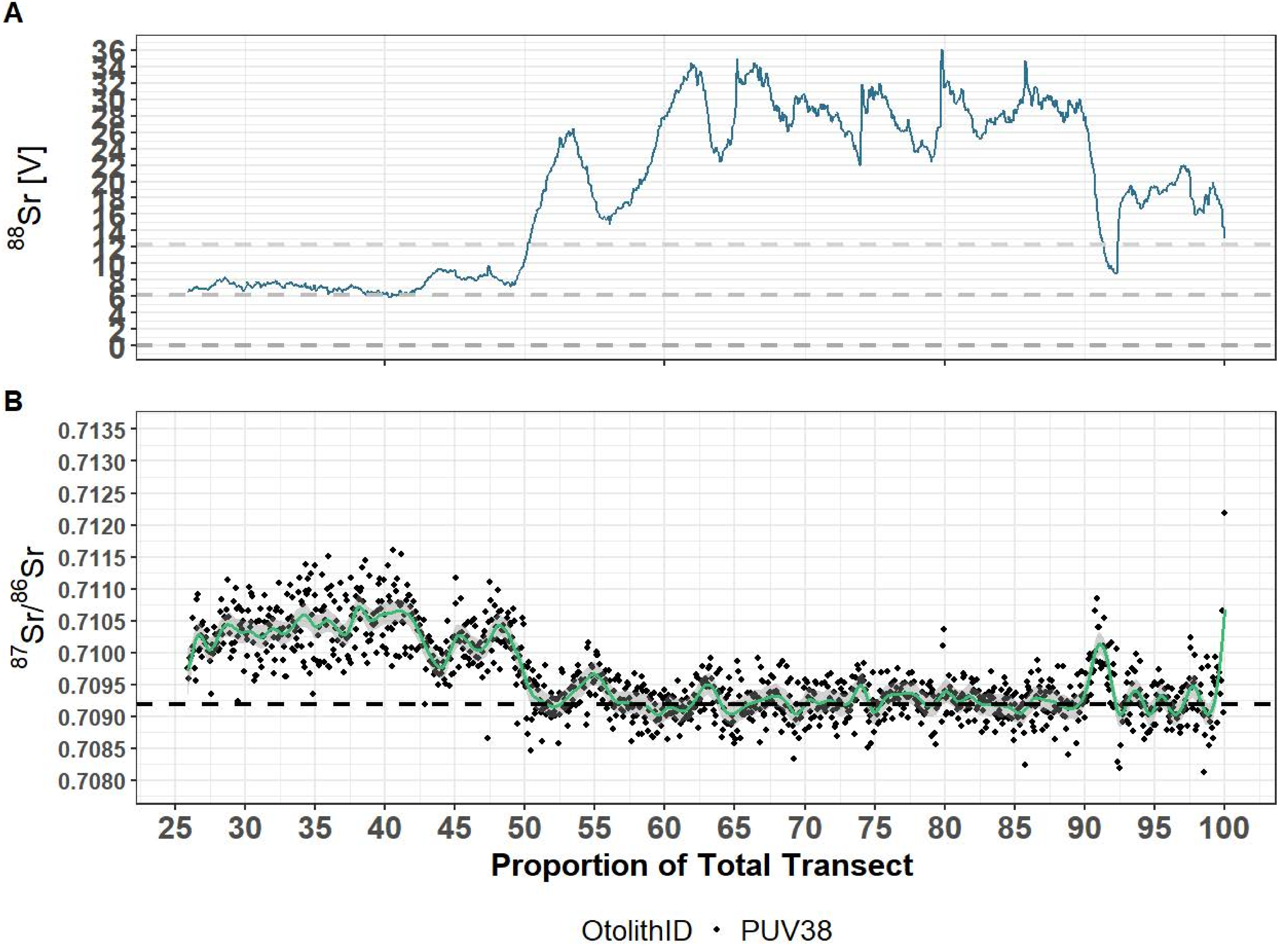

A
^{88}Sr [V]
36
34
32
30
28
26
24
22
20
18
16
14
12
10
8
6
4
2
0
B
^{87}Sr/^{86}Sr
0.7135
0.7130
0.7125
0.7120
0.7115
0.7110
0.7105
0.7100
0.7095
0.7090
0.7085
0.7080
25
30
35
40
45
50
55
60
65
70
75
80
85
90
95
100
Proportion of Total Transect
OtolithID
PUV38

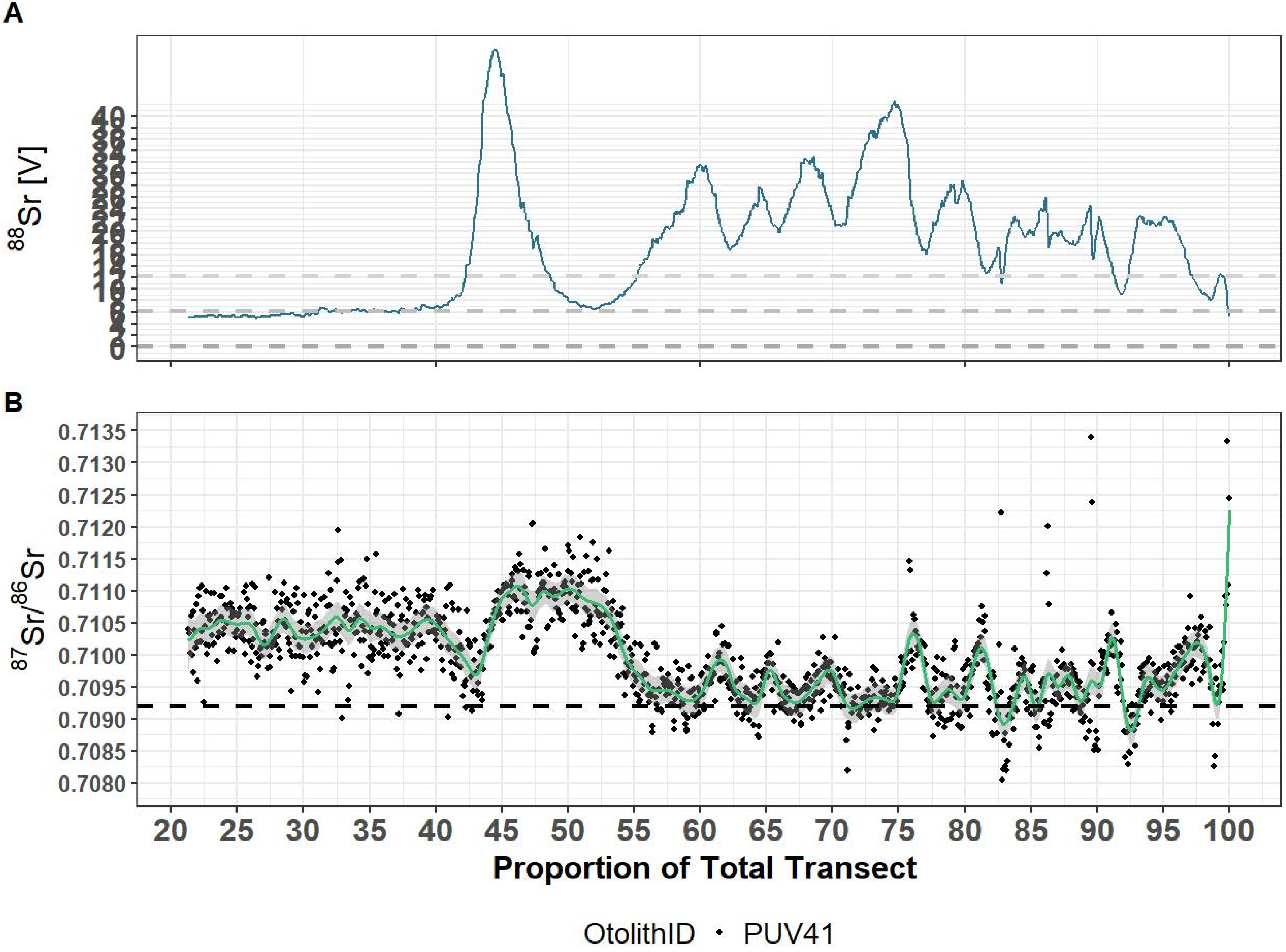
A
B
^{88}Sr [V]
40
0
$^{87}Sr/^{86}Sr$
0.7135
0.7130
0.7125
0.7120
0.7115
0.7110
0.7105
0.7100
0.7095
0.7090
0.7085
0.7080
20 25 30 35 40 45 50 55 60 65 70 75 80 85 90 95 100
Proportion of Total Transect
OtolithID • PUV41

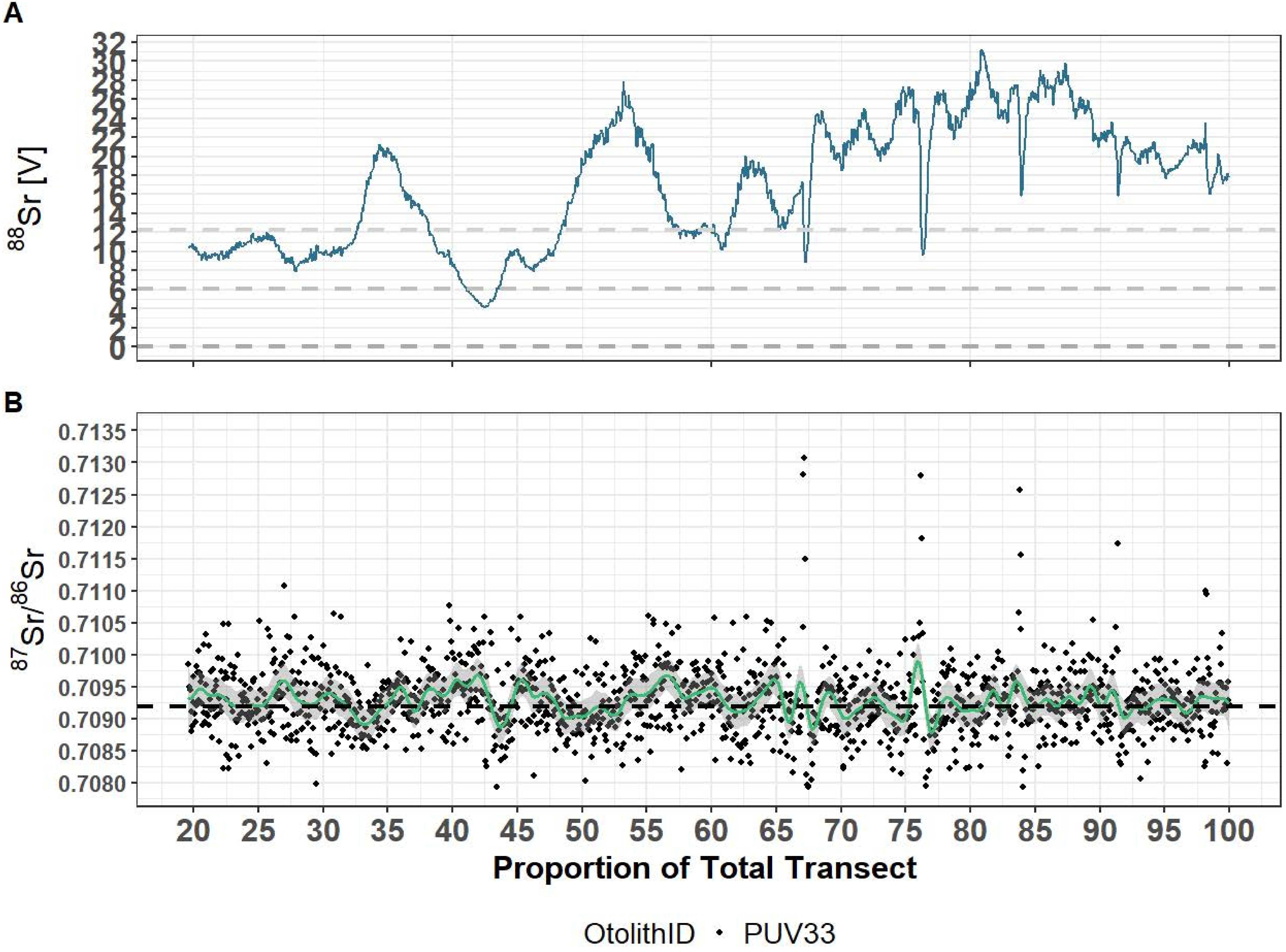

A
^{88}Sr [V]
0
2
4
6
8
10
12
14
16
18
20
22
24
26
28
30
32
B
^{87}Sr/^{86}Sr
0.7080
0.7085
0.7090
0.7095
0.7100
0.7105
0.7110
0.7115
0.7120
0.7125
0.7130
0.7135
20
25
30
35
40
45
50
55
60
65
70
75
80
85
90
95
100
Proportion of Total Transect
OtolithID
PUV33

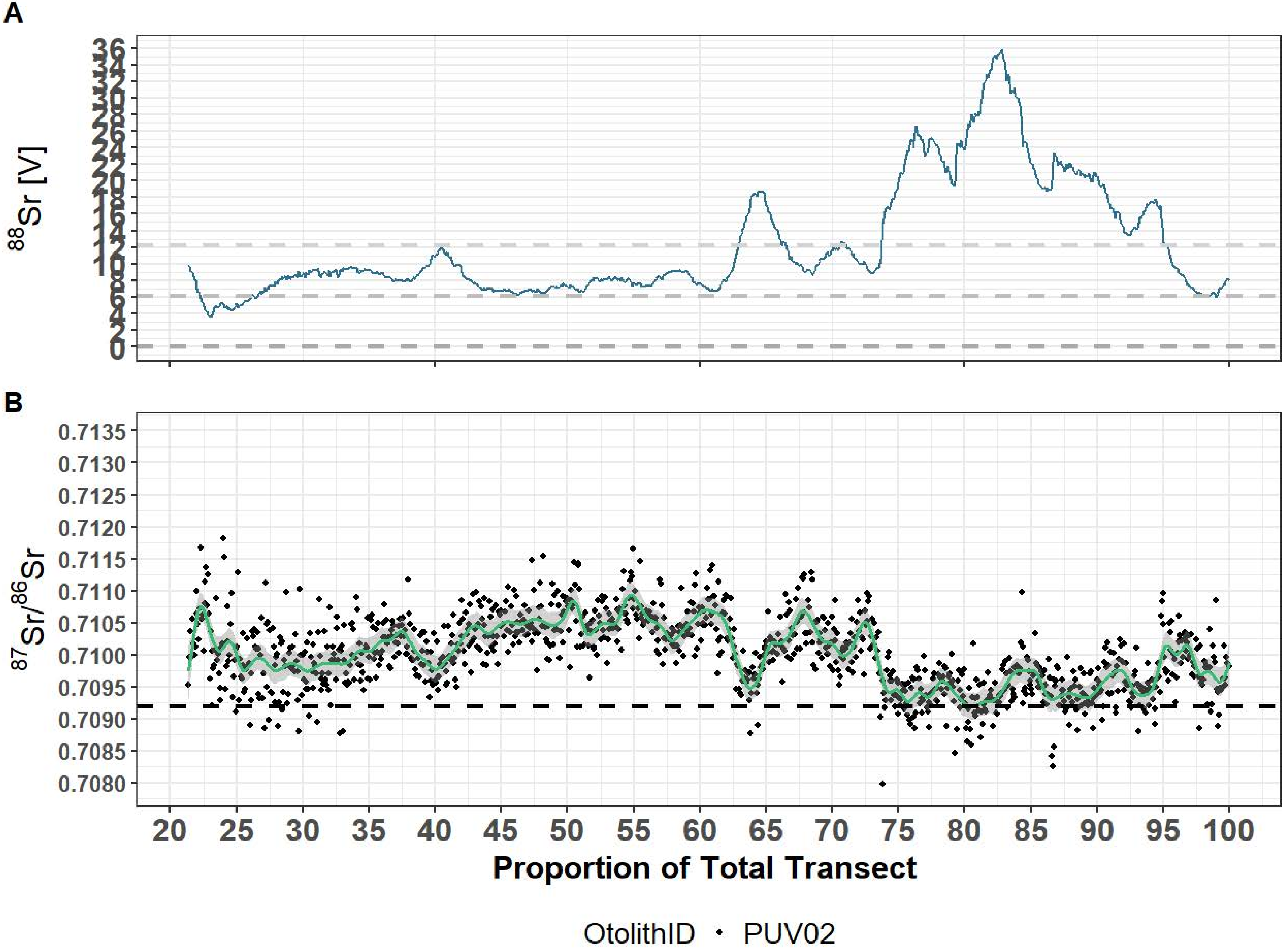
A
88Sr [V]
0
2
4
6
8
10
12
14
16
18
20
22
24
26
28
30
32
34
36
B
87Sr/86Sr
0.7080
0.7085
0.7090
0.7095
0.7100
0.7105
0.7110
0.7115
0.7120
0.7125
0.7130
0.7135
20 25 30 35 40 45 50 55 60 65 70 75 80 85 90 95 100
Proportion of Total Transect
OtolithID • PUV02

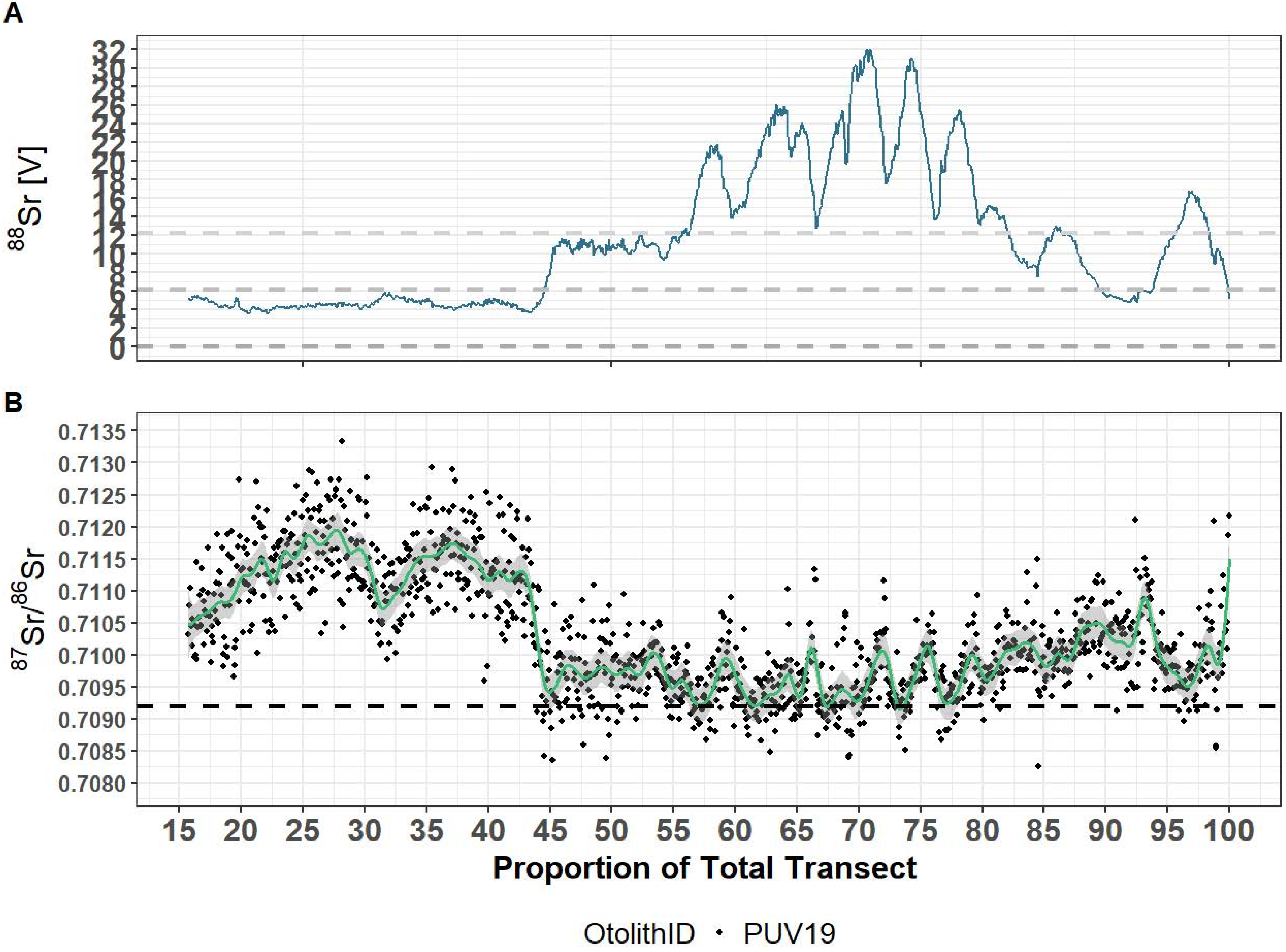

A
88Sr [V]
0
2
4
6
8
10
12
14
16
18
20
22
24
26
28
30
32
B
87Sr/86Sr
0.7080
0.7085
0.7090
0.7095
0.7100
0.7105
0.7110
0.7115
0.7120
0.7125
0.7130
0.7135
15 20 25 30 35 40 45 50 55 60 65 70 75 80 85 90 95 100
Proportion of Total Transect
OtolithID • PUV19

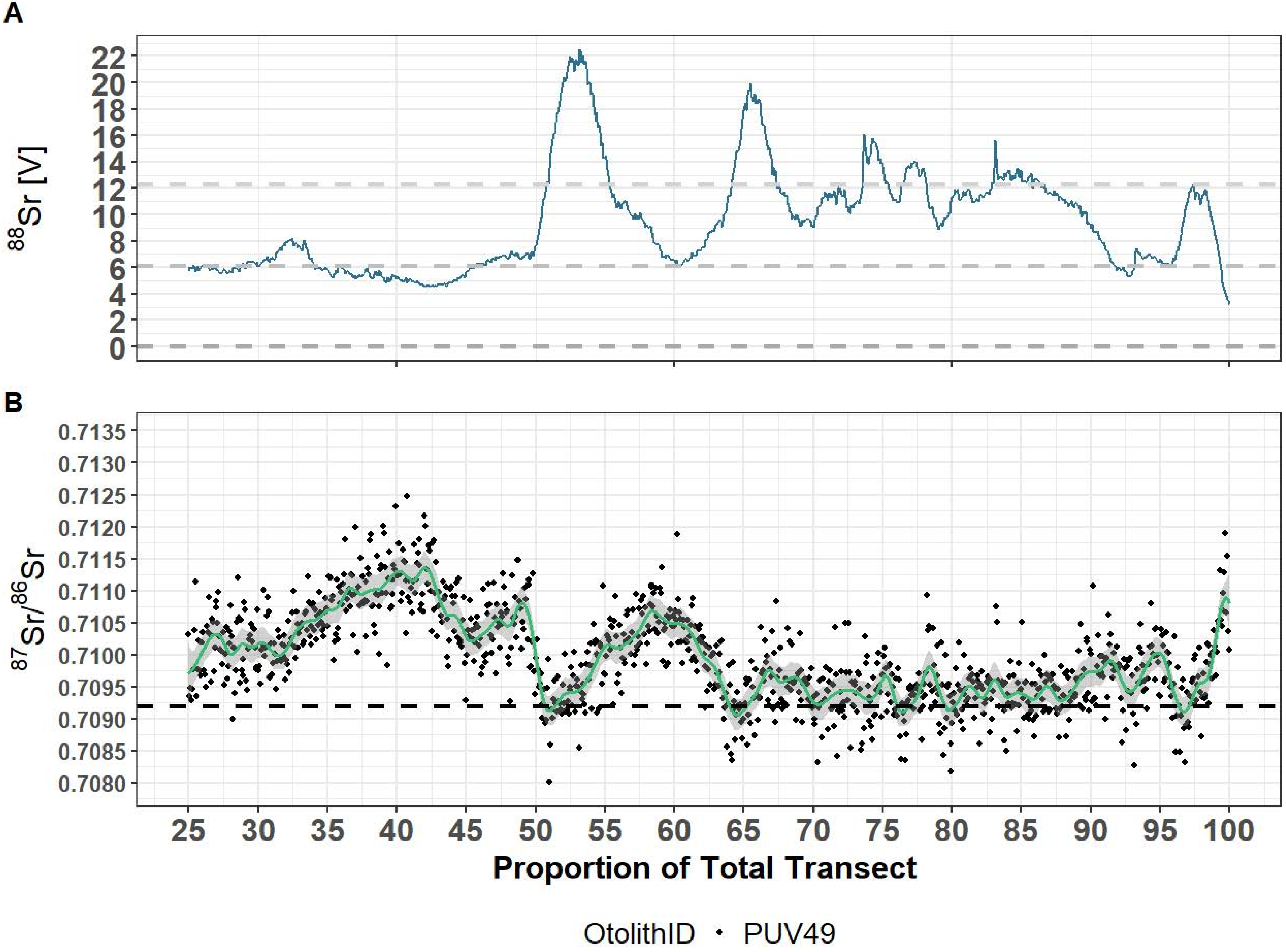
A
^{88}Sr [V]
0
2
4
6
8
10
12
14
16
18
20
22
B
^{87}Sr/^{86}Sr
0.7080
0.7085
0.7090
0.7095
0.7100
0.7105
0.7110
0.7115
0.7120
0.7125
0.7130
0.7135
25
30
35
40
45
50
55
60
65
70
75
80
85
90
95
100
Proportion of Total Transect
OtolithID • PUV49

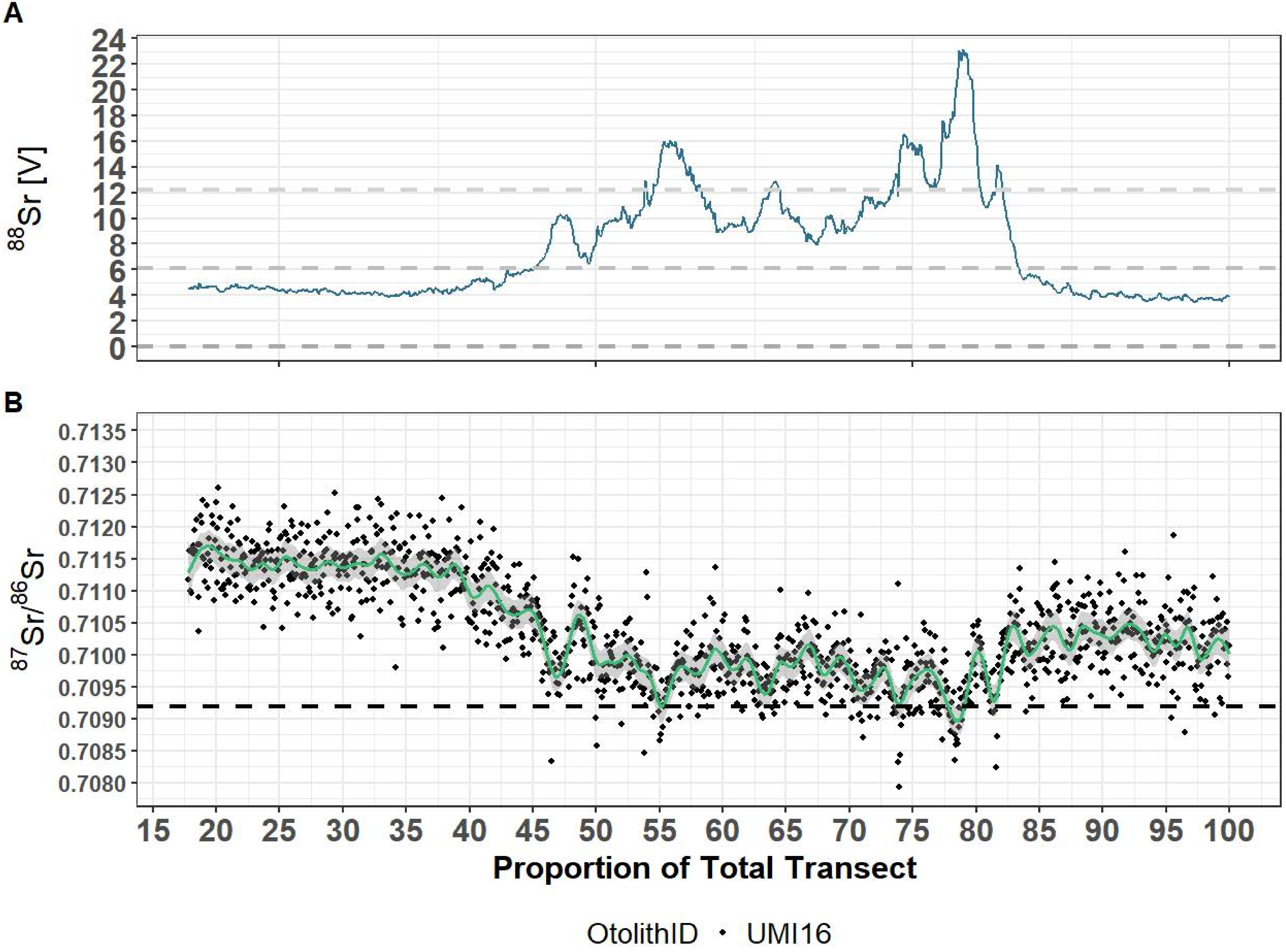

A
24
22
20
18
16
14
12
10
8
6
4
2
0
^{88}Sr [V]
B
0.7135
0.7130
0.7125
0.7120
0.7115
0.7110
0.7105
0.7100
0.7095
0.7090
0.7085
0.7080
$^{87}Sr/^{86}Sr$
15
20
25
30
35
40
45
50
55
60
65
70
75
80
85
90
95
100
Proportion of Total Transect
OtolithID
UMI16

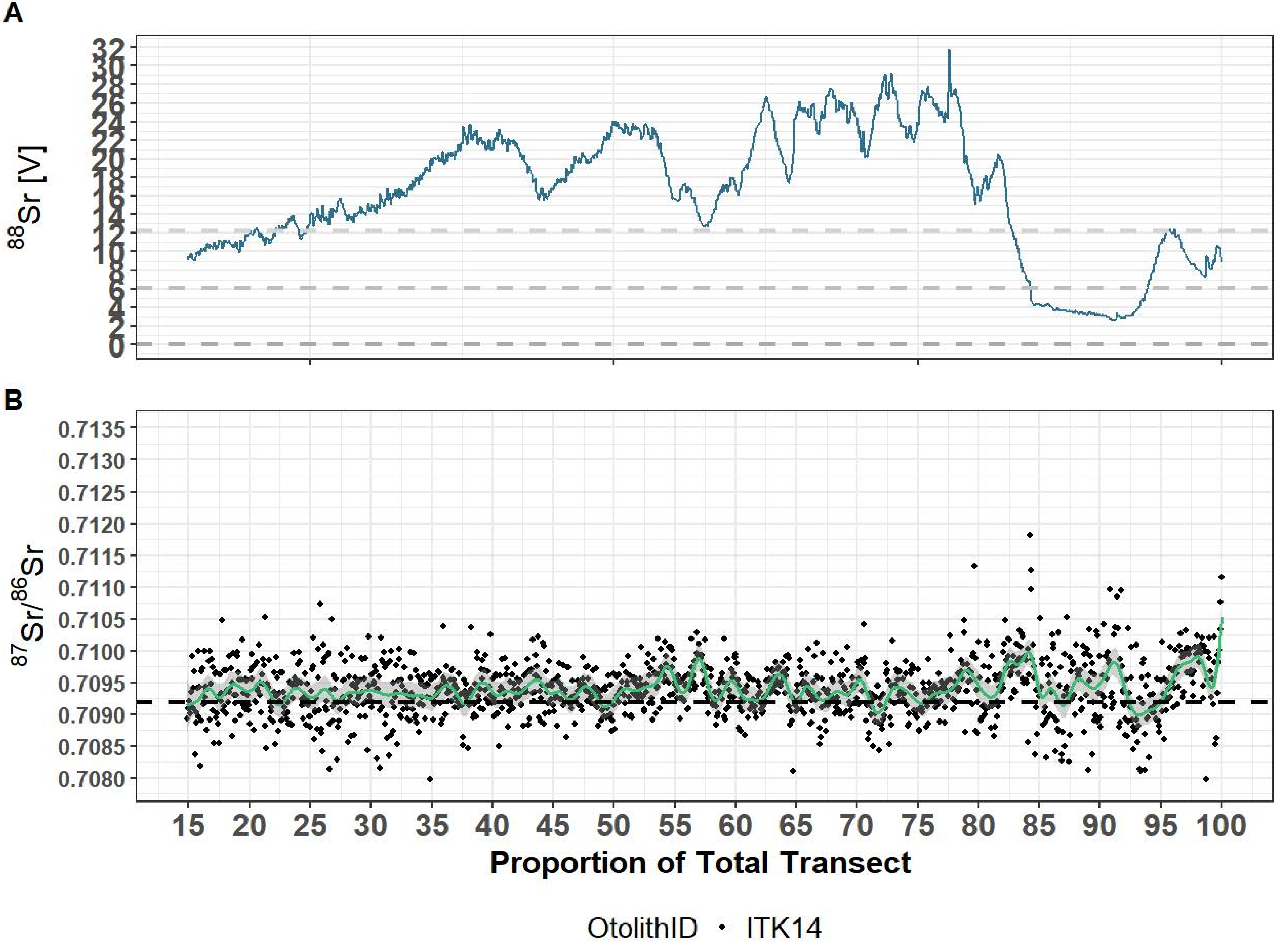

A
^{88}Sr [V]
B
^{87}Sr/^{86}Sr
0.7135
0.7130
0.7125
0.7120
0.7115
0.7110
0.7105
0.7100
0.7095
0.7090
0.7085
0.7080
15 20 25 30 35 40 45 50 55 60 65 70 75 80 85 90 95 100
Proportion of Total Transect
OtolithID • ITK14

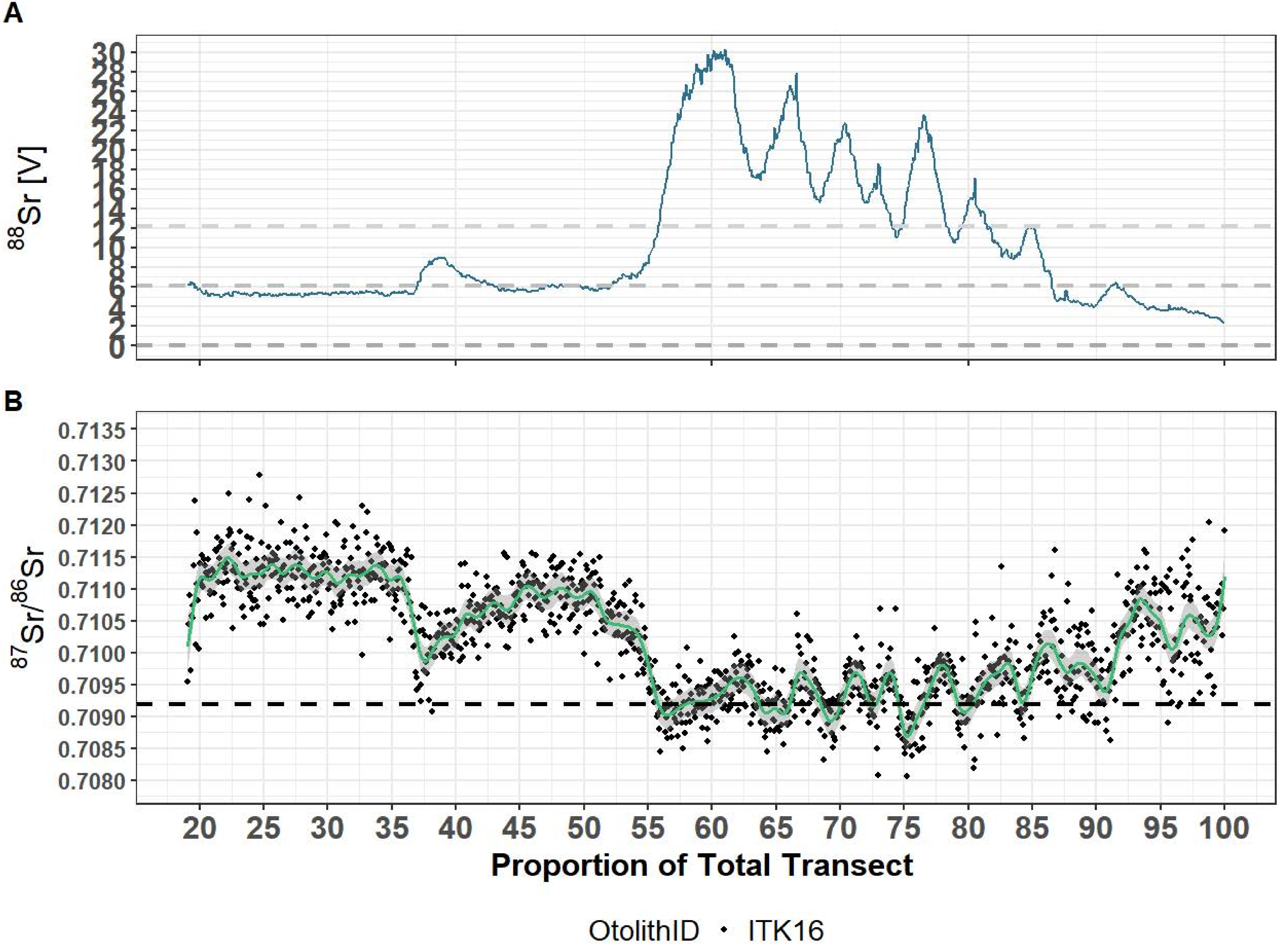

A
^{88}Sr [V]
0
2
4
6
8
10
12
14
16
18
20
22
24
26
28
30
B
^{87}Sr/^{86}Sr
0.7080
0.7085
0.7090
0.7095
0.7100
0.7105
0.7110
0.7115
0.7120
0.7125
0.7130
0.7135
20
25
30
35
40
45
50
55
60
65
70
75
80
85
90
95
100
Proportion of Total Transect
OtolithID • ITK16

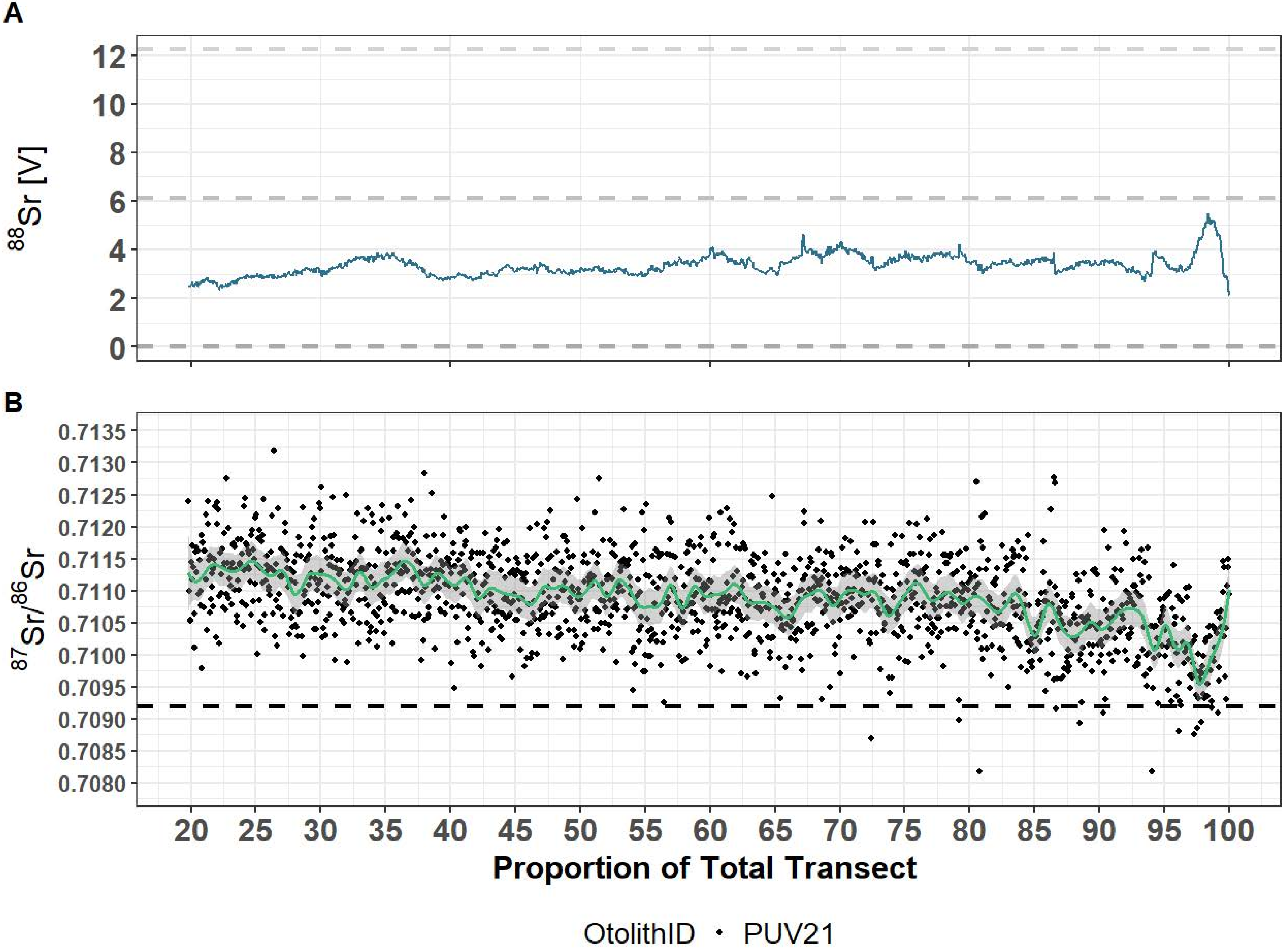

A
B
^{88}Sr [V]
12
10
8
6
4
2
0
$^{87}Sr/^{86}Sr$
0.7135
0.7130
0.7125
0.7120
0.7115
0.7110
0.7105
0.7100
0.7095
0.7090
0.7085
0.7080
20
25
30
35
40
45
50
55
60
65
70
75
80
85
90
95
100
Proportion of Total Transect
OtolithID • PUV21

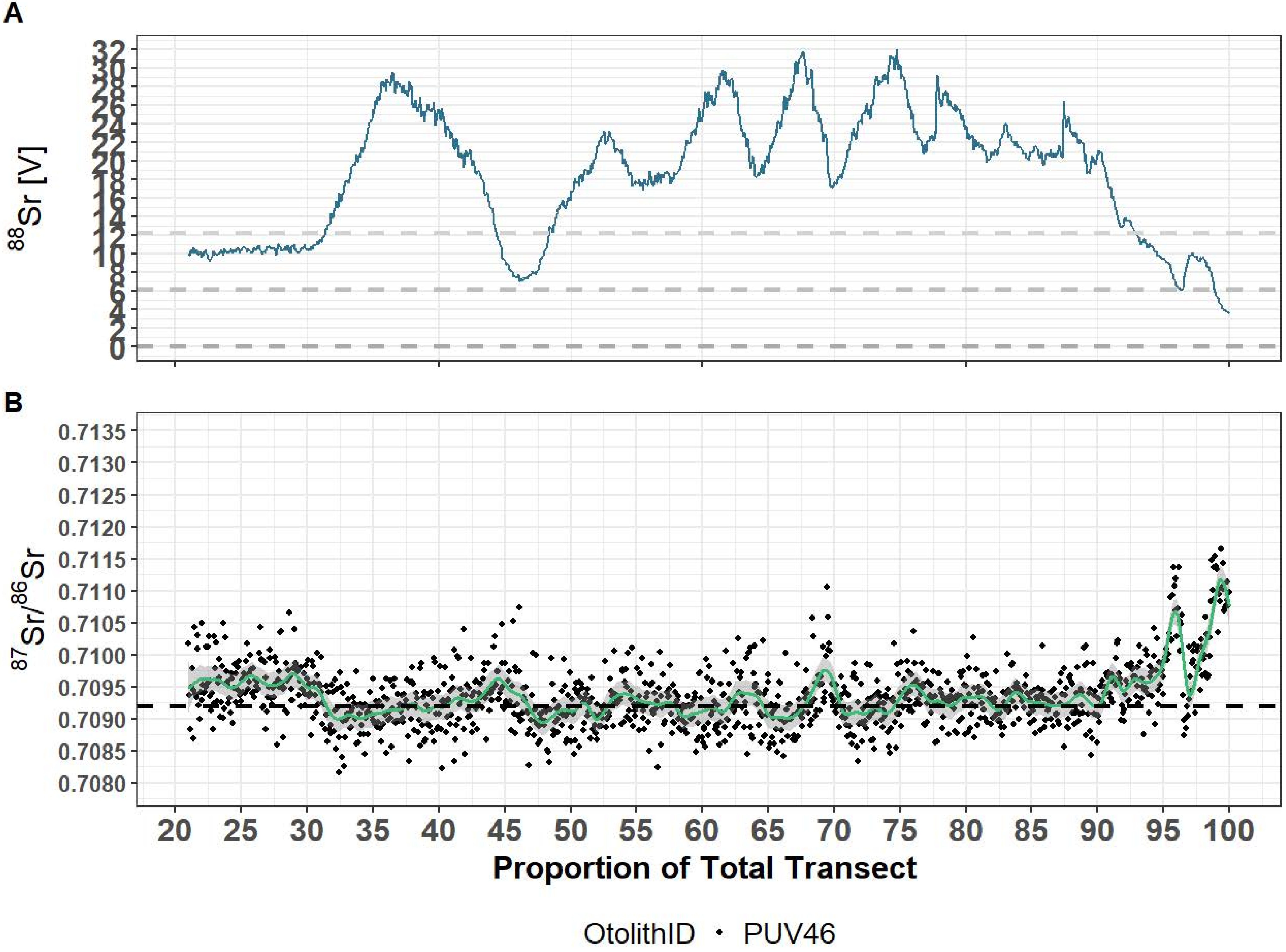

A
^{88}Sr [V]
32
30
28
26
24
22
20
18
16
14
12
10
8
6
4
2
0
B
^{87}Sr/^{86}Sr
0.7135
0.7130
0.7125
0.7120
0.7115
0.7110
0.7105
0.7100
0.7095
0.7090
0.7085
0.7080
20 25 30 35 40 45 50 55 60 65 70 75 80 85 90 95 100
Proportion of Total Transect
OtolithID • PUV46

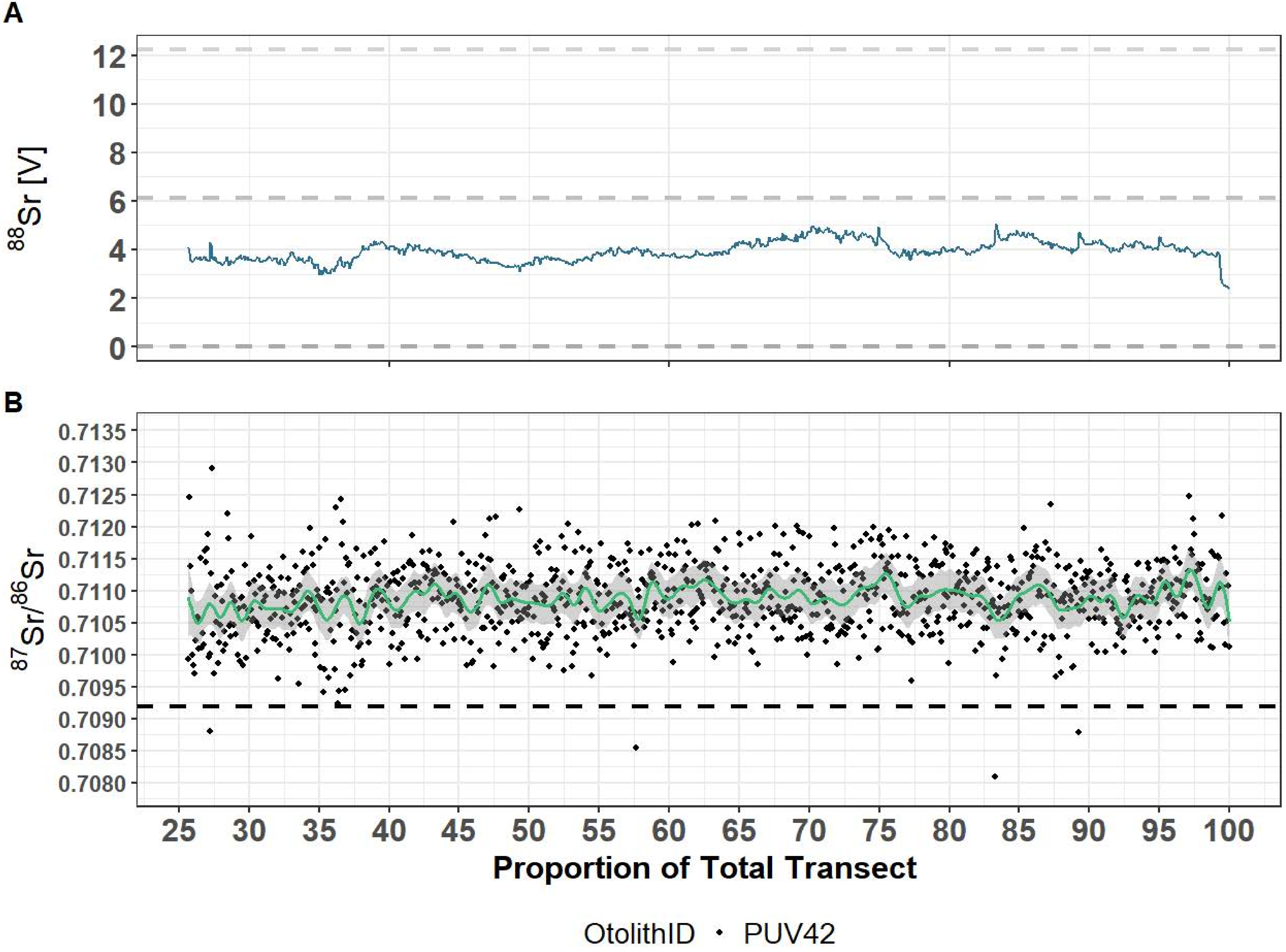

A
12
10
8
6
4
2
0
^{88}Sr [V]
B
0.7135
0.7130
0.7125
0.7120
0.7115
0.7110
0.7105
0.7100
0.7095
0.7090
0.7085
0.7080
^{87}Sr/^{86}Sr
25 30 35 40 45 50 55 60 65 70 75 80 85 90 95 100
Proportion of Total Transect
OtolithID • PUV42

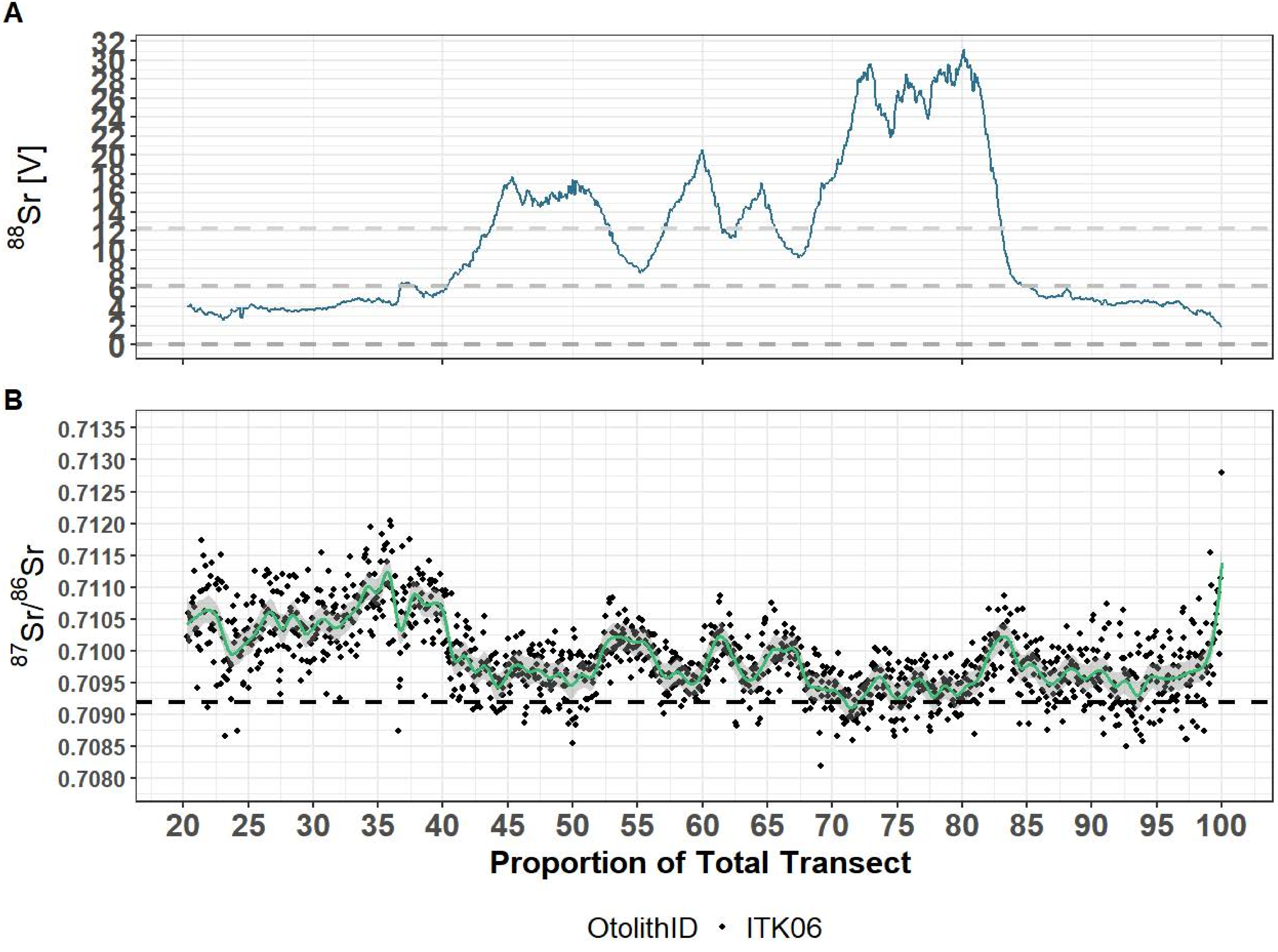
A
^{88}Sr [V]
0
2
4
6
8
10
12
14
16
18
20
22
24
26
28
30
32
B
^{87}Sr/^{86}Sr
0.7080
0.7085
0.7090
0.7095
0.7100
0.7105
0.7110
0.7115
0.7120
0.7125
0.7130
0.7135
20
25
30
35
40
45
50
55
60
65
70
75
80
85
90
95
100
Proportion of Total Transect
OtolithID • ITK06

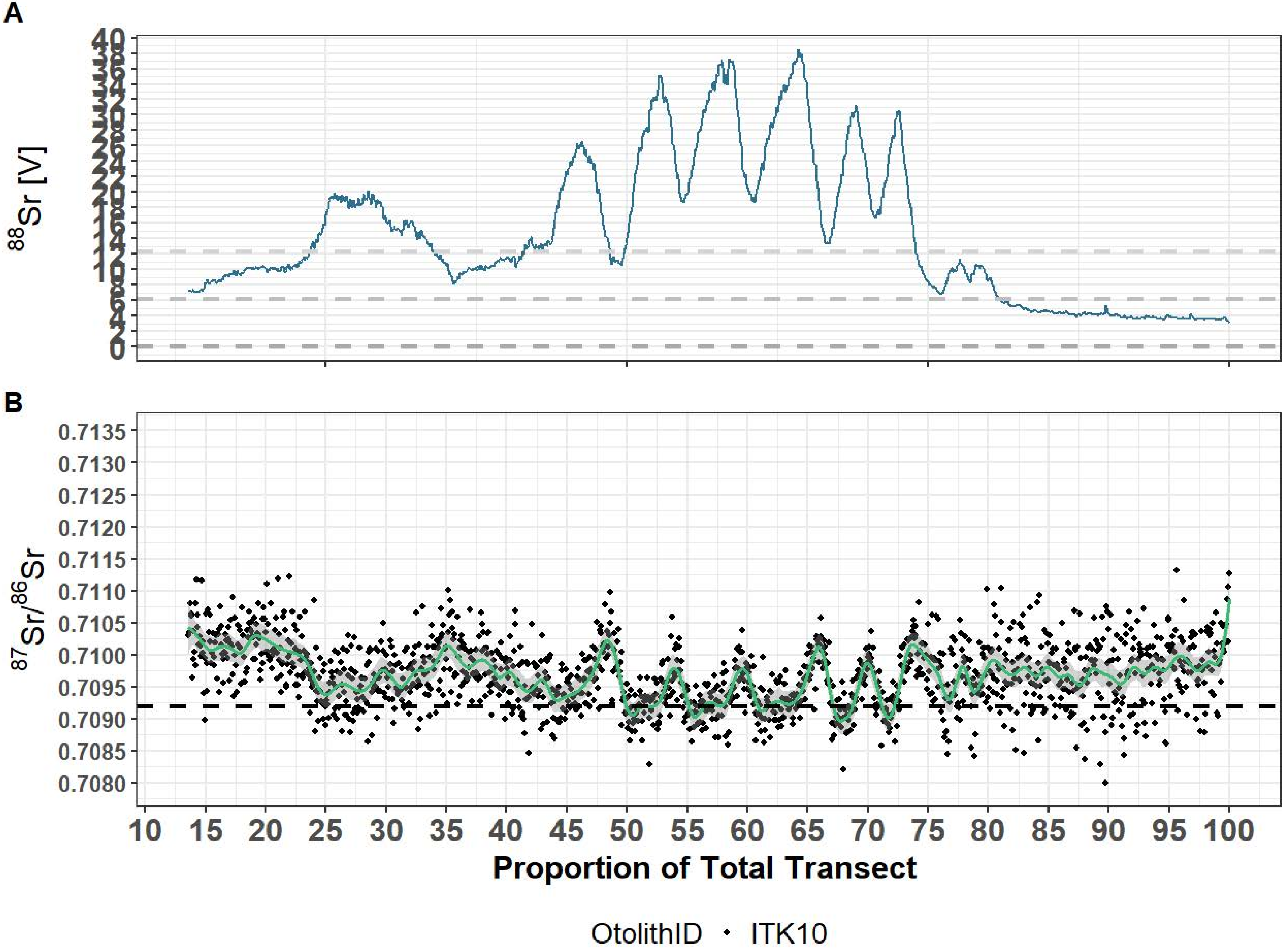
A
88Sr [V]
B
87Sr/86Sr
0.7135
0.7130
0.7125
0.7120
0.7115
0.7110
0.7105
0.7100
0.7095
0.7090
0.7085
0.7080
10 15 20 25 30 35 40 45 50 55 60 65 70 75 80 85 90 95 100
Proportion of Total Transect
OtolithID • ITK10

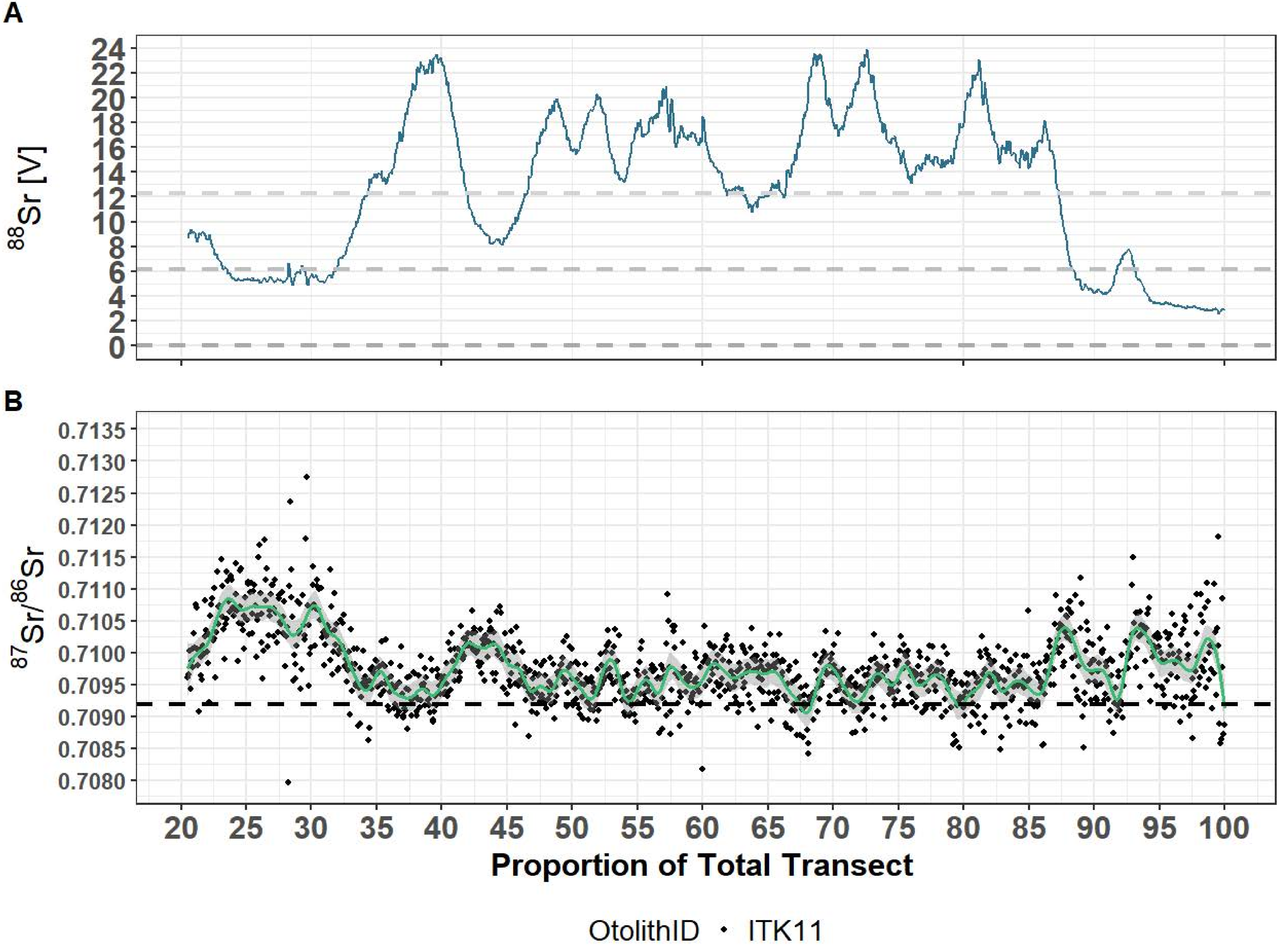

A
B
^{88}Sr [V]
^{87}Sr/^{86}Sr
Proportion of Total Transect
OtolithID • ITK11

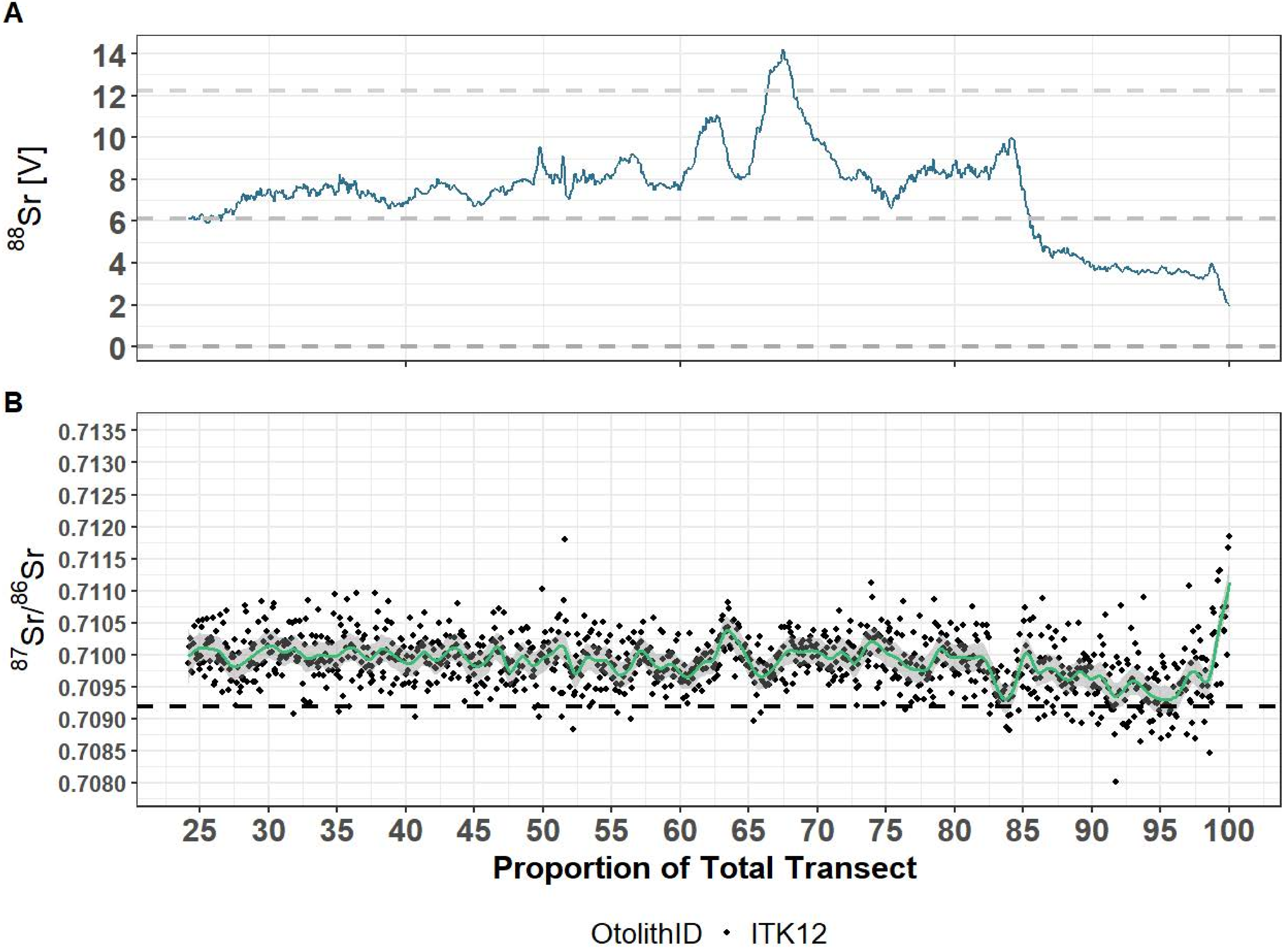

A
14
12
10
8
6
4
2
0
^{88}Sr [V]
B
0.7135
0.7130
0.7125
0.7120
0.7115
0.7110
0.7105
0.7100
0.7095
0.7090
0.7085
0.7080
$^{87}Sr/^{86}Sr$
25 30 35 40 45 50 55 60 65 70 75 80 85 90 95 100
Proportion of Total Transect
OtolithID • ITK12

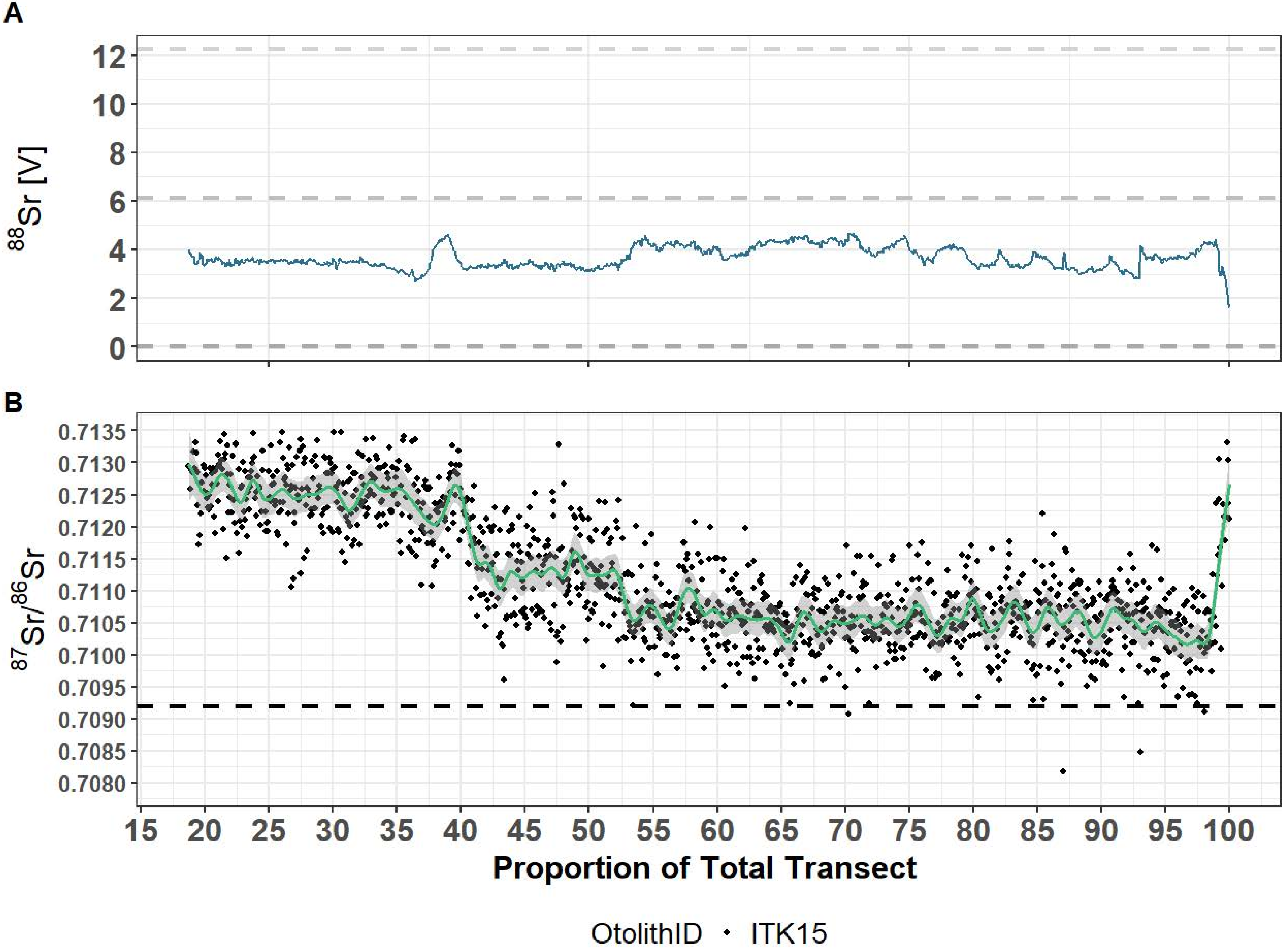
A
^{88}Sr [V]
0
2
4
6
8
10
12
B
$^{87}Sr/^{86}Sr$
0.7080
0.7085
0.7090
0.7095
0.7100
0.7105
0.7110
0.7115
0.7120
0.7125
0.7130
0.7135
15 20 25 30 35 40 45 50 55 60 65 70 75 80 85 90 95 100
Proportion of Total Transect
OtolithID ITK15

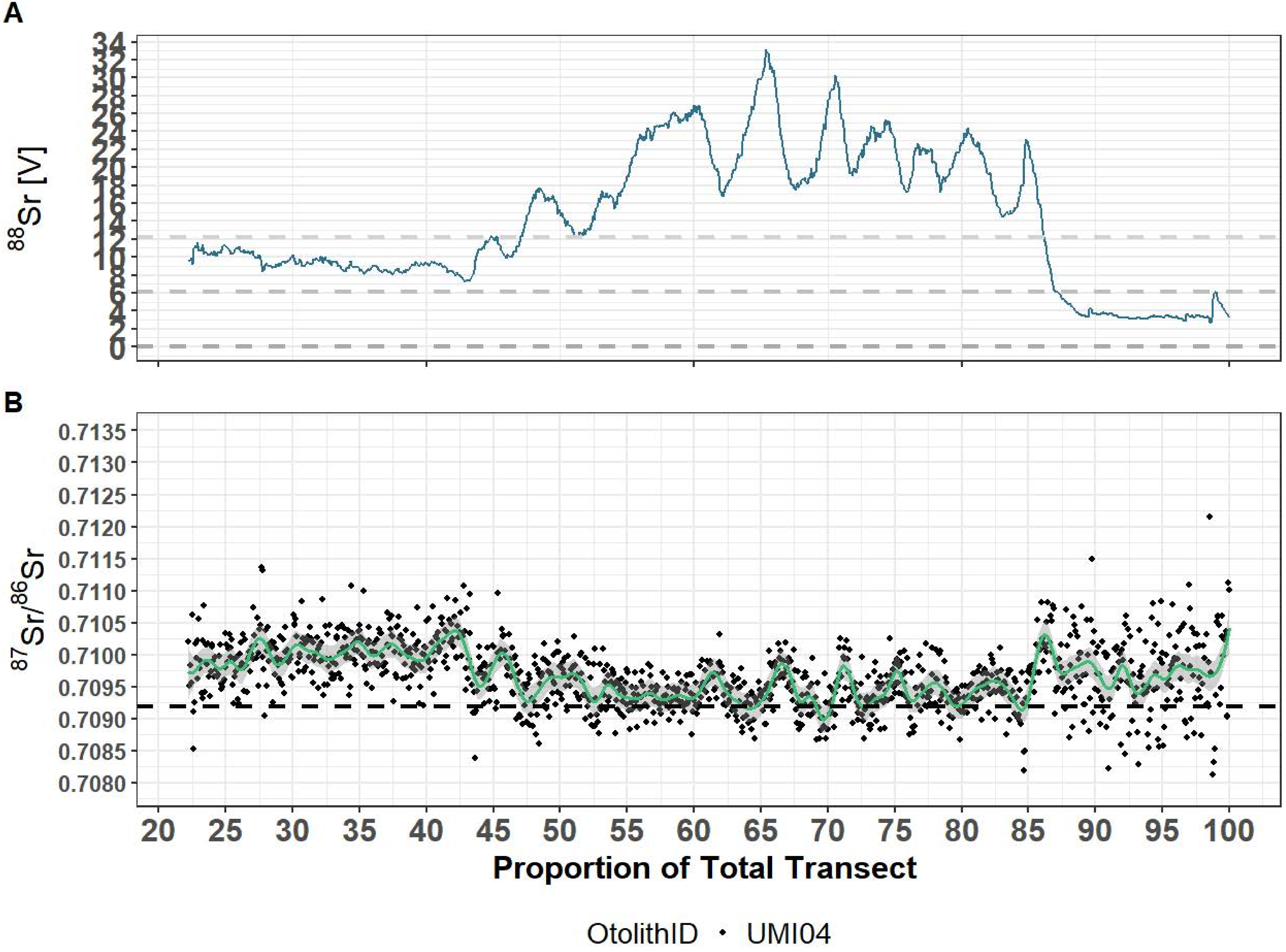

A
B
^{88}Sr [V]
0
2
4
6
8
10
12
14
16
18
20
22
24
26
28
30
32
34
$^{87}Sr/^{86}Sr$
0.7080
0.7085
0.7090
0.7095
0.7100
0.7105
0.7110
0.7115
0.7120
0.7125
0.7130
0.7135
20
25
30
35
40
45
50
55
60
65
70
75
80
85
90
95
100
Proportion of Total Transect
OtolithID • UMI04

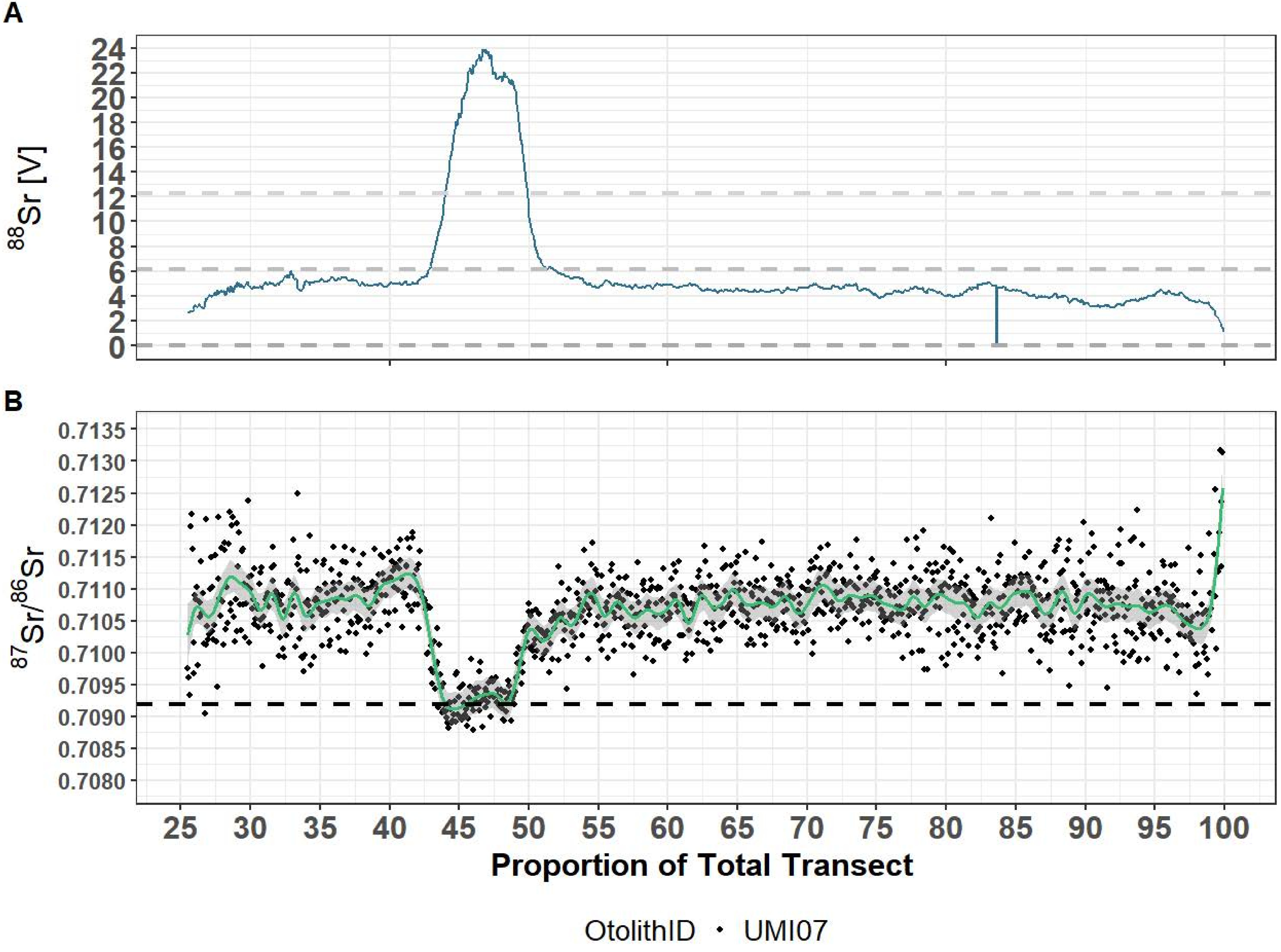

A
B
^{88}Sr [V]
$^{87}Sr/^{86}Sr$
Proportion of Total Transect
OtolithID • UMI07

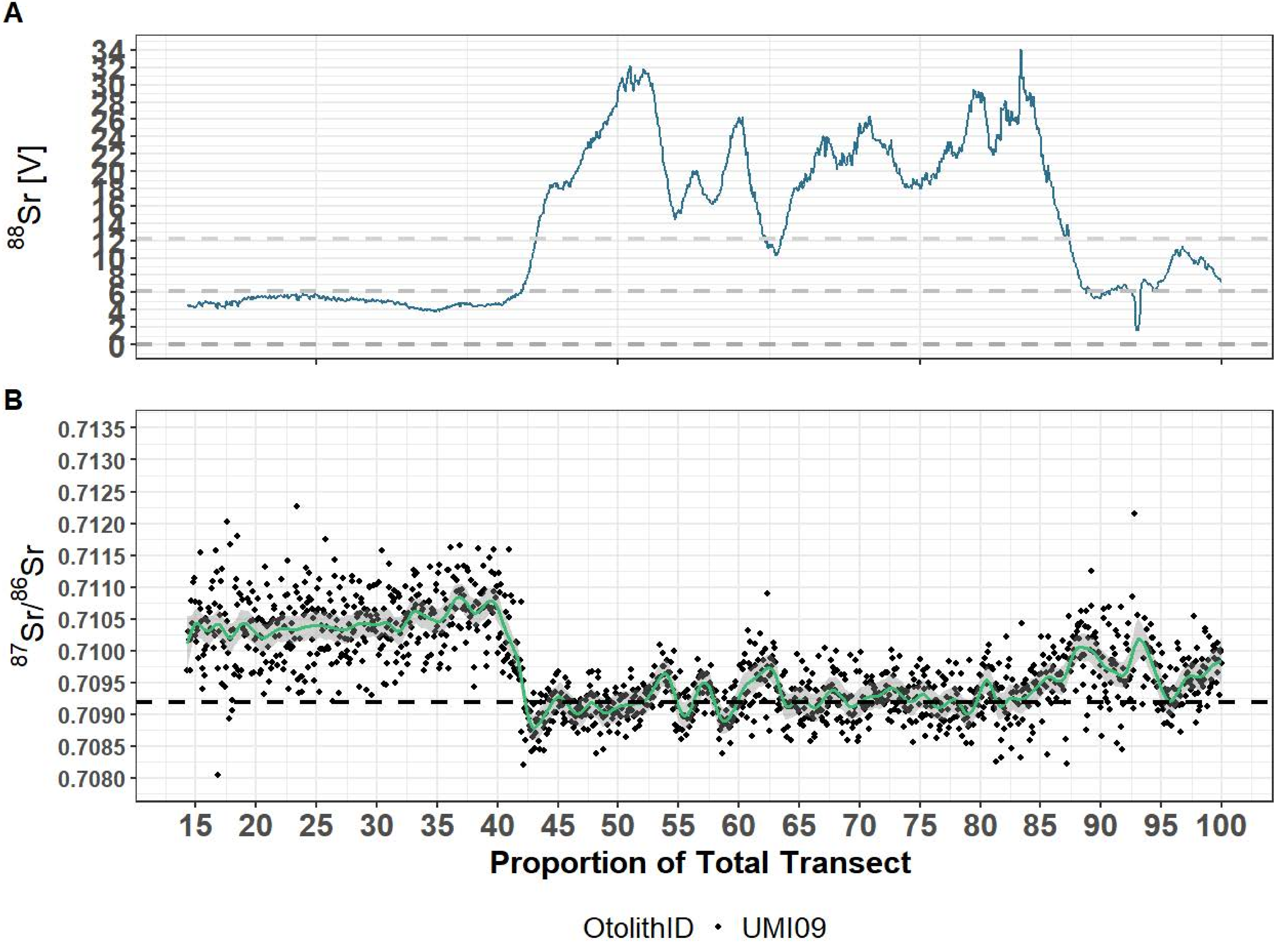

A
^{88}Sr [V]
34
32
30
28
26
24
22
20
18
16
14
12
10
8
6
4
2
0
B
^{87}Sr/^{86}Sr
0.7135
0.7130
0.7125
0.7120
0.7115
0.7110
0.7105
0.7100
0.7095
0.7090
0.7085
0.7080
15 20 25 30 35 40 45 50 55 60 65 70 75 80 85 90 95 100
Proportion of Total Transect
OtolithID • UMI09

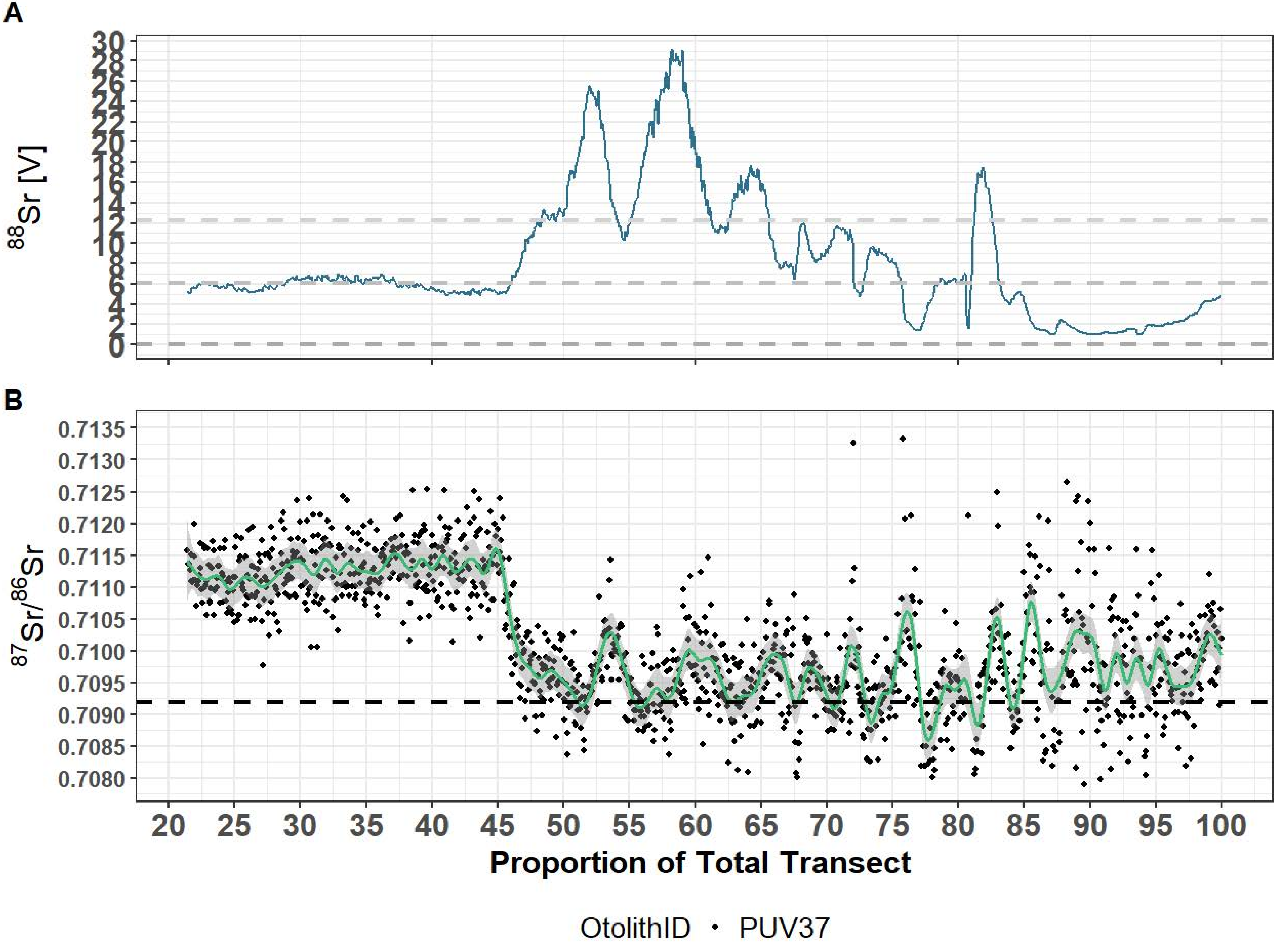
A
B
88Sr [V]
87Sr/86Sr
Proportion of Total Transect
OtolithID • PUV37

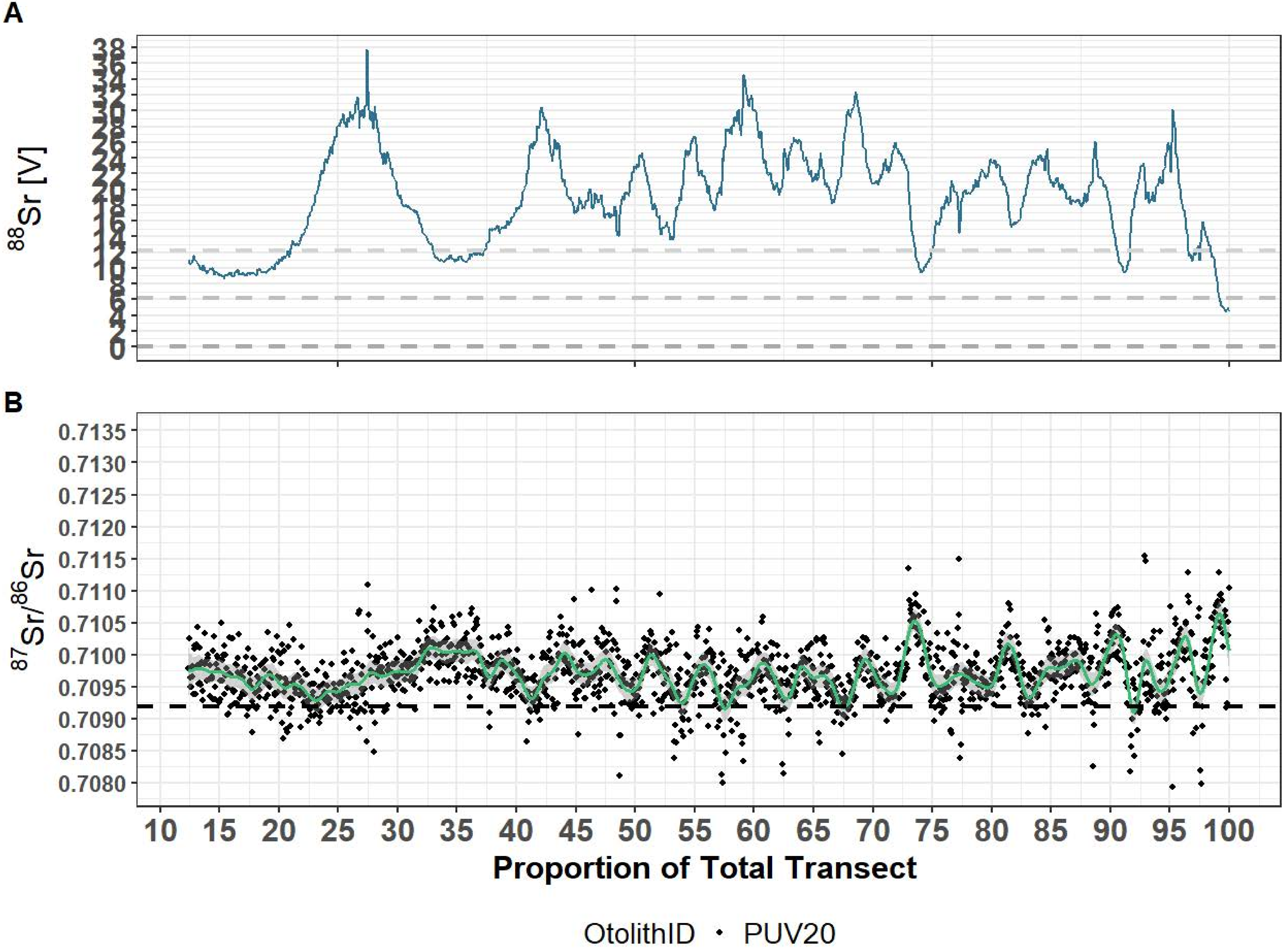

A
B
88Sr [V]
87Sr/86Sr
0.7135
0.7130
0.7125
0.7120
0.7115
0.7110
0.7105
0.7100
0.7095
0.7090
0.7085
0.7080
10
15
20
25
30
35
40
45
50
55
60
65
70
75
80
85
90
95
100
Proportion of Total Transect
OtolithID • PUV20
